# Synthesis and Cytotoxic Activity of Chiral Sulfonamides Based on the 2-Azabicycloalkane Skeleton

**DOI:** 10.3390/molecules25102355

**Published:** 2020-05-18

**Authors:** Mahzeiar Samadaei, Matthias Pinter, Daniel Senfter, Sibylle Madlener, Nataliya Rohr-Udilova, Dominika Iwan, Karolina Kamińska, Elżbieta Wojaczyńska, Jacek Wojaczyński, Andrzej Kochel

**Affiliations:** 1 Department of Internal Medicine III, Medical University of Vienna, AKH Vienna Währinger Gürtel 18-20, 1090 Vienna, Austria; mahzeiar.samadaei@meduniwien.ac.at (M.S.); matthias.pinter@meduniwien.ac.at (M.P.); daniel.senfter@meduniwien.ac.at (D.S.); sibylle.madlener@meduniwien.ac.at (S.M.); 2 Faculty of Chemistry, Wrocław University of Science and Technology, Wybrzeże Wyspiańskiego 27, 50-370 Wrocław, Poland; dominika.iwan@pwr.edu.pl (D.I.); karolina.kaminska@pwr.edu.pl (K.K.); 3 Faculty of Chemistry, University of Wrocław, F. Joliot-Curie St. 14, 50-383 Wrocław, Poland; jacek.wojaczynski@chem.uni.wroc.pl (J.W.); andrzej.kochel@chem.uni.wroc.pl (A.K.)

**Keywords:** 2-azabicycloalkane, chiral sulfonamide, cytotoxic activity

## Abstract

A series of chiral sulfonamides containing the 2-azabicycloalkane scaffold were prepared from *aza*-Diels–Alder cycloadducts through their conversion to amines based on 2-azanorbornane or the bridged azepane skeleton, followed by the reaction with sulfonyl chlorides. The cytotoxic activity of the obtained bicyclic derivatives was evaluated using human hepatocellular carcinoma (HCC), medulloblastoma (MB), and glioblastoma (GBM) cell lines. Chosen compounds were shown to notably reduce cell viability as compared to nonmalignant cells.

## 1. Introduction

**Figure 1:**
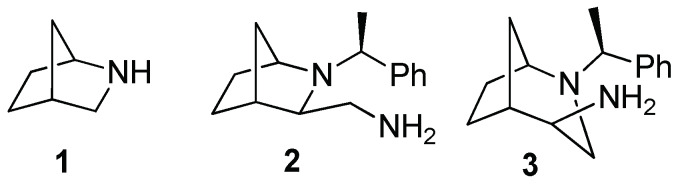
The 2-azanorbornane (**1**) and isomeric amines containing bicyclic skeletons (**2**,**3**).

A continuous demand for novel, potent, and selective anticancer drugs has led to the development of various classes of therapeutics. Among them, sulfonamides have attracted considerable attention due to their high and multidirectional activity, and are used or tested as pharmaceutical agents, possessing antibacterial, antiviral, anticancer, antimalarial, diuretic, hypoglycemic, anticonvulsant, antirheumatic, and antithyroid properties [1,2,3,4,5]. In addition to functional groups, certain characteristic structural motifs are also associated with impressive biological activity. For example, the biphenyl moiety is present in numerous derivatives that have been tested in antiproliferative studies [6,7,8]. The trifluoromethyl group is also regarded as a valuable pharmacophore [9], which is found in marketed drugs (e.g., nonsteroidal antiandrogen flutamide) and newly developed derivatives [10]. Additionally, 2-azanorbornane (2-azabicyclo[2.2.1]heptane) **1** (Figure 1), an intrinsically chiral compound with a stable bicyclic skeleton, has been recognized as a versatile scaffold for asymmetric synthesis and a precursor or a component of molecules exhibiting biological activity [11]. This system can be prepared via the stereoselective *aza*-Diels–Alder reaction of chiral imines and cyclopentadiene in both enantiomeric forms, even in large quantities (grams or kilograms) [12,13,14,15,16,17]. Derivatives of 2-azanorbornane have already been applied in various enantioselective transformations [11,18]. An expanded bicyclic system, namely 2-azabicyclo[3.2.1]octane, can be prepared in a stereoselective manner from 2-aznanorbornylmethanols under nucleophilic substitution reaction conditions [19]. Using this approach, isomeric amines based on the bridged piperidine (compound **2**, Figure 1) or azepane (isomer **3**, Figure 1) were prepared [20,21]. We used these amines and their epimers for the construction of bifunctional catalysts [22]. Monomeric and dimeric derivatives modified with amino functions were also prepared and were found to inhibit the growth of selected tumor cell lines [7].

The promising results of our study on the biological activity of amines bearing chiral azabicyclic skeletons prompted us to prepare a series of corresponding sulfonamides. Based on literature reports, we were convinced that introduction of the sulfonamide function, also with biphenyl substituents, should result in the enhancement of cytotoxicity and bioavailability. For example, combinations of sulfonamide and thiourea derivatives produced promising anticancer agents in the study by Ghorab et al. [2], while the methanesulfonamide analogue of cryptopleurine exhibited improved bioavailability and antitumor activity, as reported by Kwon et al. [4]. In this article, we present the synthesis of new bicyclic derivatives and the study of their interaction with selected human tumor cell lines and nonmalignant primary cells. We believed that molecules that contain both sulfonamide and biphenyl or trifluoromethyl moieties could lead to new hybrid architectures with improved biological profiles.

## 2. Materials and Methods

### 2.1. Instrumentation

Melting points were determined on the Schmelzpunkt Bestimmer Apotec melting point apparatus (WEPA Apothekenbedarf GmbH & Co. KG., Hillscheid, Germany) using the standard open capillary. ^1^H and ^13^C NMR spectra were collected on Jeol 400yh (Joel Ltd., Tokyo, Japan), Bruker Avance III 500, and Bruker Avance II 600 instruments (Bruker, Billerica, MA, USA). NMR spectra recorded in CDCl_3_ were referenced to the respective residual ^1^H or ^13^C signals of the solvent; chemical shifts are expressed in ppm, and coupling constants in Hz. Infrared spectra (4000–400 cm^−1^) were collected on a Perkin Elmer 2000 FTIR spectrometer (PerkinElmer, Waltham, MA, USA). High-resolution mass spectra were collected using electrospray ionization on a Waters LCT Premier XE TOF instrument (Waters Corporation, Milford, MA, USA). Optical rotations were measured using an Model AA-5 automatic polarimeter (Optical Activity, Ltd., Ramsey, UK); [α]_D_ values are given in 10^−1^ deg cm^2^ g^−1^. Chromatographic separations were performed on silica gel 60 (70–230 mesh). Thin-layer chromatography was carried out using silica gel 60 precoated plates.

X-ray diffraction data for monocrystals were collected on an Xcalibur Ruby diffractometer [23] with MoK_α_ radiation at 80(2) K. The Cambridge Crystallographic Data Centre (CCDC) reference numbers for **12a** and **12d**: CCDC 1992131 (**12a**) and 1992390 (**12d**) (Supplementary data available from CCDC, 12 Union Road, Cambridge CB2, 1EZ, UK on request). The crystal structures were solved with direct methods in SHELXT and refined using the full-matrix method in SHELXL [24]. H atoms bonded to C atoms were generated using HFIX instruction with U_eq_ = 1.2–1.5U_eq_ (parent C atom). Basic crystallographic data are presented in Supplementary Table S1.

### 2.2. Preparation of Starting Compounds

The bicyclic amines, (1*S*,3*R*,4*R*)-2-[(*S*)-1-phenylethyl]-3-aminemethyl-2- azabicyclo[2.2.1]heptane **2** and (1*S*,4*S*,5*R*)-2-[(*S*)-1-phenylethyl]-4-amine-2- azabicyclo[3.2.1]octane **3**, were prepared as described previously [20,21]. The synthesis of enantiopure 2-azanorbornane amine derivatives containing two bicyclic units **8** and **9** was also reported by our group [21].

### 2.3. General Procedure for Sulfonamide Synthesis

The appropriate sulfonyl chloride (1.0 mmol) was added to a solution of primary amine **2** or **3** (0.23 g, 1.0 mmol) and powdered potassium hydroxide (0.10 g, 1.8 mmol) in a dry dichloromethane (15.0 mL), which was stirred at room temperature. After 24 h, the resulting reaction mixture was poured over brine and extracted with dichloromethane. The organic phases were dried over Na_2_SO_4_, filtered through the Celite^®^, washed with dichloromethane, and evaporated under vacuum. The resulting residue was purified by column chromatography on silica (eluent: ethyl acetate/*n*-hexane 1:1 v/v), yielding sulfonamide **10** (from amine **2**) or **11** (from amine **3**). An analogous procedure was applied to the synthesis of compound **13a** from amine **9** (0.44 g, 1.0 mmol) and biphenyl-4-sulfonyl chloride (0.25 g, 1.0 mmol); the *n*-hexane/ethyl acetate (3/1 v/v) mixture was used for elution of the product. For synthesis of compounds bearing two sulfonamide functions, **12**, bisamine **8** (0.48 g, 1.0 mmol) was reacted with 2.0 mmol of the appropriate sulfonyl chloride; final products were eluted with *n*-hexane/ethyl acetate (3/1 v/v for 12a, 1/1 v/v for the remaining derivatives). The physicochemical characterization of all newly obtained compounds (including copies of ^1^H and ^13^C NMR spectra, and X-ray structures of compounds **12a** and **12d**) can be found in the Supplementary Materials.

### 2.4. Cell Lines and Culture Cconditions

The human hepatocellular carcinoma (HCC) cell line HUH7 was purchased from ATCC (LGC standards GmbH, Wesel, Germany). The Austrian human HCC cell line AKH12 has been well characterized [21,22,23,24,25] and was kindly provided by Prof. Bettina Grasl-Kraupp. HUH7 cells were cultured in Dulbecco’s modified Eagle’s medium (Thermo Fisher Scientific, Waltham, MA, USA) containing 10% heat-inactivated fetal bovine serum (FBS) (Sigma-Aldrich, St. Louis, MO, USA), 1% penicillin-streptomycin (10,000 U/mL) (Thermo Fisher Scientific, Waltham, MA, USA), and 1% minimum essential medium nonessential amino acids (100x) (Thermo Fisher Scientific, Waltham, MA, USA). AKH12 cells were cultured in RPMI1640 medium (Sigma-Aldrich, St. Louis, MO, USA) containing 10% heat-inactivated FBS. DAOY and D283Med (short D283) medulloblastoma (MB) cells were purchased from ATCC (LGC standards GmbH, Wesel, Germany). D425Med (short D425) and UW228-2 were provided by Thomas Ströbel from the Institute of Neurology Medical University of Vienna. The cells were cultured in Dulbecco’s modified Eagle’s medium (Sigma-Aldrich, St. Louis, MO, USA) containing 10% FBS (Sigma-Aldrich, St. Louis, MO, USA), 2% l-Glutamine (Sigma-Aldrich, St. Louis, MO, USA), and 0.2% Normocin (Sigma-Aldrich, St. Louis, MO, USA). U-251 MG (short U251) glioblastoma (GBM) cell line was purchased from Sigma-Aldrich and cultured in Dulbecco’s modified Eagle’s medium (Sigma-Aldrich, St. Louis, MO, USA) containing 10% FBS (Sigma-Aldrich, St. Louis, MO, USA), 2% l-Glutamine (Sigma-Aldrich, St. Louis, MO, USA), and 0.2% Normocin (Sigma-Aldrich, St. Louis, Missouri, USA). Human umbilical vein endothelial cells (HUVECs) were provided by Brigitte Winter from the Department of Surgery Medical University of Vienna and were grown in endothelial cell growth medium-2 BulletKitTM (Lonza, Basel, Switzerland) under standard tissue culture conditions. All cells were incubated at 37 °C in a 5% CO_2_ atmosphere and regularly checked for mycoplasma contamination.

### 2.5. Cell Viability Assay (Adherent Cell Lines)

Cell viability was determined using a neutral red assay, as previously described [26]. The cells were placed in 24-well plates and grown to reach 70–80% confluency prior to treatment. After treatment for 24 h, cells were incubated with neutral red at a concentration of 50 µg/mL in serum-free medium for 2 h at 37 °C. Cells were then washed 2x and incubated with 1% acetic acid in 70% ethanol. The dye concentration was measured photometrically at 562 nm. The percentage of survival was calculated considering DMSO-treated (indicated as 0 µM concentration) cells as 100% control. The final DMSO concentration in media did not exceed 0.2%.

LD_50_ was defined as the amount of a toxic agent required to eliminate 50% of the cell population. The LD_50_ was calculated in each experiment from the respective dose–response curve. All experiments were performed in triplicate wells.

### 2.6. Cell Viability Assay (Suspension Cell Line)

One day before treatment, 2 × 10^4^ of D425Med or HUVEC cells were seeded into 24-well plates and incubated at 37 °C in a humidified atmosphere containing 5% CO_2_.The cells were treated with different compounds for 24 h. A MTT (3-(4,5-dimethylthiazol-2-yl)-2,5-diphenyltetrazolium bromide) assay using the CellTiter Blue reagent (Promega, Fitchburg, Wisconsin, USA) was performed according to the manufacturer’s protocol. Experiments were performed in triplicate and repeated three times. The percentage of survival was calculated as the ratio using DMSO-treated (indicated as 0 µM concentration) cells as 100% control. The final DMSO concentration in media did not exceed 0.2%.

### 2.7. Determination of Lactate Dehydrogenase (LDH)

LDH release into culture media was measured by a cytotoxicity detection kit from Roche Diagnostics, according to the manufacturer’s instruction. LDH-positive controls or 100% lysed cells were generated in each cellular experiment by addition of Triton X100. Additionally, 0.2% DMSO was used as the vehicle control.

## 3. Results and Discussion

### 3.1. Preparation of Compounds

**Scheme 1:**
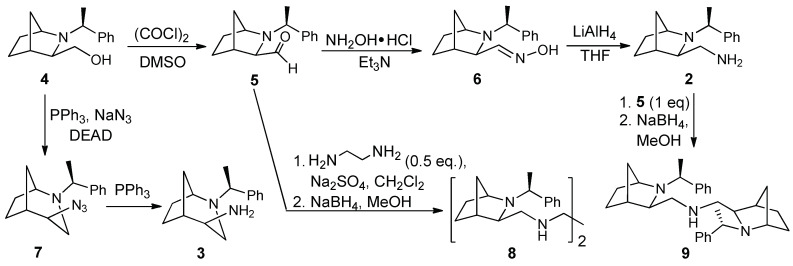
Synthesis of amines **2**, **3**, **8**, and **9**.

Enantiopure isomeric amines based on bicyclic skeletons of 2-azabicyclo[2.2.1]heptane 2 and 2-azabicyclo[3.2.1]octane **3** were prepared from (1*S*,3*R*,4*R*)-2-[(*S*)-1-phenylethyl]-3- hydroxymethyl-2-azabicyclo[2.2.1]heptane **4**, as previously described [19,21]. Nucleophilic substitution of the hydroxyl group of alcohol **4** with azide resulted in ring expansion, exclusively giving one stereoisomeric product **7**, which was then reduced with triphenylphosphine to yield amine **3** (Scheme 1). In turn, amine **2**, containing a preserved bicyclic system, was obtained using a three-step route involving Swern oxidation of alcohol **4**, conversion of the resulting aldehyde to oxime **6**, and its reduction. Amines containing two bicyclic fragments **8** and **9** were also reported in our previous paper [21]; their preparation included reaction of aldehyde **5** with ethylenediamine or amine **2**, respectively, followed by reduction of the obtained imines with sodium borohydride (Scheme 1).

**Scheme 2:**
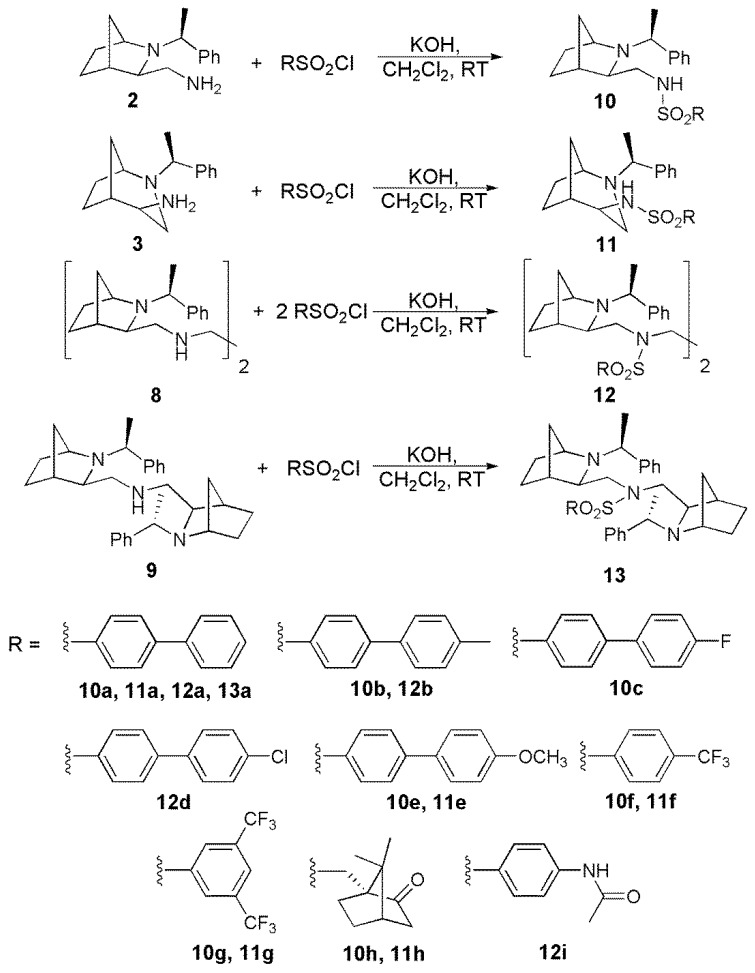
Synthesis of sulfonamides **10**–**13**.

Amines **2**, **3**, **8**, and **9** were converted into a series of sulfonamides. To this end, they were reacted with various sulfonyl chlorides in dichloromethane solution in the presence of powdered potassium hydroxide (Scheme 2). After chromatographic purification, they were obtained as crystalline solids or oils at 30–85% yield. Their structures were confirmed by spectroscopic methods (FTIR, ^1^H, and ^13^C NMR) and high-resolution mass spectrometry (data available in Supplementary Materials). In this way, enantiomerically pure derivatives **10** and **11**, differing in the size of the bicyclic skeleton and the distance of sulfonamide function from the chiral scaffold, were obtained together with compounds possessing two 2-azanorbornane subunits and one (**13**) or two (**12**) sulfonamide fragments. The introduced substituents included biphenyl (**10a-13a**), substituted biphenyl (**10b-10e**, **11e**, **12b**, and **12d**), trifluoromethylated phenyls (**10f-g**, **11f-g**), chiral camphoryl moiety (**10h**, **11h**), and acetamide derivative (**12i**, **13i**). The choice of these groups was substantiated by their presence in various drugs and therapeutics. Besides full spectroscopic characterization, structures of derivatives **12a** and **12d** were additionally confirmed by X-ray crystallography (structures shown in Supplementary Materials). We further investigated the impact of the sulfonamide structure on their cytotoxicity.

### 3.2. Primary Evaluation of the Anticancer Activity of Sulfonamide Compounds

All tested compounds were dissolved in dimethyl sulfoxide (DMSO). The solubility of compounds **12b**, **12d**, **8**, and **9** was not sufficient for our in vitro study. In the first screening, compounds that reduced cell viability by more than 50% at 50 µM after 24 h were considered as positive hints and were eligible for lactate dehydrogenase (LDH) cytotoxicity assay. To assess the anticancer activity of compounds, we tested human hepatocellular carcinoma (HCC), medulloblastoma (MB), and glioblastoma (GBM) cell lines. Further, to compare the effects of selected compounds on nonmalignant primary cells, we used primary human umbilical vein endothelial cells (HUVEC).

### 3.3. Sulfonamides and HCC Cell Lines

HCC accounts for a vast majority of liver cancer and is among the leading causes of cancer-related mortality [27]. HCC has been considered as a highly chemoresistant cancer, and hence is a good candidate for our study. The introduced 50µM concentration of **10a**, **10b**, **10c**, **10e**, **10f**, **10g**, **11a**, **11e**, **11g**, **12i**, and **13a** compounds reduced cell viability below LD_50_ in HUH7 and AKH12 cell lines (Table 1; Figures S1A and S3A available in Supplementary Materials). 50µM concentration of **11f**, however, only diminishes viability of HUH7 cells by more than 50%. The cytotoxicity of mentioned compounds was measured by the LDH release in a dose-dependent manner (Supplementary Figures S1B and S3B). The cytotoxicity was compared between HCC and HUVEC cells. Our results showed no significant differences of sulfonamide cytotoxicity in HCC cell as compared to HUVEC cells (Supplementary Figures S2 and S4—data available in Supplementary Materials). LD_50_ values for the above compounds tested in HUH7, AKH12, and HUVEC cells are summarized in Table 1.

### 3.4. Sulfonamides and MB Cell Lines

MB is considered the most common malignant brain tumor, with an estimated 5000–8000 cases per year worldwide [28]. Current MB classification defines four molecular subgroups, namely wingless (WNT), sonic hedgehog (SHH), group 3, and group 4 [29]. Among the MB cells tested, DAOY and UW228-2 belong to the SHH subgroup, whereas D425 corresponds to group 3. Cell line D283 exhibits features of both group 3 and group 4 [30]. Here, 50 µM of compounds **10a**, **10c**, **10e**, **10f**, **10g**, **11a**, **11e**, **11f**, **11g**, **12i**, and **13a** reduced cell viability in all 4 cell lines (Supplementary Figures S5A, S7A, S9A and S11A—data available in Supplementary Materials). Compound **10b,** however, only affected D425 and D283 cells, with LD_50_ values of 16.38 ± 2.34 µM and 31.27 ± 1.32 µM, respectively (Table 1 and Table 2). LDH release indicative for loss of membrane integrity is shown in Supplementary Figures S5B, S7B, S9B, and S11B. Among the compounds tested, **10b**, **10c**, **10e**, **10g**, and **11e** significantly reduced D425 cell viability as compared to HUVEC cells (* *p* ≤0.05, and ** *p* ≤0.01; Supplementary Figure S10—data available in Supplementary Materials). The LD_50_ values for the designated sulfonamides in all MB cell lines and HUVEC cells are shown in Table 1 and Table 2. LD_50_ values for compounds **10c** (5.38 µM) and **10g** (6.61 µM) in D425 cells (Table 2) were slightly below the LD_50_ values of the above known chemotherapeutics, etoposide, and cisplatin (8 and 10 µM, respectively) reported in the study by von Bueren et al. [31].

### 3.5. Sulfonamides and GBM Cell Line

Despite aggressive therapy, GBM continues to be associated with a dismal prognosis in virtually all adult patients [31]. U251, a GBM cell line, was subjected to 50µM of sulfonamides. Compounds **10a**, **10b**, **10c**, **10e**, **10f**, **10g**, **11a**, **11e**, **11f**, **11g**, **12i**, and **13a** decreased cell viability by more than 50% (Supplementary Figure S13A—data available in Supplementary Materials). The LDH (lactate dehydrogenase) cytotoxicity assay was performed to assess necrotic cell death (Supplementary Figure S13B—data available in Supplementary Materials). There were no statistically significant differences in cytotoxicity between U251 cells as compared to HUVEC cells (Supplementary Figure S14—data available in Supplementary Materials). Table 1 represents the LD_50_ values of the tested sulfonamide on U251 and HUVEC cell lines.

### 3.6. SAR Analysis

In our previous investigations, amines derived from 2-azanorbornane were prepared and tested for antiproliferative activity using three cancer cell lines: human lung cancer A549, human colon cancer HT-29, and biphenotypic leukemia MV4-11 [21]. The selectivity was assessed with human lung microvascular endothelial cells (HLMEC) and BALB/3T3 mouse embryo fibroblasts. Bisamine compounds 8 and 9, two compounds that can be treated as reference for the current study, exhibited rather low cytotoxicity, although for compound 8, the IC_50_ value for colon cancer line was comparable to cisplatin (7.2 ± 0.8 μM vs 9.7 ± 2.0 μM). The *N*-Benzyl derivative of compound 9 was more active (IC_50_ values for AT549, HT-29, and MV4-11 equaled 4.1 ± 0.1 μM, 4.8 ± 0.4 μM, and 5.4 ± 0.7 μM, respectively). However, it also inhibited the growth of mouse fibroblasts (IC_50_ =19.7 ± 10.1 μM) and nonmalignant endothelial cells (IC_50_ = 5.8 ± 0.6 μM), meaning its selectivity was unsatisfactory.

We planned to use compounds **8** and **9** as references in the current study. Unfortunately, they were found to be insufficiently soluble in the DMSO used for the tests. In contrast, most of the sulfonamide derivatives showed better solubility, and we could perform all tests with their samples. In our further studies, we plan to compare activities of all compounds using ethanol as the solvent.

Although the activities of the investigated sulfonamides varied from one cell line to another, certain regularities could be observed, which are important from the point of view of further developments. Comparison of activities exhibited by isomeric compounds **10** and **11**, containing bicyclic systems of various sizes, showed that 2-azanorbornyl derivatives (in particular, **10b**, **10c**, **10e**, and **10g**) exhibited higher cytotoxicity than their 2-azabicyclo[3.2.1.]octane (bridged azepane) counterparts. The scaffold of the latter can be regarded as less rigid; on the other hand, higher flexibility of the arm containing sulfonamide moiety in 2-azabicylo[2.2.1]heptane-derived compound **10** seems to be of importance as well. Among substituents present in the sulfonamide, biphenyl-containing methoxy group (**10e**, **11e**) or fluorine (**10c**) were found to be most active. For phenyl derivatives, the introduction of two –CF_3_ moieties resulted in enhanced activity and selectivity (compounds **10g**, **11g**), while one trifluoromethyl group in the *para* position was not sufficient to increase the antiproliferative properties.

Finally, dimeric species 12i and 13a were not particularly active, and even their toxicity for normal cell lines was higher than for malignant ones.

## 4. Conclusions

In this study, we evaluate the effect of chiral 2-azabicycloalkane-based sulfonamides on a variety of tumor cell lines, including HCC, MB, and GBM. Our results indicate that compounds **10b**, **10c**, **10e**, **10g**, and **11e** notably reduce cell viability on the D425 cell line (* *p* ≤ 0.05, and ** *p* ≤ 0.01) as compared to HUVEC cells. The D425 cell line is categorized as a group 3 medulloblastoma, with high MYC proto-oncogene amplification [29]. In D425, the best discrimination in cytotoxicity between malignant and nonmalignant cells was observed for compound **10c** (3.11-fold change), followed by compounds **10g** and **11e** (2.66- and 2.04-fold changes, respectively). Thus, compounds **10c**, **10g**, and **11e** showed the highest performance among the agents tested in this study. Although the safety margin seems to be too narrow and remains far below the >30-fold difference recommended for safety reasons [32], appropriate modifications can be introduced to optimize the structures of the studied sulfonamides. These promising stable compounds can serve as a convenient starting point for further exploration.

## Figures and Tables

**Table 1:** LD_50_ values for specified sulfonamides tested in Huh7, AKH12, DAOY, UW228-2, D283 and U251 tumor cell lines, as well as in human umbilical vein endothelial cells (HUVEC) cells.

			Cell Line/LD_50_ [µM]				
Compound	HCC		MB			GBM U251	HUVEC
	HUH7	AKH12	DAOY	UW228-2	D283		
10a	13.94 ± 1.02	15.5 ± 1.3	16 ± 1.12	28.8 ± 2.1	17.71 ± 1.09	13.51 ± 0.9	11.58 ± 1.11
10b	21.33 ± 1.23	23.72 ± 1.27	-	-	31.27 ± 1.32	20.44 ± 1.58	12.88 ± 2.13
10c	17.33 ±0.98	23.27 ± 2.1	16 ± 2.1	15.74 ± 1.32	27.25 ± 2.35	21.33 ± 1.01	13.8 ± 1.14
10e	16.33 ± 0.69	32 ± 2.21	15.54 ± 1.62	26.66 ± 1.52	15.61 ± 0.59	16 ± 2	9.58 ± 1.88
10f	24.57 ± 1.54	24.72 ± 1.98	15.77 ± 1.09	28 ± 2.09	18.78 ± 1.22	32.72 ± 1.32	17.26 ± 2.33
10g	11.66 ± 1.53	10.18 ± 1.09	19.2 ± 1.42	12 ± 0.99	16 ± 1	11.76 ± 1.58	10.77 ± 0.93
11a	22.5 ± 1.21	28.8 ± 1.53	18.66 ± 1.88	16 ± 1.03	20 ± 2.02	18.78 ± 2.11	13.35 ± 0.25
11e	30.22 ± 2.21	28 ± 1.39	24 ± 1.49	23.46 ± 1.56	25.9 ± 1.9	23.7 ± 1.69	17.58 ± 2.94
11f	42.66 ± 1.98	-	17.33 ± 0.99	31.36 ± 2.12	29.91 ± 1.5	31.48 ± 2	22.28 ± 1.45
11g	20 ± 1.1	28 ± 1.72	20.41 ± 1.33	20.14 ± 1.34	17.23 ± 0.89	24.8 ± 0.92	18.68 ± 1.97
12i	7.75 ± 0.84	5.75 ± 0.47	12.88 ± 1.59	28.44 ± 2.11	21.81 ± 1.9	15.33 ± 1.59	3.16 ± 0.15
13a	13.92 ± 0.49	8 ± 0.28	19.2 ± 1.37	25.9 ± 1.98	38.4 ± 1.11	27.63 ± 1.04	12.13 ± 0.26

**Table 2:** LD_50_ values for designated sulfonamides tested in D425 and HUVEC cells.

	Cell line/LD_50_ [µM]		
Compound	MB D425	HUVEC	HUVEC/D425 LD_50_ Fold Change
10a	16.71 ± 2.11	28 ± 1.86	1.67
10b	16.38 ± 2.34	28.9 ± 2.97	1.76
10c	5.38 ± 0.77	16.77 ± 0.71	3.11
10e	10.95 ± 0.47	11.54 ± 2.8	1.05
10f	20.24 ± 0.79	23.15 ± 2.21	1.14
10g	6.61 ± 1.52	17.62 ± 2.98	2.66
11a	21.42 ± 0.89	21.71 ± 1.99	1.01
11e	17.78 ± 1.04	36.38 ± 4.37	2.04
11f	32 ± 1.37	31.52 ± 1.16	0.98
11g	11.2 ± 1.73	16 ± 2.29	1.42
12i	15.6 ± 0.93	8.91 ± 2.11	0.57
13a	30.54 ± 1.86	16 ± 0.79	0.52

## Supplementary Materials

The following are available online. Figure S1: Effect of sulfonamide compounds on HUH7 cell line, Figure S2: Impact of sulfonamide compounds on HUH7 in contrast to HUVEC, Figure S3: Viability and cytotoxicity effect of sulfonamide compounds on AKH12 cells, Figure S4: Impact of sulfonamide compounds on AKH12 compared to HUVEC, Figure S5: Influence of sulfonamide compounds treatment on DAOY cell viability and cytotoxicity, Figure S6: Sulfonamide compounds sensitivity of DAOY in contrast to HUVEC cell lines, Figure S7: Effect of sulfonamides on viability of UW228-2 cell line, Figure S8: Impact of sulfonamides on UW228-2 in contrast to HUVEC, Figure S9: Sulfonamides sensitivity and cytotoxicity on D425 cells, Figure S10: Effect of sulfonamides on viability of D425 in comparison with HUVEC cell lines, Figure S11: Influence of sulfonamide compounds treatment on D283 cell viability and cytotoxicity, Figure S12: Efficacy of sulfonamides on cell viability of D283 compared to HEVEC cell lines, Figure S13: Sulfonamides and viability of U251 cells, Figure S14: Efficacy of sulfonamides on cell viability of U251 compared to HEVEC cell lines, Figure S15: X-Ray structure of bis-sulfonamide **12a**, Figure S16: X-Ray structure of bis-sulfonamide **12d**, Table S1: X-Ray data for compounds **12a** and **12d**; spectral data for new compounds and copies of ^1^H NMR and ^13^C NMR spectra.
