# Supplementary material for: Synthesis and Cytotoxic Activity of Chiral Sulfonamides Based on the 2-Azabicycloalkane Skeleton
Source: Molecules. 2020 May 18;25(10):2355. doi: 10.3390/molecules25102355 (PMC7288168; doi:10.3390/molecules25102355)
Supplement: Supplementary file 1 [file molecules-25-02355-s001.pdf]

Supporting Information

Synthesis and cytotoxic activity of chiral sulfonamides based on the 2-azabicycloalkane skeleton

Content

ANTIPROLIFERATIVE ACTIVITY STUDIES 3

Figure S1. Effect of sulfonamide compounds on HUH7 cell line 3

Figure S2. Impact of sulfonamide compounds on HUH7 in contrast to HUVEC. 4

Figure S3. Viability and cytotoxicity effect of sulfonamide compounds on AKH12 cells. 5

Figure S4. Impact of sulfonamide compounds on AKH12 compared to HUVEC 6

Figure S5. Influence of sulfonamide compounds treatment on DAOY cell viability and cytotoxicity 7

Figure S6. Sulfonamide compounds sensitivity of DAOY in contrast to HUVEC cell lines 8

Figure S7. Effect of sulfonamides on viability of UW228-2 cell line 9

Figure S8. Impact of sulfonamides on UW228-2 in contrast to HUVEC 10

Figure S9. Sulfonamides sensitivity and cytotoxicity on D425 cells 11

Figure S10. Effect of sulfonamides on viability of D425 in comparison with HUVEC cell lines 12

Figure S11. Influence of sulfonamide compounds treatment on D283 cell viability and cytotoxicity 13

Figure S12. Efficacy of sulfonamides on cell viability of D283 compared to HEVEC cell lines 14

Figure S13. Sulfonamides and viability of U251 cells 15

Figure S14. Efficacy of sulfonamides on cell viability of U251 compared to HEVEC cell lines 16

X-RAY STRUCTURES AND CRYSTALOGRAPHIC DATA OF SULFONAMIDES 12a AND 12d 17

Figure S15. X-Ray structure of bis-sulfonamide 12a. 17

Figure S16. X-Ray structure of bis-sulfonamide 12d. 17

SPECTRAL DATA 19

4-Phenyl-*N*-((2-((*S*)-1-phenylethyl)-(1*S*,3*R*,4*R*)-2-azabicyclo[2.2.1]heptane-3-yl) benzenesulfonamide (10a) 19

4-(4-Tolyl)-*N*-((2-((*S*)-1-phenylethyl)-(1*S*,3*R*,4*R*)-2-azabicyclo[2.2.1]heptane-3-yl)benzenesulfonamide (10b) 20

4-(4-Fluorophenyl)-*N*-((2-((*S*)-1-phenylethyl)-(1*S*,3*R*,4*R*)-2-azabicyclo[2.2.1]heptane-3-yl)benzenesulfonamide (10c) 21

4-(4-Metoxyphenyl)-*N*-((2-((*S*)-1-phenylethyl)-(1*S*,3*R*,4*R*)-2-azabicyclo[2.2.1]heptane-3-yl)benzenesulfonamide (10e) 23

4-Trifluoromethyl-*N*-((2-((*S*)-1-phenylethyl)-(1*S*,3*R*,4*R*)-2-azabicyclo[2.2.1]heptane-3-yl) benzenesulfonamide (10f) 24

3,5-Bis(trifluoromethyl)-*N*-((2-((*S*)-1-phenylethyl)-(1*S*,3*R*,4*R*)-2-azabicyclo[2.2.1]heptane-3-yl)benzenesulfonamide (10g) 26

N-((2-((S)-1-Phenylethyl)-(1S,3R,4R)-2-azabicyclo[2.2.1]heptan-3-yl)-[(1R)-(-)-10-camphor]sulfonamide (10h) 27

4-Phenyl-*N*-((2-((*S*)-1-phenylethyl)-(1*S*,4*S*,5*R*)-2-azabicyclo[3.2.1]octan-4-yl) benzenesulfonamide (11a) 29

4-(4-Methoxyphenyl)-*N*-((2-((*S*)-1-phenylethyl)-(1*S*,4*S*,5*R*)-2-azabicyclo[3.2.1]octan-4-yl)benzenesulfonamide (11e) 30

4-Trifluoromethyl-*N*-((2-((*S*)-1-phenylethyl)-(1*S*,4*S*,5*R*)-2-azabicyclo[3.2.1]octan-4-yl)benzenesulfonamide (11f) 32

3,5-Bis(trifluoromethyl)-N-((2-((S)-1-phenylethyl)-(1S,4S,5R)-2-azabicyclo[3.2.1]octan-4-yl)benzenesulfonamide (11g) 33

N-((2-((S)-1-Phenylethyl)-(1S,4S,5R)-2-azabicyclo[3.2.1]octan-4-yl)-[(1R)-(-)-10-camphore]sulfonamide (11h) 35

Bis-*N*,*N*-{[(1*S*,3*R*,4*R*)-2-((*S*)-1-phenylethyl)-2-azabicyclo[2.2.1]heptan-3-yl]methyl}-[1,1'-biphenyl]ethyl-4-sulfonamide (12a) 36

Bis-4'-methyl-N,N-{[(1*S*,3*R*,4*R*)-2-((*S*)-1-phenylethyl)-2-azabicyclo[2.2.1]heptan-3-yl]methyl}-[1,1'-biphenyl]ethyl-4-sulfonamide (12b) 38

Bis-4'-chloro-*N*,*N*-{[(1*S*,3*R*,4*R*)-2-((*S*)-1-phenylethyl)-2-azabicyclo[2.2.1]heptan-3-yl]methyl}-[1,1'-biphenyl]ethyl-4-sulfonamide (12d) 39

Bis-*N*,*N*-(4-(*N*-(((1*S*,3*R*,4*R*)-2-((*S*)-1-phenylethyl)-2-azabicyclo[2.2.1]heptan-3-yl)methyl)sulfamoyl)ethyl)phenyl)acetamide (12i) 41

N-Bis-(((1*S*,3*R*,4*R*)-2-((*S*)-1-phenylethyl)-2-azabicyclo[2.2.1]heptan-3-yl)methyl)-[1,1'-biphenyl]-4-sulfonamide (13a) 42

ANTIPROLIFERATIVE ACTIVITY STUDIES

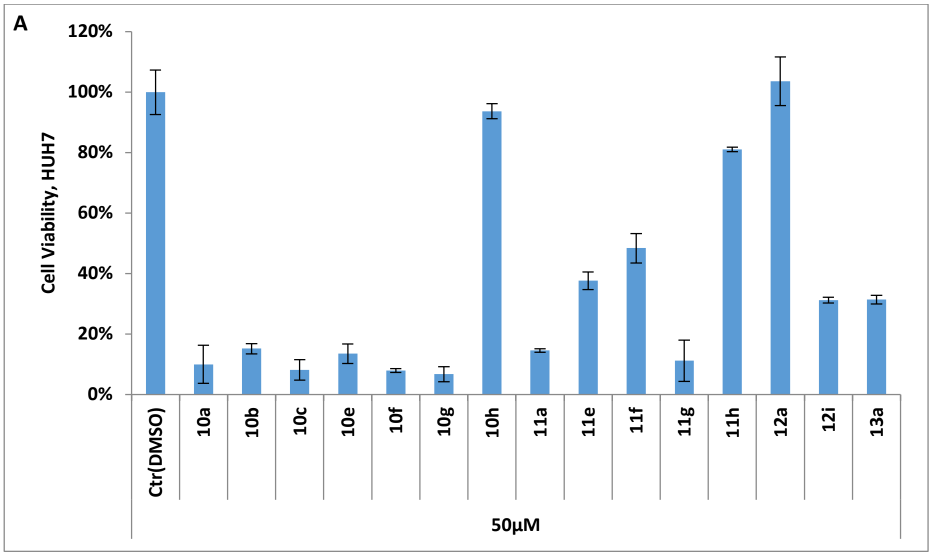

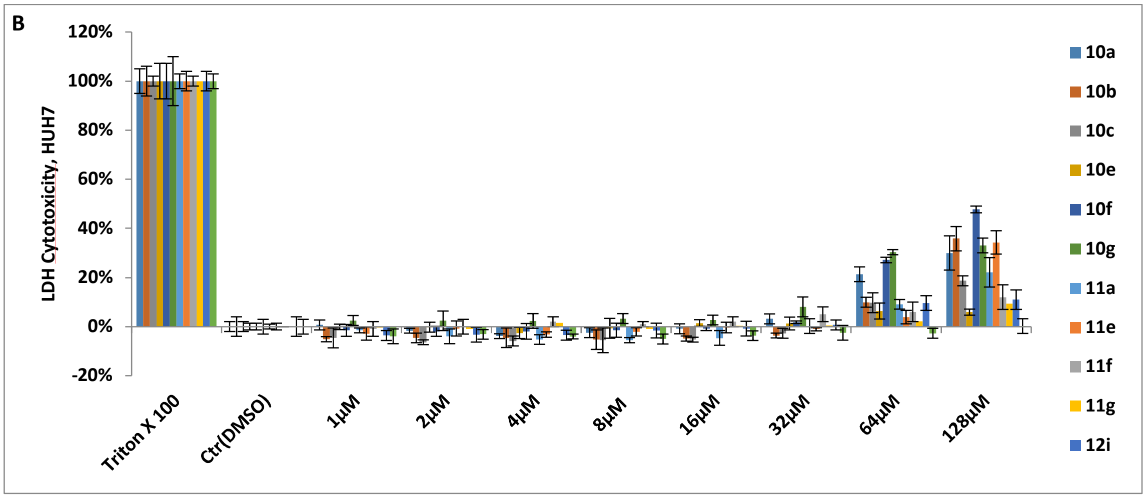

Figure S1. Effect of sulfonamide compounds on HUH7 cell line. A) HUH7 cell line was incubated with 50µM of sulfonamide compounds or vehicle control (DMSO) for 24 h. B) A quantitative measurement of LDH released into the media for tested compounds after 24 h. We used cell lysis by Triton X 100 as a 100% positive control. All experiments were performed in triplicates, error bars indicate ± SD.

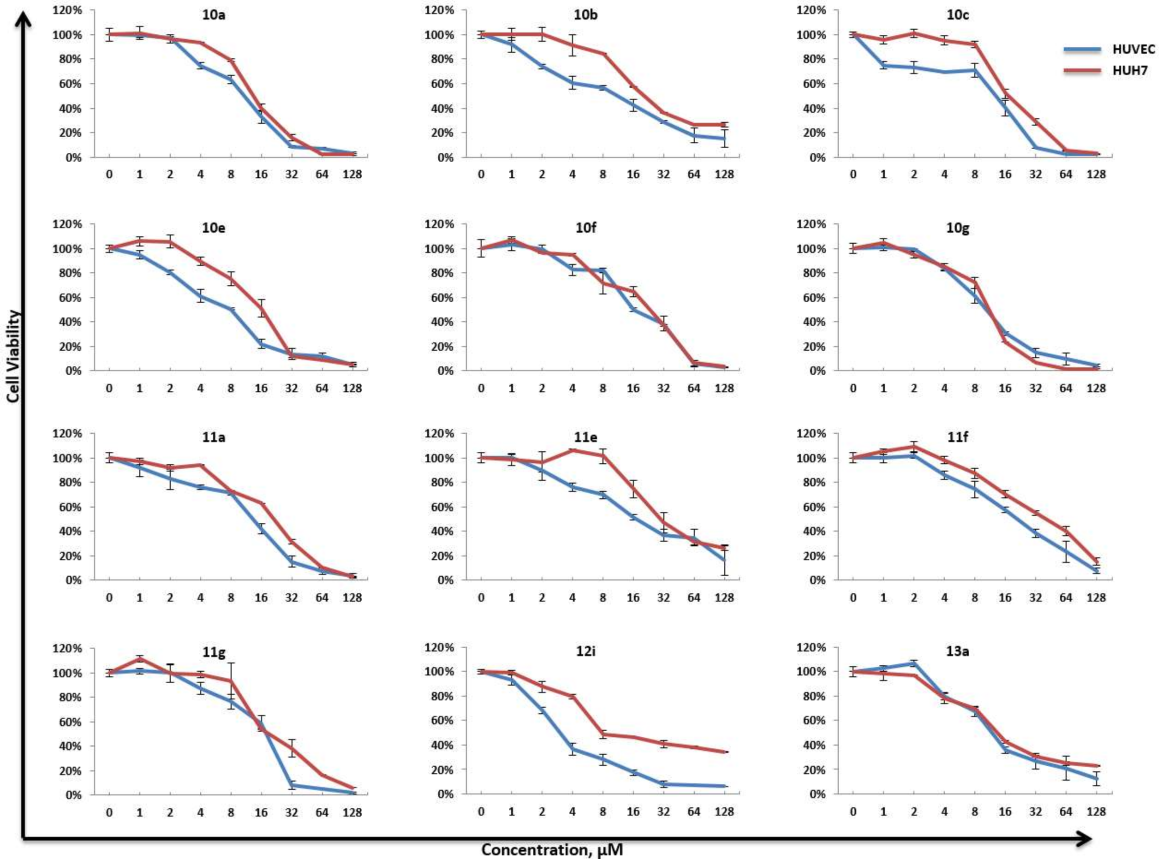

Figure S2. Impact of sulfonamide compounds on HUH7 in contrast to HUVEC. After 24 h treatment with various concentration of sulfonamides (1, 2, 4, 8, 16, 32, 64, 128 μM) cell viability of HUH7 and in cells compared to HUVEC cells was measured. Values are the average ±SD of triplicate experiments.

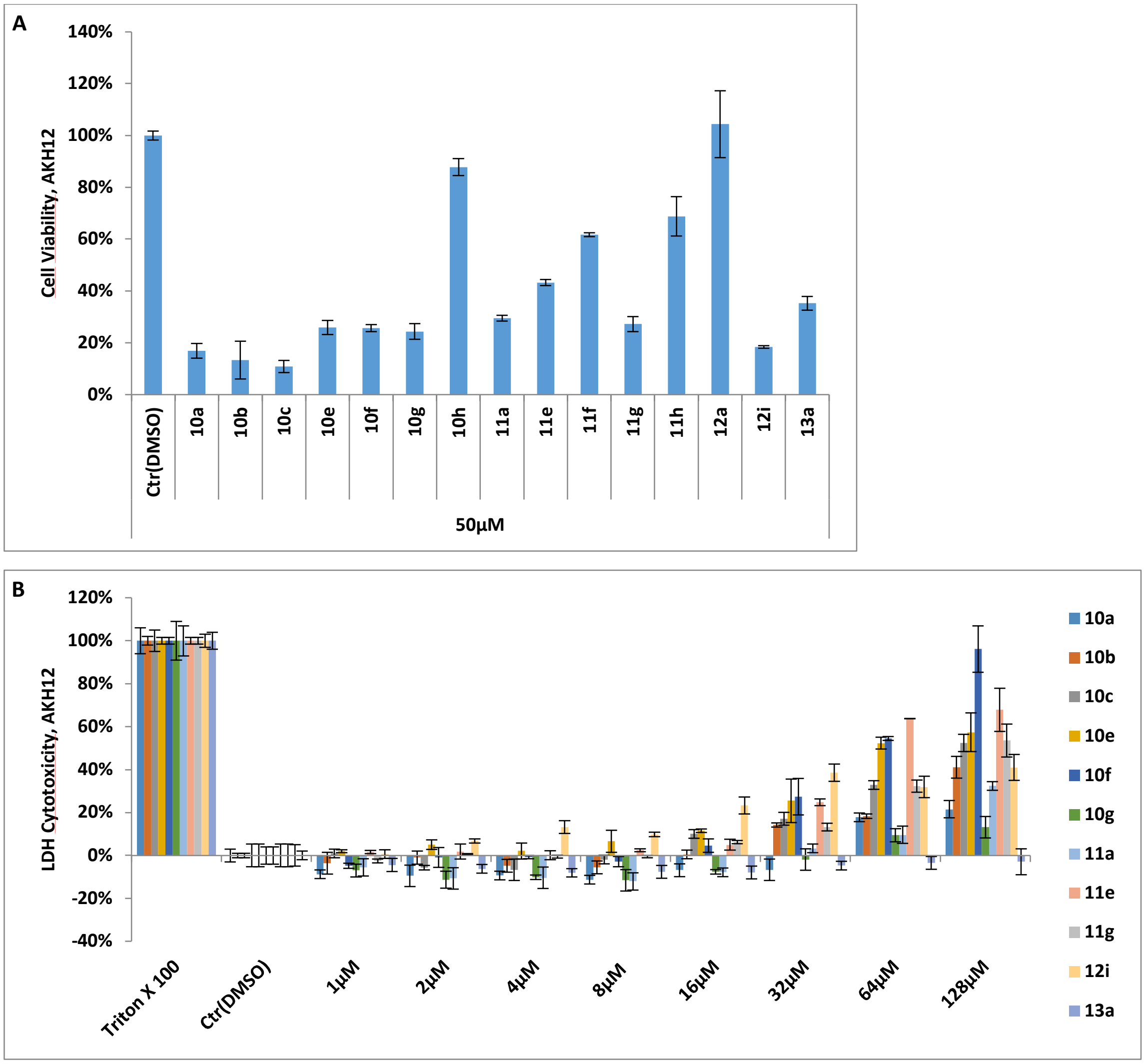

Figure S3. Viability and cytotoxicity effect of sulfonamide compounds on AKH12 cells. A) Cell viability was measured after 24 h of incubation of AKH12 cell line with 50µM of indicated sulfonamides. B) LDH release, Triton X 100 was used to induce maximum LDH leakage. All experiments were performed in triplicates; means ± SD errors are shown.

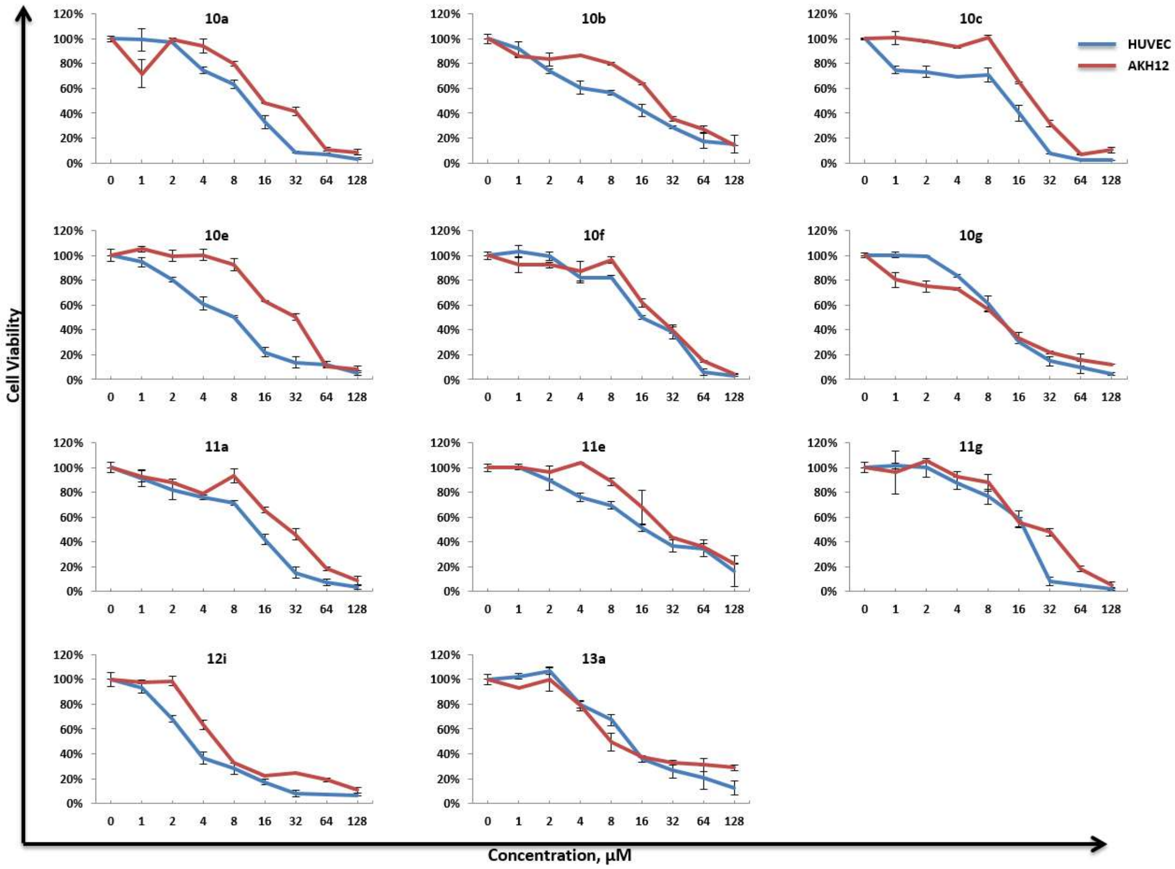

Figure S4. Impact of sulfonamide compounds on AKH12 compared to HUVEC. Cells were treated with indicated sulfonamide compounds in dose-dependent manner. No notable differences in cell viabilities were observed between both cell lines. All experiments were performed in triplicates; means ± SD errors are shown.

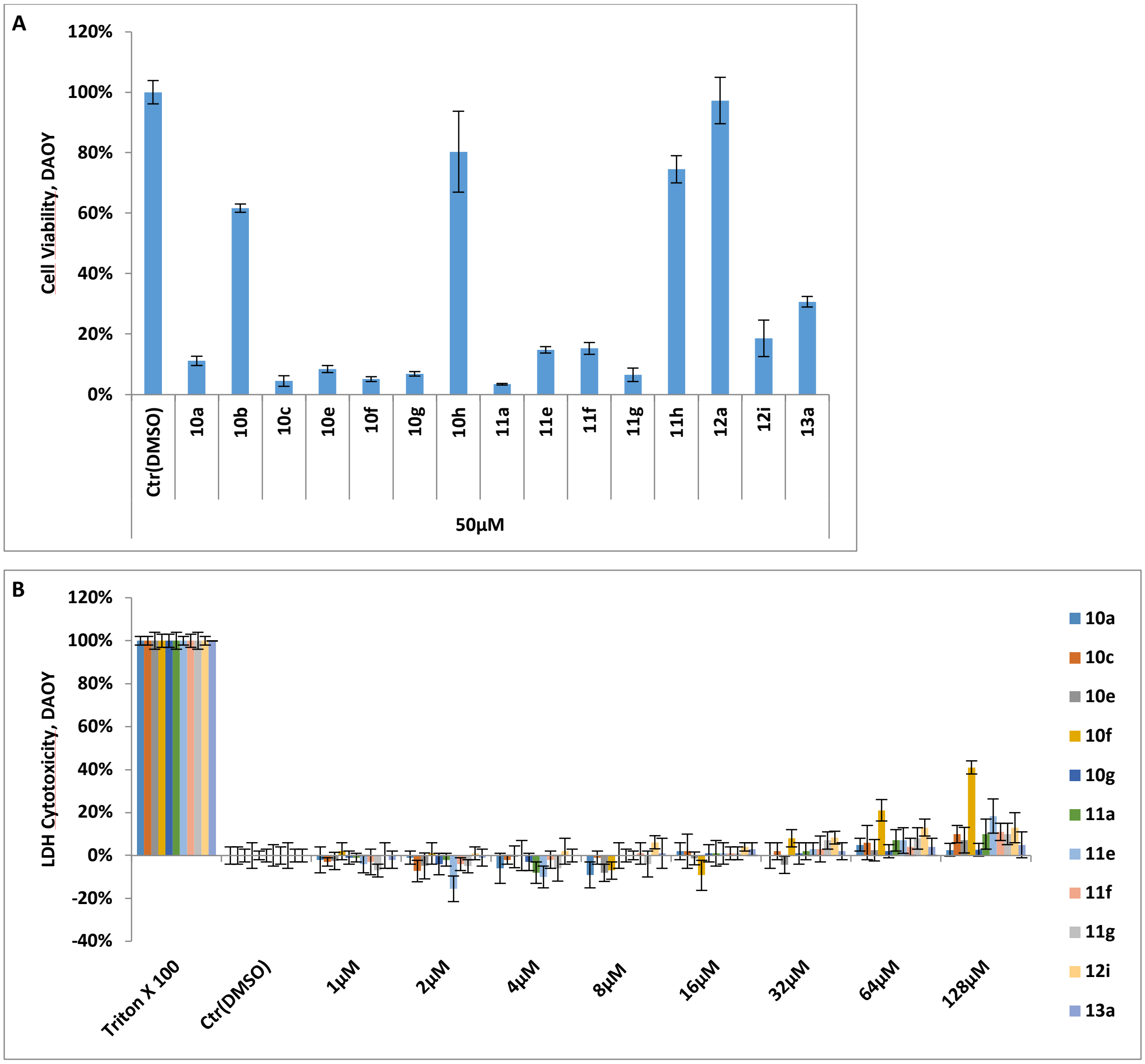

Figure S5. Influence of sulfonamide compounds treatment on DAOY cell viability and cytotoxicity. A) DAOY cells were incubated with 50µM designated sulfonamide compounds for 24 h. B) The LDH release assay for selected sulfonamide compounds. All experiments were performed in triplicates; error bars indicate ± SD.

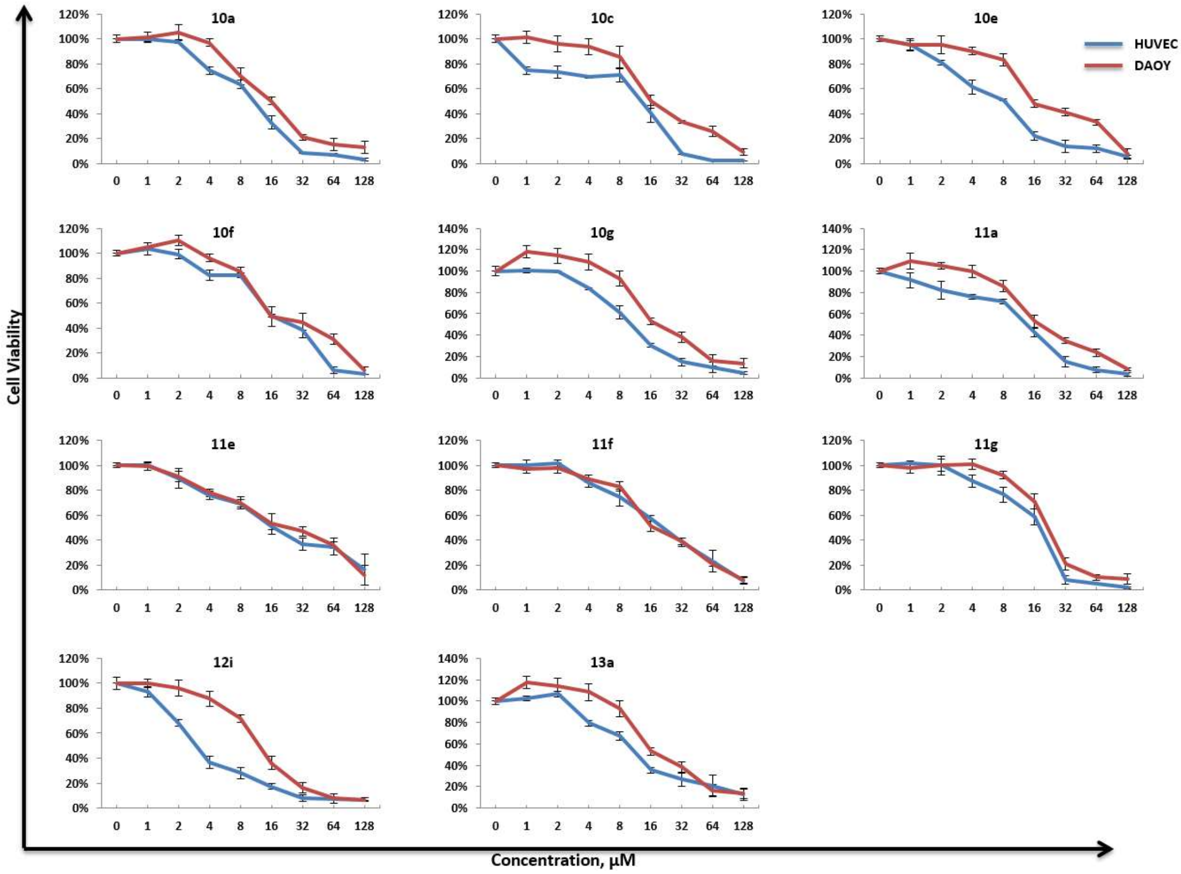

Figure S6. Sulfonamide compounds sensitivity of DAOY in contrast to HUVEC cell lines. No significant differences in cytotoxicity were observed in our experiment set up. Error bars indicate mean ±SD.

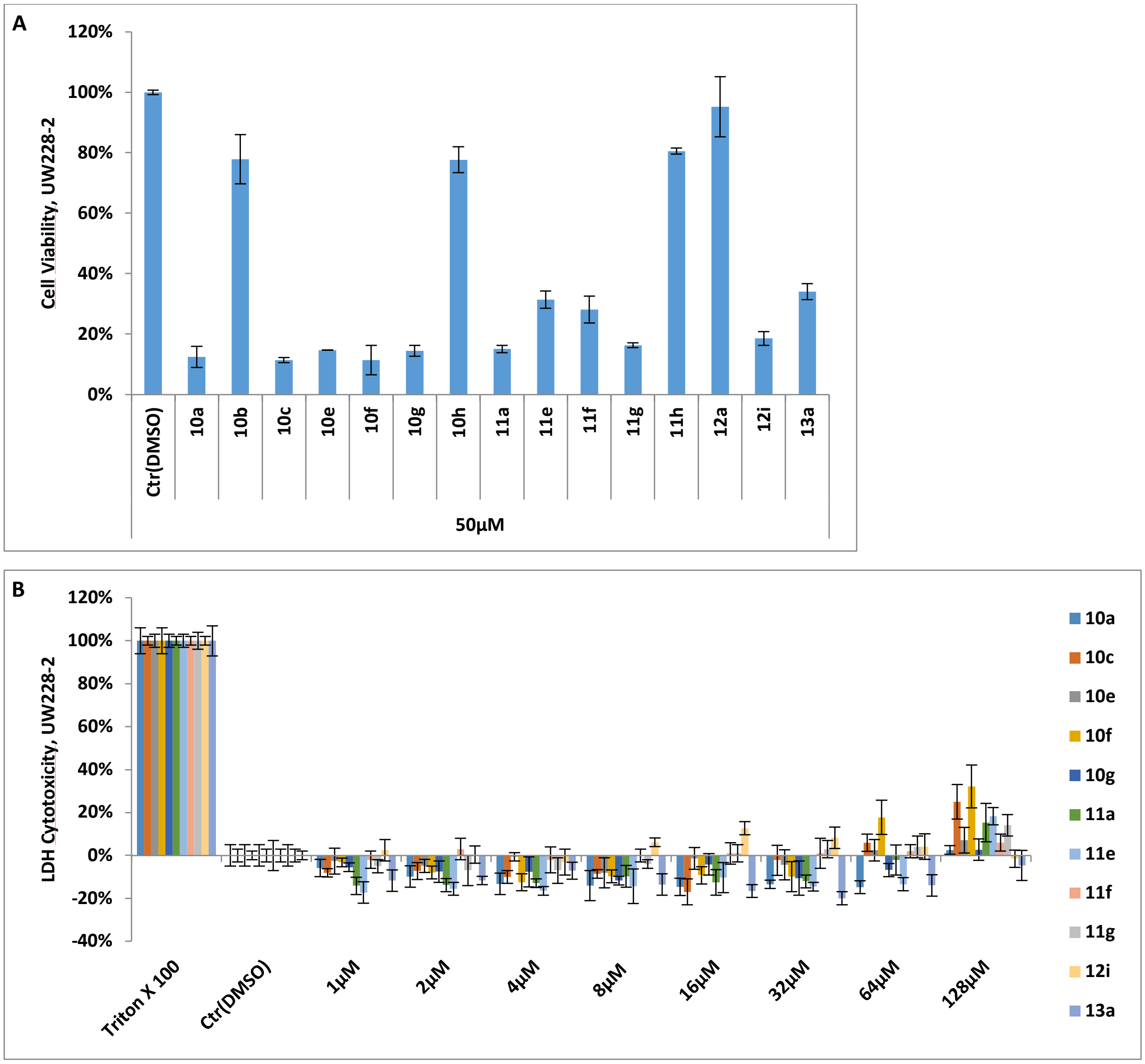

Figure S7. Effect of sulfonamides on viability of UW228-2 cell line. A) UW228-2 cells were introduced by 50µM indicated compounds for 24 h. B) Cytotoxicity was determined by LDH release of various concentrations of specified sulfonamides. All experiments were performed in triplicates; means ± SD errors are shown.

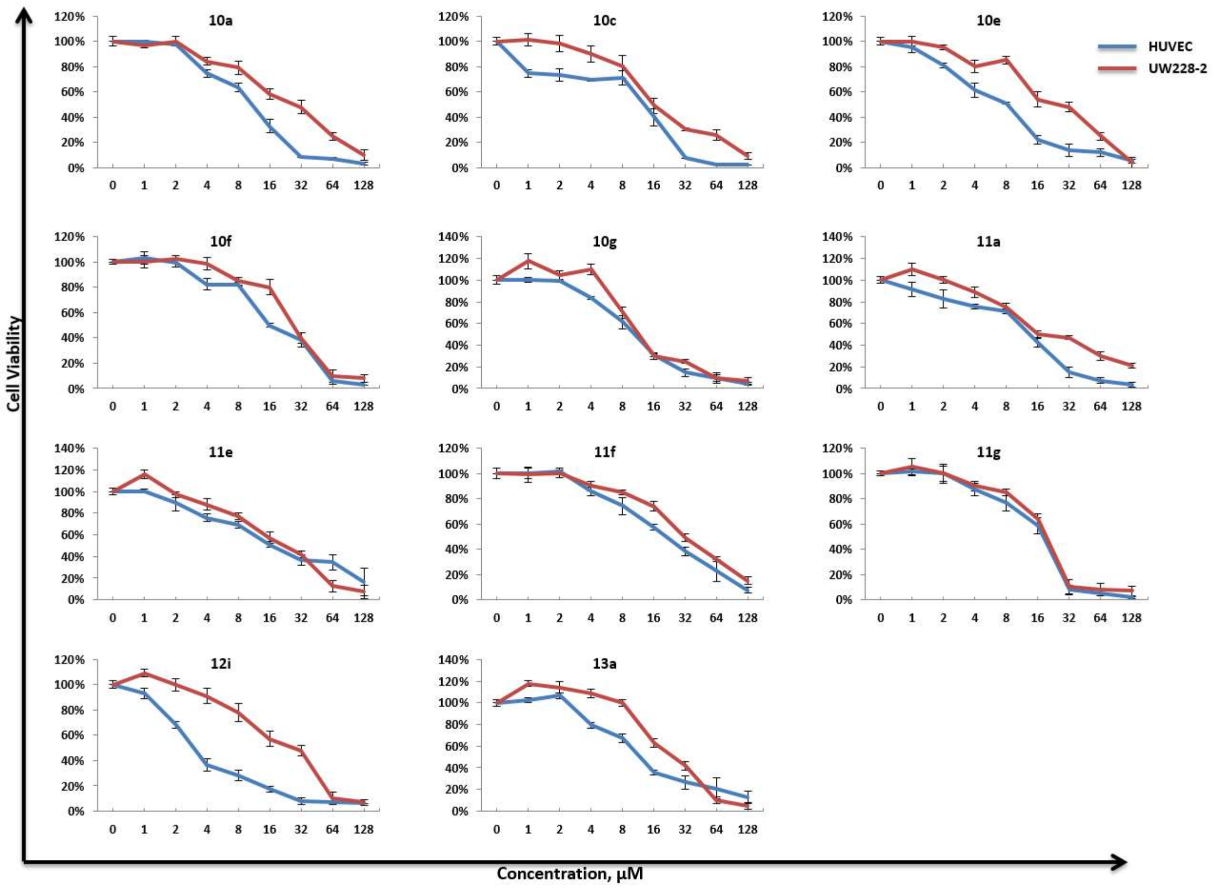

Figure S8. Impact of sulfonamides on UW228-2 in contrast to HUVEC. Cells treated with a variety of concentration of compounds or DMSO vehicle for 24 h. All experiments were performed in triplicates; errors indicate ± SD.

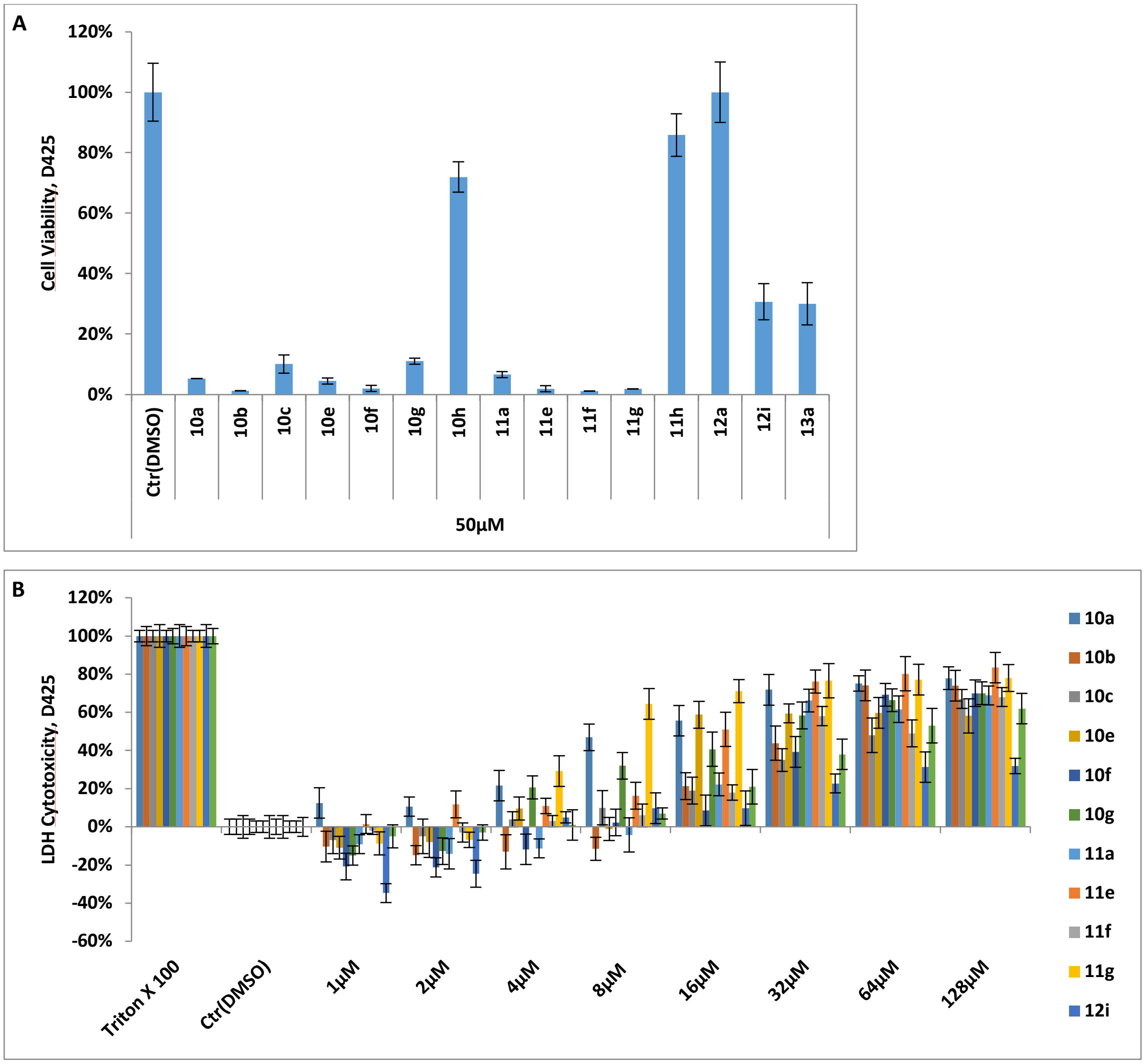

Figure S9. Sulfonamides sensitivity and cytotoxicity on D425 cells. A) D425 cells were incubated with 50µM of indicated sulfonamide compounds for 24 h. B) LDH release of sulfonamide compounds after 24 h in a dose-dependent manner. Triton X 100 used as positive control. Error bars indicate mean ±SD.

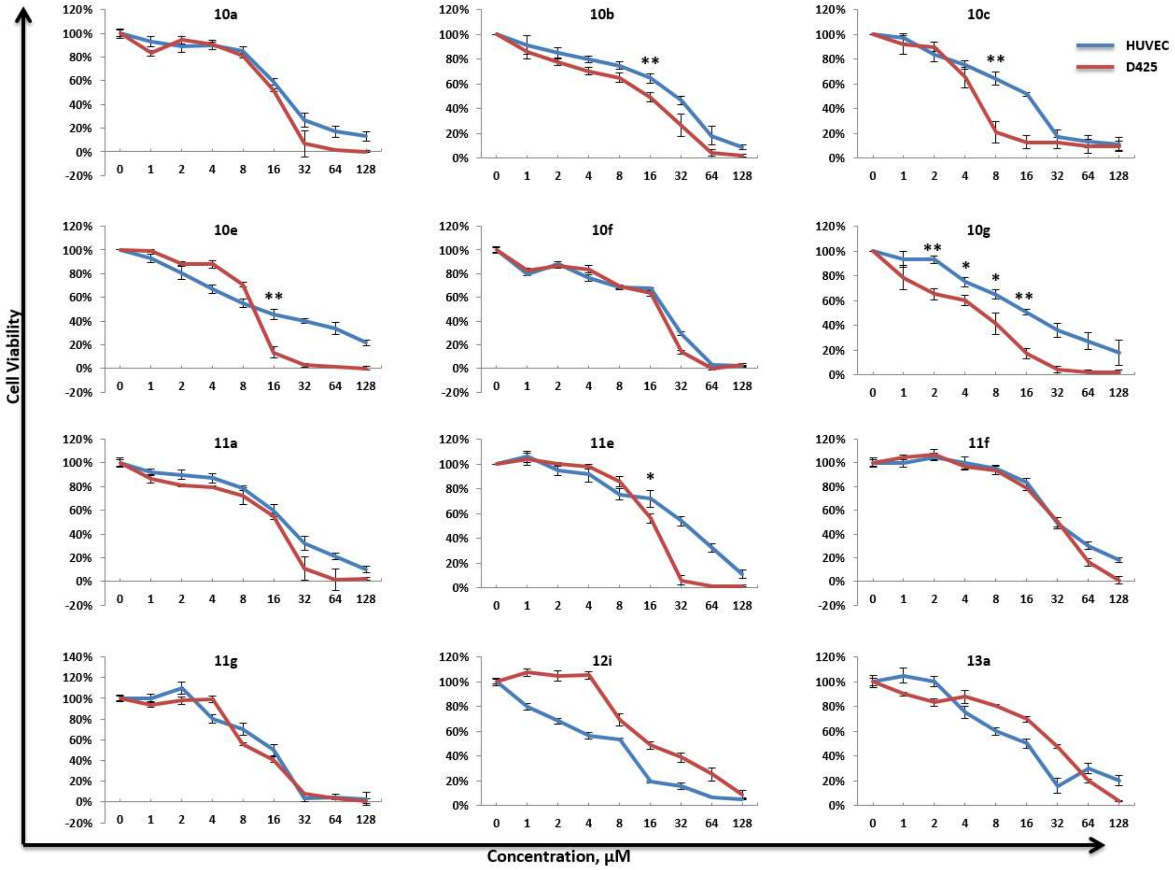

Figure S10. Effect of sulfonamides on viability of D425 in comparison with HUVEC cell lines. Grown cells were treated with mentioned compounds in 24-well plates for 24 h. Compounds 10b, 10c, 10e, 10g, and 11e resulted a significant higher viability on HUVEC cells as compared to D425 cells. Error bars indicate standard error of mean of triplicate assays. Asterisks indicate statistical significance analyzed by student's t-test (*P≤0.05, and **P≤0.01).

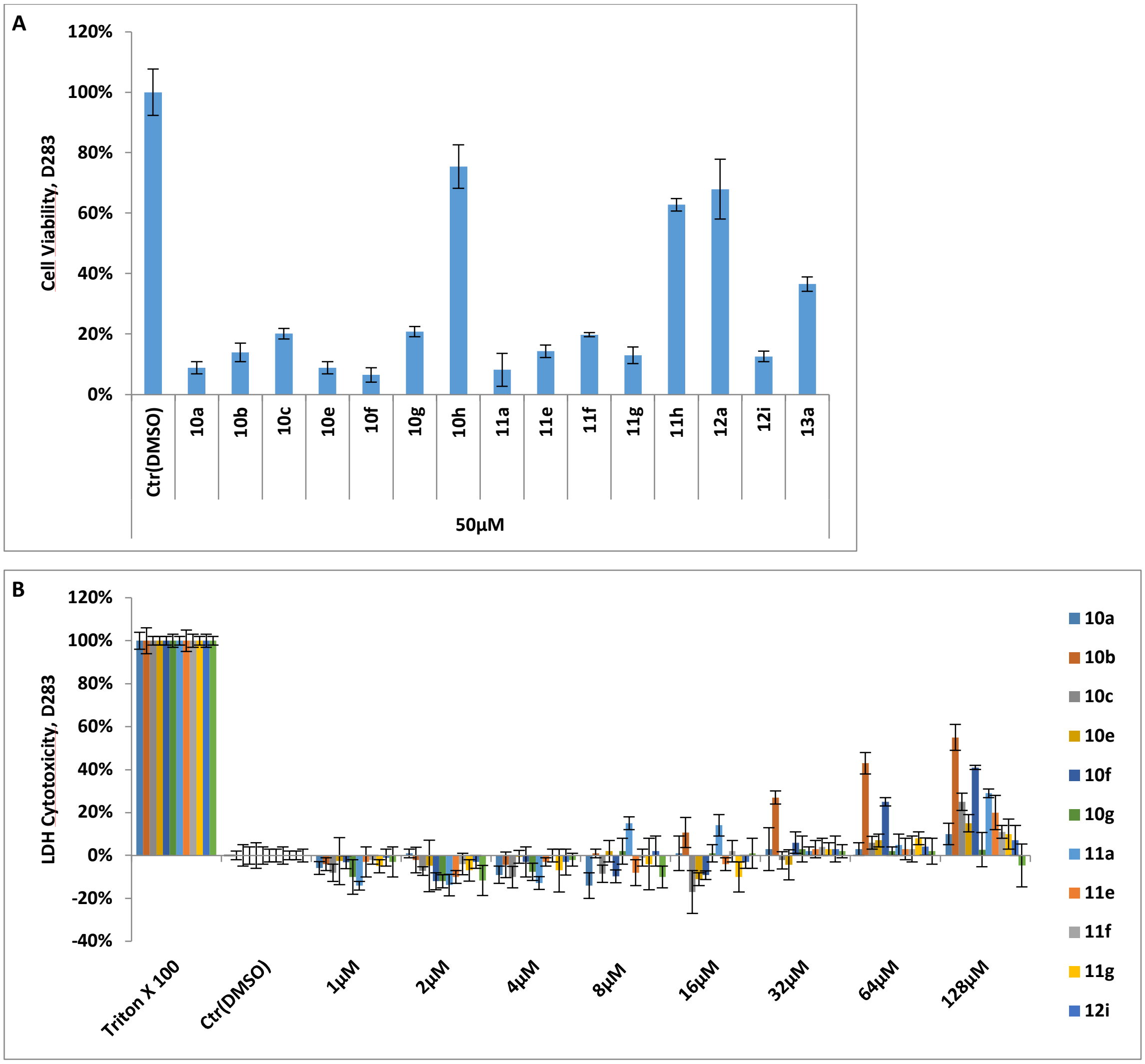

Figure S11. Influence of sulfonamide compounds treatment on D283 cell viability and cytotoxicity. A) D283 cells were incubated with 50µM designated sulfonamide compounds for 24 h. B) The LDH release assay for selected sulfonamide compounds. All experiments were performed in triplicates; errors indicate ± SD.

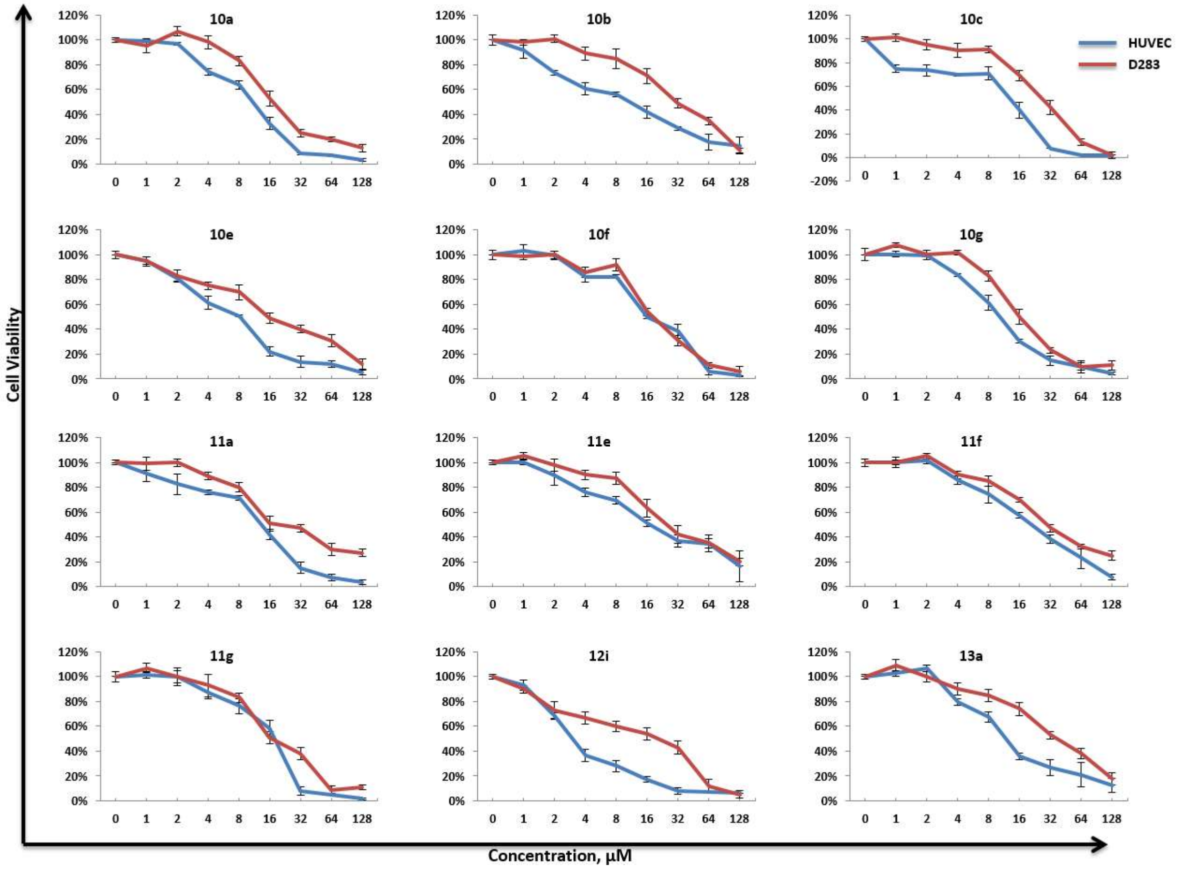

Figure S12. Efficacy of sulfonamides on cell viability of D283 compared to HEVEC cell lines. Cell lines are treated with indicated compounds in a dose-dependent manner for 24 h. No differences in cytotoxicity between the cell lines were observed. Values are the average ±SD of triplicate experiments.

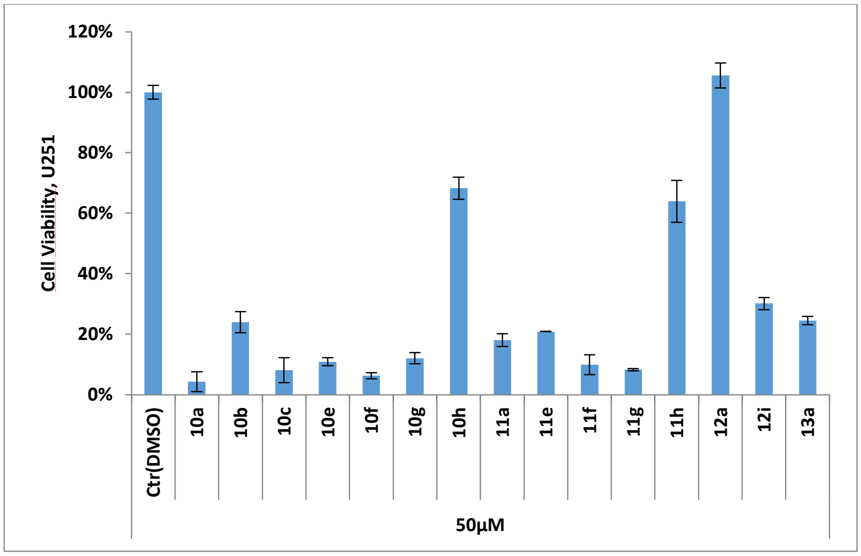

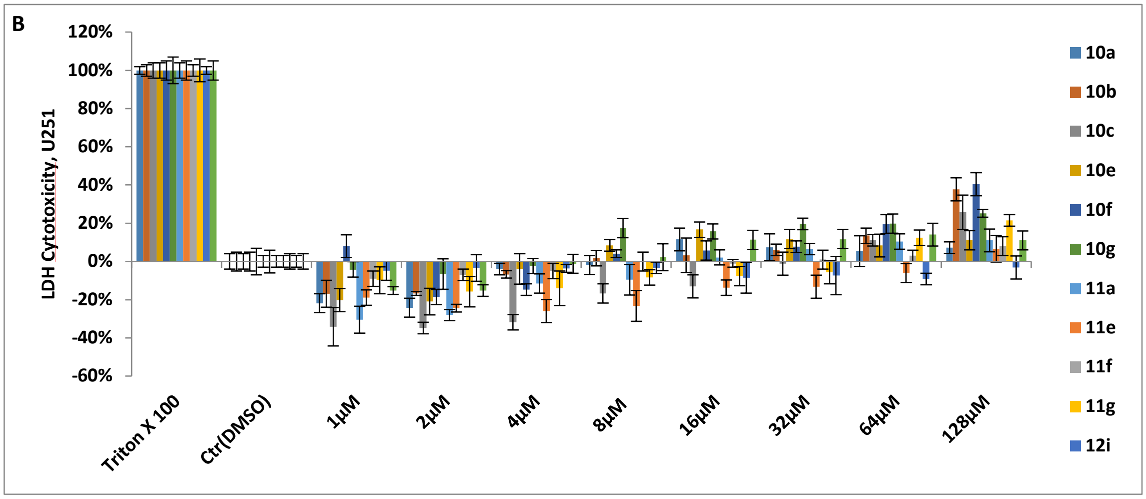

Figure S13. Sulfonamides and viability of U251 cells. A) U251 cell viability was measured after 24 h of 50μM compounds treatment. B) LDH release. Error bars indicate mean ±SD.

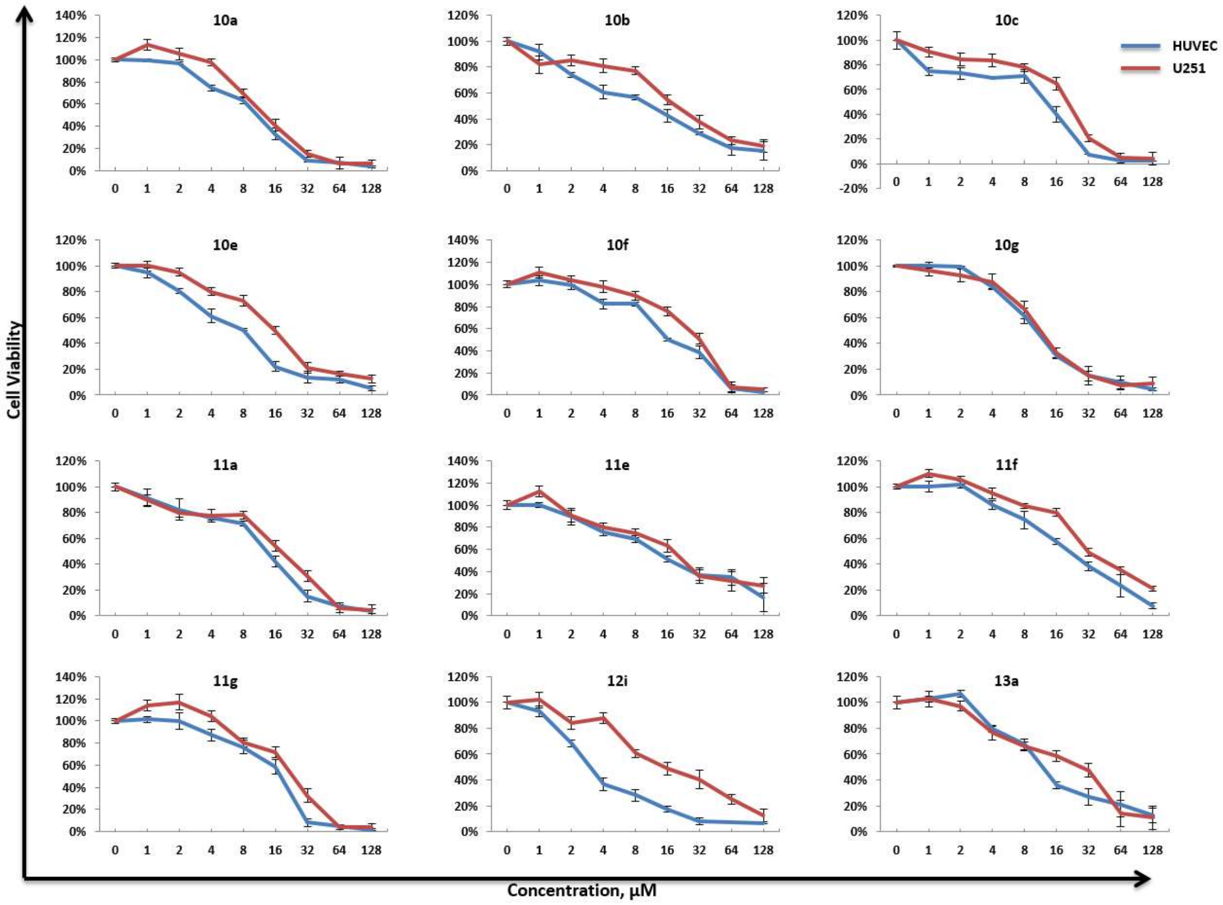

Figure S14. Efficacy of sulfonamides on cell viability of U251 compared to HEVEC cell lines. Cell lines are treated with indicated compounds in a dose-dependent manner for 24 h. No differences in cytotoxicity between the cell lines were observed. Values are the average ±SD of triplicate experiments.

X-RAY STRUCTURES AND CRYSTALOGRAPHIC DATA OF SULFONAMIDES 12a AND 12d

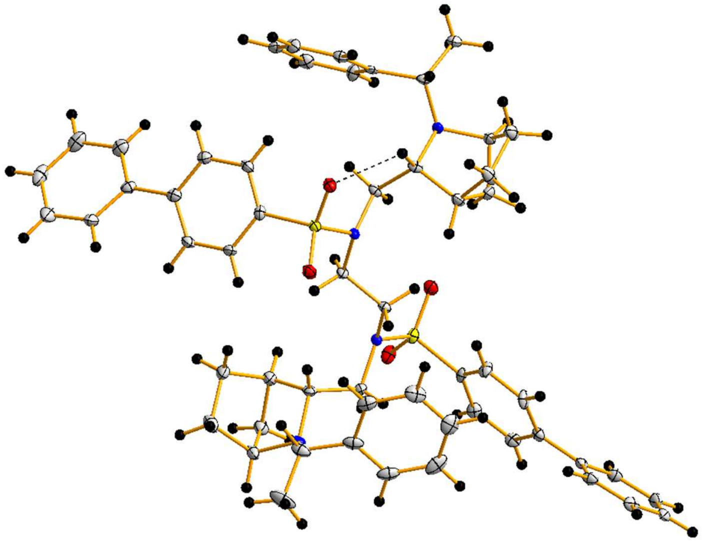

12a

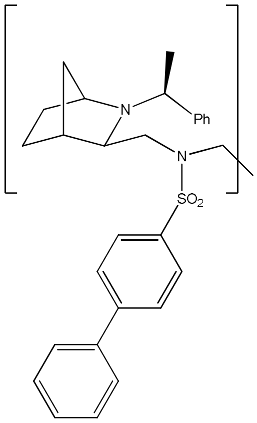

Figure S15. X-Ray structure of bis-sulfonamide 12a.

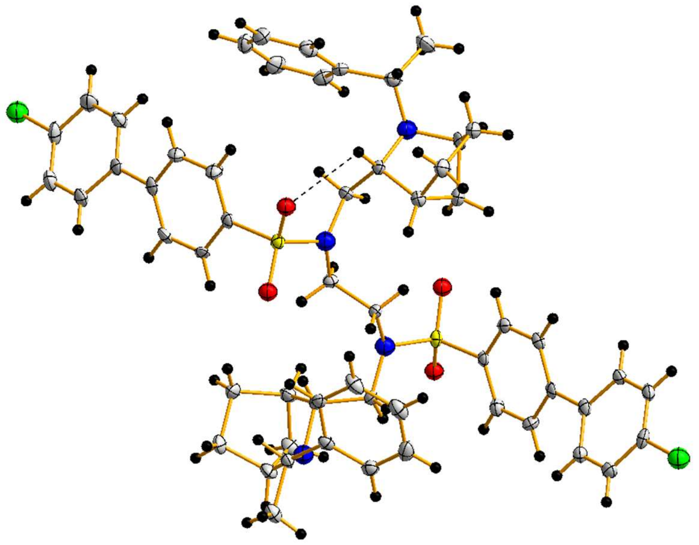

12d

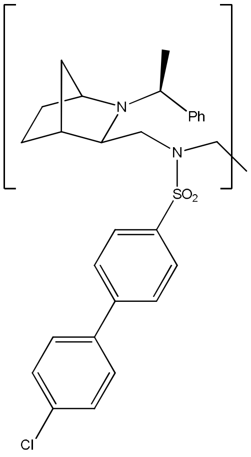

Figure S16. X-Ray structure of bis-sulfonamide 12d.

Table S1. X-Ray data for compounds **12a** and **12d**

Empirical formula	$C_{56} H_{62} N_4 O_4 S_2$ (**12a**)	$C_{56} H_{60} Cl_2 N_4 O_4 S_2$ (**12d**)
Formula weight	919.21	988.10
Temperature	80(2) K	100(2) K
Wavelength	0.71073 Å	0.71073 Å
Crystal system	Orthorhombic	Monoclinic
Space group	$P2_12_12_1$	$P2_1$
Unit cell dimensions	a = 8.704(3) Å	a = 12.914(4) Å
	b = 23.241(3) Å	b = 11.659(3) Å
	c = 23.522(2) Å	c = 17.497(3) Å
		β= 107.67(3)°.
Volume	4758.3(18) Å^3	2510.1(12) Å^3
Z	4	2
Density (calculated)	1.283 Mg/m^3	1.307 Mg/m^3
Absorption coefficient	0.164 mm^{-1}	0.264 mm^{-1}
F(000)	1960	1044.0
Crystal size	0.40 x 0.08 x 0.06 mm^3	0.30 x 0.07 x 0.05 mm^3
Theta range for data collection	2.742 to 28.904°.	2.918 to 28.654°.
Index ranges	-11<=h<=8, -16<=k<=30, -30<=l<=29	-17<=h<=11, -8<=k<=15, -15<=l<=23
Reflections collected	13797	11387
Independent reflections	10102 [R(int) = 0.0263]	7972 [R(int) = 0.0324]
Completeness to theta = 25.000°	98.7 %	99.8 %
Absorption correction	Numerical	Analytical
Max. and min. transmission	0.897 and 0.789	0.910 and 0.890
Refinement method	Full-matrix least-squares on F^2	Full-matrix least-squares on F^2
Data / restraints / parameters	10102 / 0 / 595	7972 / 1 / 613
Goodness-of-fit on F^2	0.978	1.006
Final R indices [I>2sigma(I)]	R1 = 0.0516, wR2 = 0.0972	R_1 = 0.0494, wR_2 = 0.0868
R indices (all data)	R1 = 0.0759, wR2 = 0.1070	R_1 = 0.0705, wR_2 = 0.0956
Absolute structure parameter	0.01(5)	0.03(5)
Extinction coefficient	n/a	n/a
Largest diff. peak and hole	0.257 and -0.362 e.Å^{-3}	0.395 and -0.441 e.Å^{-3}

SPECTRAL DATA

4-Phenyl-*N*-((2-((*S*)-1-phenylethyl)-(1*S*,3*R*,4*R*)-2-azabicyclo[2.2.1]heptane-3-yl) benzenesulfonamide (10a)

White solid. Yield 0.33 g (75%). Mp 110-111 °C. $[\alpha]_D^{20} = -39.4$ (*c* 0.69, CH_2Cl_2). ^{1}H NMR (600 MHz, $CDCl_3$): δ 1.23-1.25 (d, *J* =9.8 Hz, 2H), 1.29-1.30 (d, *J* = 6.5 Hz, 3H), 1.35-1.42 (m, 1H), 1.59-1.66 (m, 2H), 1.85-1.97 (m, 3H), 2.18-2.19 (d, *J* = 3.9 Hz, 1H), 2.23-2.26 (m, 1H), 3.41-3.45 (q, *J* = 6.5 Hz, 1H), 3.61 (s, 1H), 4.32 (s, 1H), 7.00-7.04 (m, 1H), 7.06-7.08 (m, 2H), 7.19-7.21 (m, 2H), 7.43-7.50 (m, 1H), 7.52-7.55 (m, 2H), 7.61-7.68 (m, 6H) ppm.

Solvents peaks: ethyl acetate 1.27 (t) overlapped peak, 2.07 (s), 4.13-4.17 (q), chloroform 7.29 (s) ppm.

^{13}C NMR ($CDCl_3$, 125 MHz): δ 22.2, 22.5, 29.4, 35.7, 41.9, 47.0, 58.7, 60.7, 67.6, 127.3, 127.3, 127.5, 127.5, 128.2, 128.4, 129.0, 129.1, 138.2, 139.6, 145.1, 145.8 ppm.

Solvents peaks: chloroform-*d* 77.1 ppm (t).

IR (KBr): 3435, 3282, 2967, 2871, 1329, 1162, 763, 700, 672, 591 cm^{-1}.

HRMS (ESI+, *m/z*): calcd for $[C_{27}H_{31}N_2O_2S]^+$ ($[M+H]^+$) 447.2106; found 447.2107.

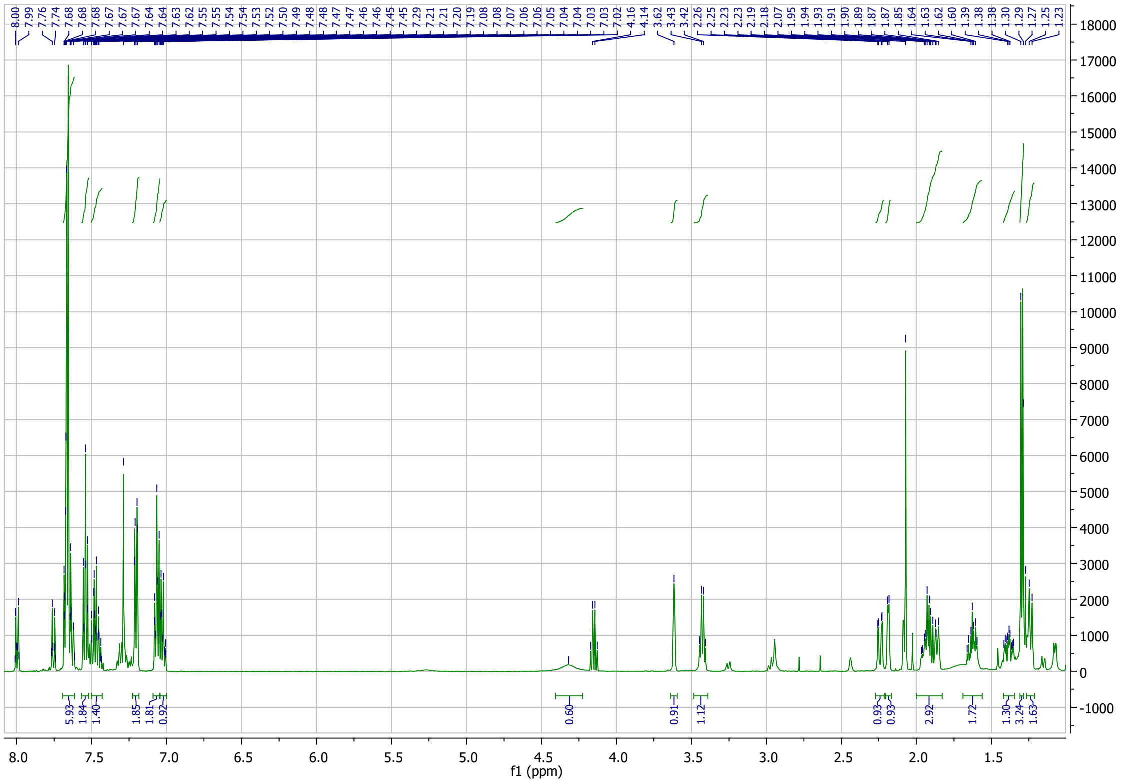

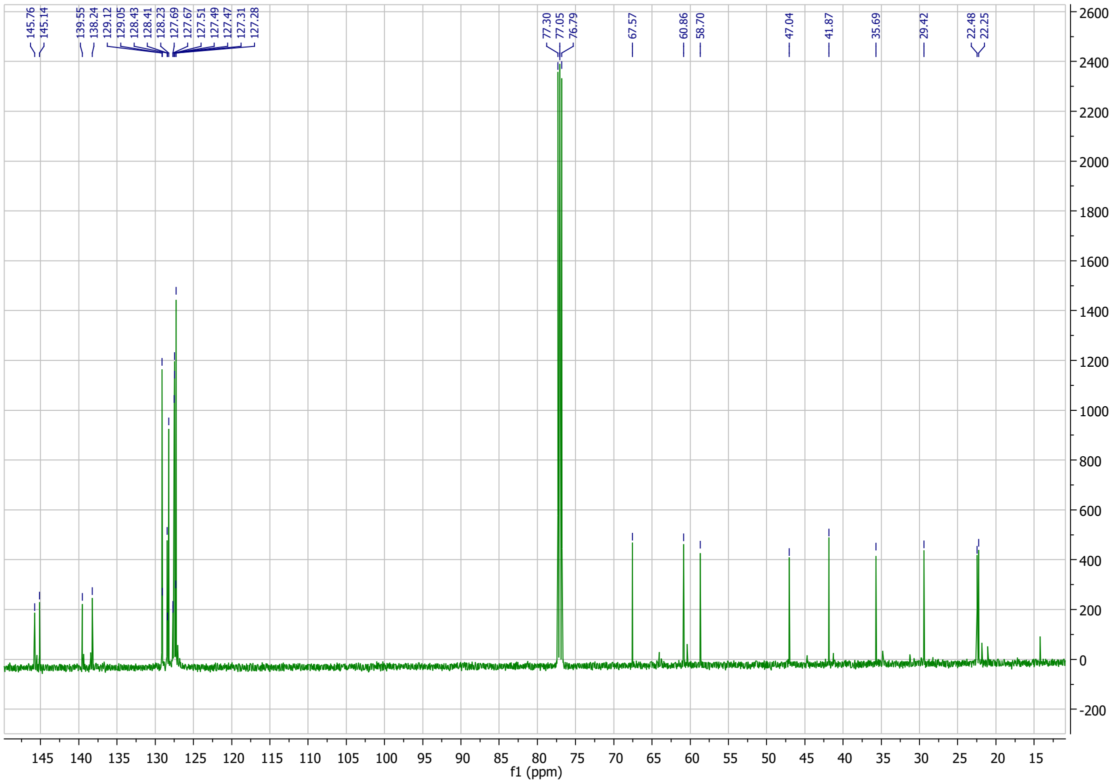

4-(4-Tolyl)-*N*-((2-((*S*)-1-phenylethyl)-(1*S*,3*R*,4*R*)-2-azabicyclo[2.2.1]heptane-3-yl)benzenesulfonamide (10b)

White solid. Yield 0.15 g (30%). Mp 181-182 °C. $[\alpha]_D^{20}$ = +52.3 (*c* 0.50, CH_2Cl_2). ^{1}H NMR (600 MHz, $CDCl_3$): δ 1.19-1.26 (m, 5H), 1.52-1.63 (m, 2H). 1.82-1.95 (m, 2H), 2.02-2.04 (m, 1H), 2.14-2.22 (m, 3H), 2.42 (s, 3H), 3.37 (q, *J* = 6.4 Hz, 1H), 3.57 (s, 1H), 4.19 (s, 1H, NH), 6.95-7.03 (m, 3H, ArH), 7.14-7.16 (m, 2H, ArH), 7.30-7.32 (d, *J* = 8.0 Hz, 2H, ArH), 7.51-7.54 (d, *J* = 8.0 Hz, 2H, ArH), 7.57-7.63 (m, 4H, ArH) ppm.

Solvents peaks: chloroform 7.25 (s) ppm.

^{13}C NMR ($CDCl_3$, 125 MHz): δ 21.3, 22.3, 22.5, 29.5, 35.8, 42.0, 47.1, 58.7, 60.9, 67.6, 127.2, 127.3, 127.4, 127.5, 127.6, 128.3, 129.9, 136.7, 137.9, 138.5, 145.2, 145.8 ppm.

Solvents peaks: chloroform-*d* 77.1 ppm (t).

IR (KBr): 3436, 3346, 2964, 1540, 1332, 1164, 811, 633, 561 cm^{-1}.

HRMS (ESI+, *m/z*): calcd for $[C_{28}H_{33}N_2O_2S]^+$ ($[M+H]^+$) 461.2263; found 461.2271.

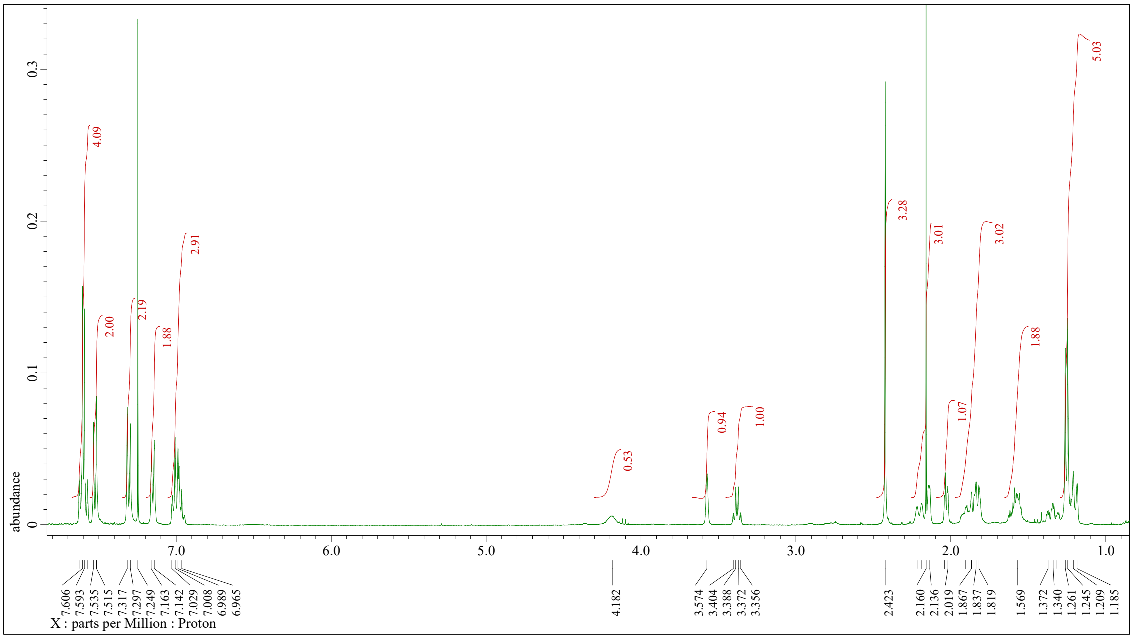

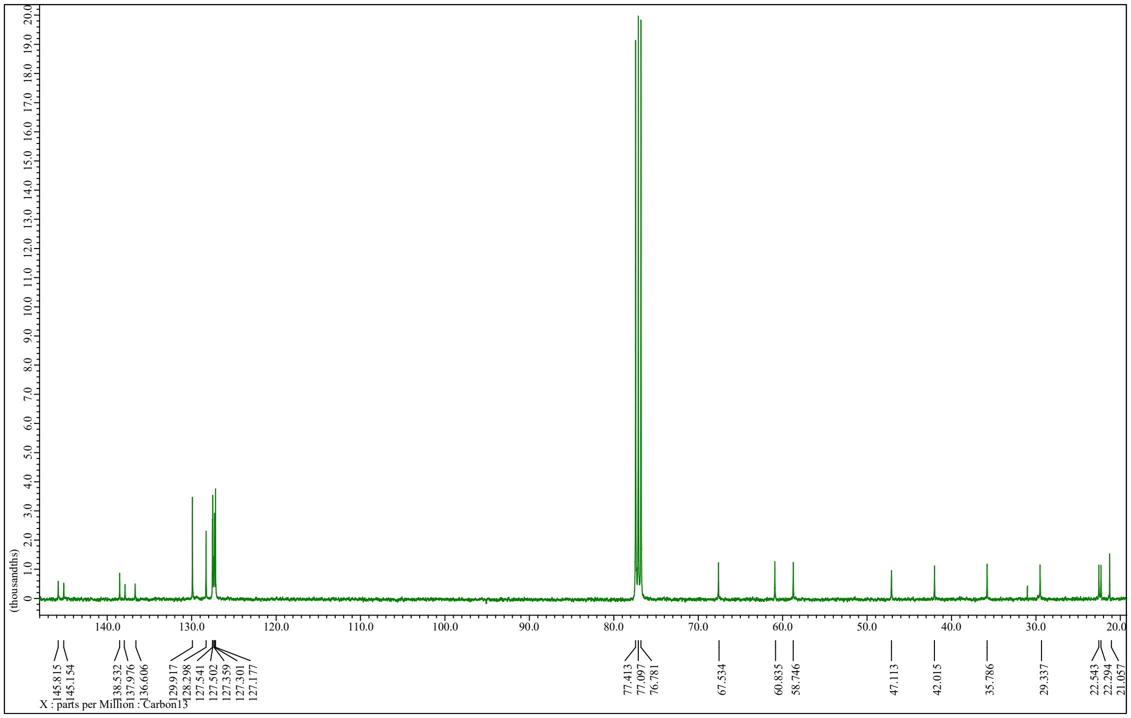

4-(4-Fluorophenyl)-*N*-((2-((*S*)-1-phenylethyl)-(1*S*,3*R*,4*R*)-2-azabicyclo[2.2.1]heptane-3-yl)benzenesulfonamide (10c)

White solid. Yield 0.32 g (69%). Mp 126-127 °C. $[\alpha]_D^{20}$ = +34.0 (*c* 0.50, CH_2Cl_2). ^{1}H NMR (600 MHz, $CDCl_3$): δ 1.19-1.26 (m, 5H), 1.52-1.63 (m, 2H). 1.82-1.95 (m, 2H), 2.02-2.04 (m, 1H), 2.14-2.22 (m, 3H), 2.42 (s, 3H), 3.37 (q, *J* = 6.4 Hz, 1H), 3.57 (s, 1H), 4.19 (s, 1H, NH),

6.95-7.03 (m, 3H, ArH), 7.14-7.16 (m, 2H, ArH), 7.30-7.32 (d, *J* = 8.0 Hz, 2H, ArH), 7.51-7.54 (d, *J* = 8.0 Hz, 2H, ArH), 7.57-7.63 (m, 4H, ArH) ppm.

Solvents peaks: chloroform 7.25 (s) ppm.

^{13}C NMR ($CDCl_3$, 125 MHz): δ 22.3, 22.6, 29.5, 35.8, 42.0, 47.1, 58.8, 60.9, 67.6, 116.0, 116.2 (d, *J* = 26 Hz), 116.1, 116.3 (d, *J* = 27.5 Hz), 127.3, 127.4, 127.5, 127.6 (d, *J* = 3.5 Hz), 128.3, 129.0, 129.1 (d, *J* = 8.4 Hz), 135.7, 138.3, 144.2, 145.9, 161.9, 164.4 (d, *J* = 309 Hz).

Solvents peaks: chloroform-*d* 77.1 ppm (t).

IR (KBr): 3369, 2969, 1601, 1490, 1336, 1168, 1158, 1048, 825, 700, 632 cm^{-1}.

HRMS (ESI+, *m/z*): calcd for $[C_{28}H_{33}N_2O_2S]^+$ ($[M+H]^+$) 465.2000; found 465.1984.

,

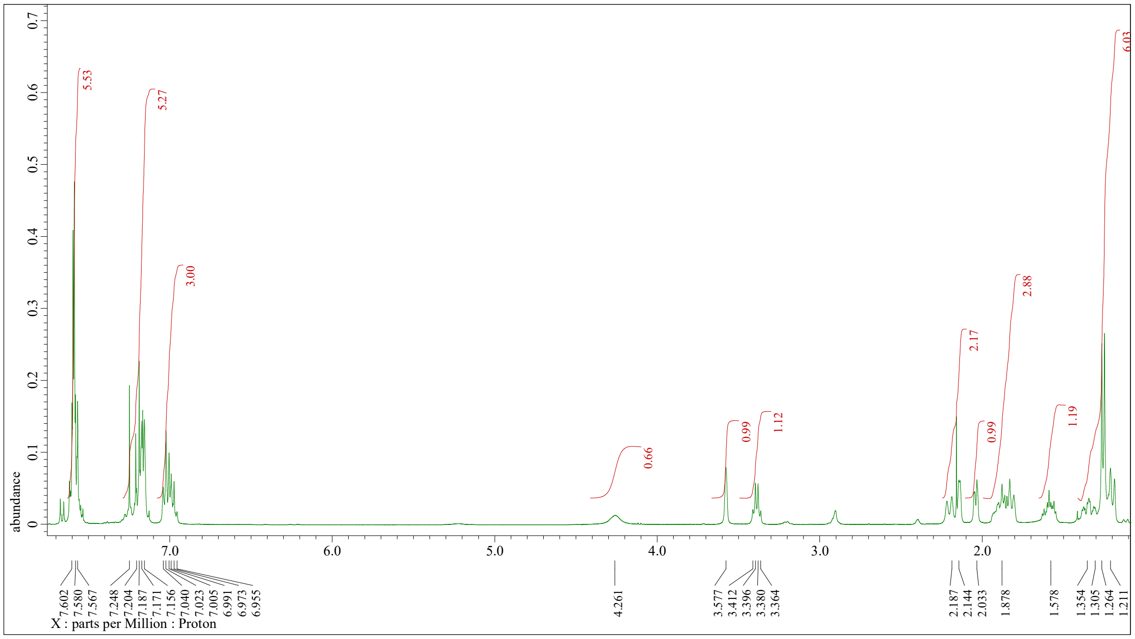

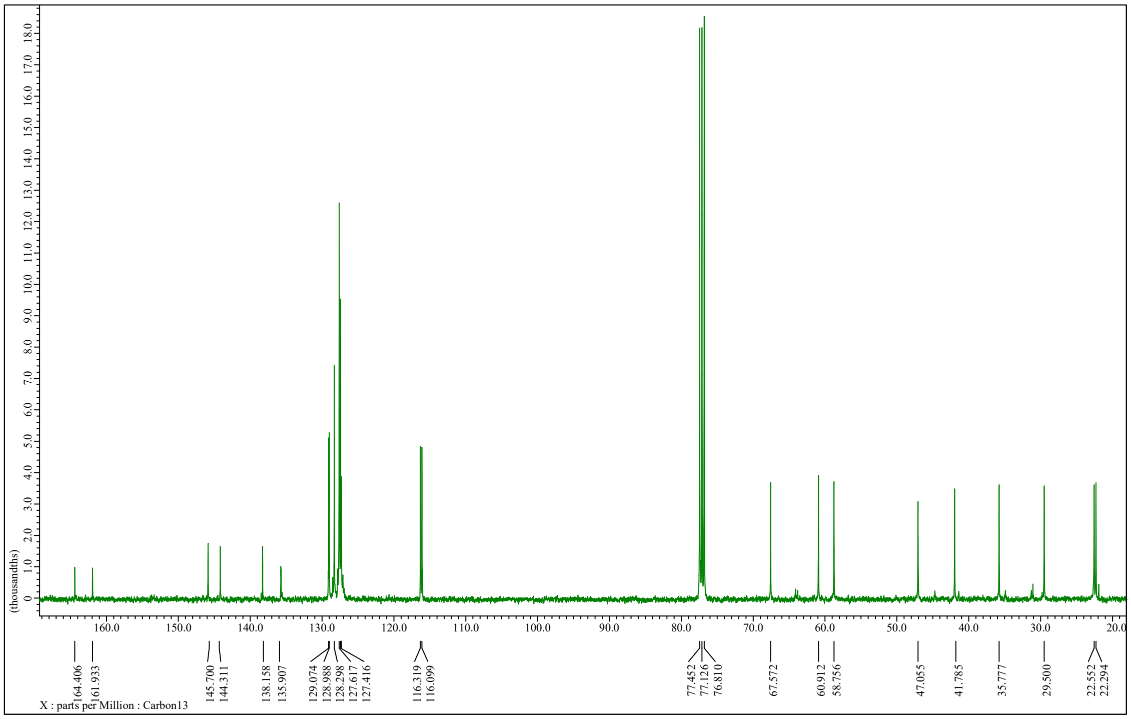

4-(4-Metoxyphenyl)-*N*-((2-((*S*)-1-phenylethyl)-(1*S*,3*R*,4*R*)-2-azabicyclo[2.2.1]heptane-3-yl)benzenesulfonamide (10e)

White solid. Yield 0.33 g (70%). Mp 155-157 °C. $[\alpha]_D^{20}$ = +50.0 (*c* 0.50, CH_2Cl_2). ^1H NMR (600 MHz, $CDCl_3$): δ 1.23-1.24 (d, *J* =9.8 Hz, 2H), 1.29-1.30 (d, *J* = 6.5 Hz, 3H), 1.35-1.41 (m, 1H), 1.59-1.66 (m, 3H), 1.85-1.77 (m, 3H), 2.06-2.08 (dd, J_1 = 6.8 Hz, J_2 = 2.1 Hz, 1H), 2.17-2.18 (d, *J* = 3.9 Hz, 1H), 2.22-2.25 (m, 1H), 3.40-3.44 (q, *J* = 6.5 Hz, 1H), 3.61 (s, 1H), 3.91 (s, 3H), 4.22 (s, 1H), 7.00-7.07 (m, 5H, ArH), 7.19-7.20 (m, 2H, ArH), 7.60-7.62 (m, 6H, ArH) ppm.

Solvents peaks: chloroform 7.29 (s) ppm.

^{13}C NMR ($CDCl_3$, 125 MHz): δ 22.2, 22.5, 29.4, 35.7, 41.9, 47.1, 55.4, 58.7, 60.9, 67.6, 114.6, 126.9, 127.3, 127.5, 127.5, 128.2, 128.4, 131.9, 137.5, 144.7, 145.8, 160.0 ppm.

Solvents peaks: chloroform-*d* 77.1 ppm (t).

IR (KBr): 3435, 3261, 2968, 2871, 1608, 1455, 1325, 1250, 1156, 1097, 822, 626 cm^{-1}.

HRMS (ESI+, *m/z*): calcd for $[C_{28}H_{33}N_2O_3S]^+$ ($[M+H]^+$) 477.2212; found 477.2226.

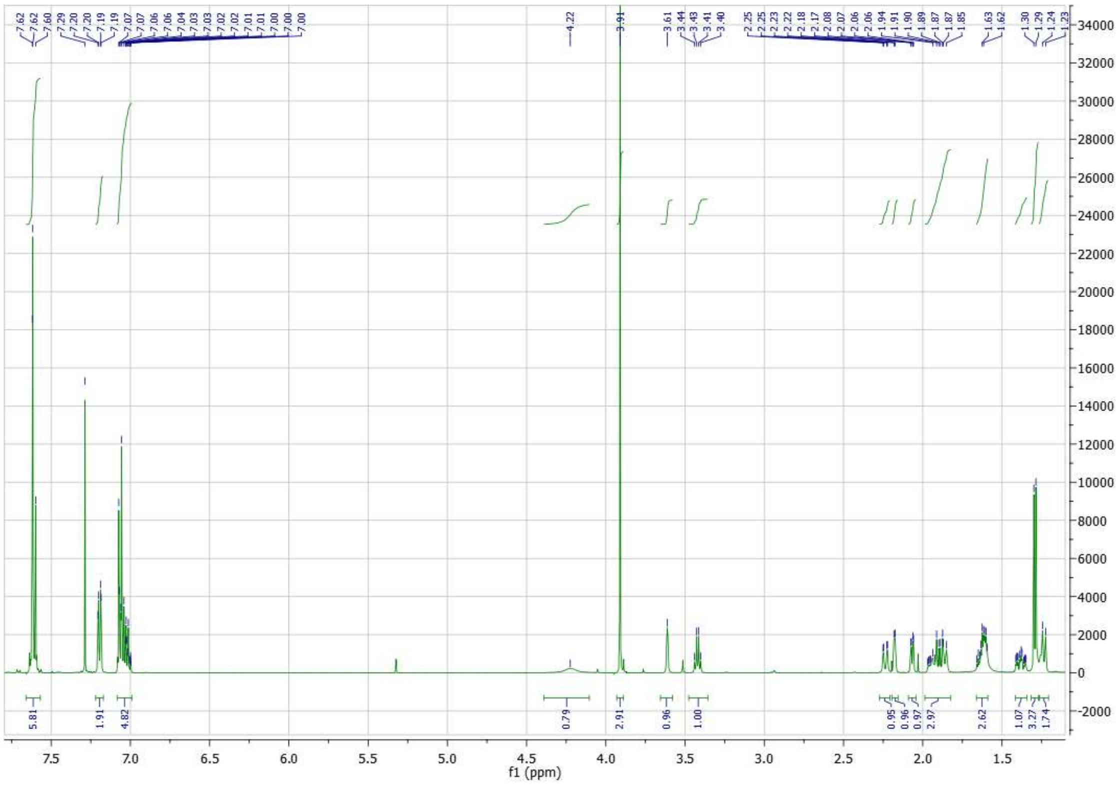

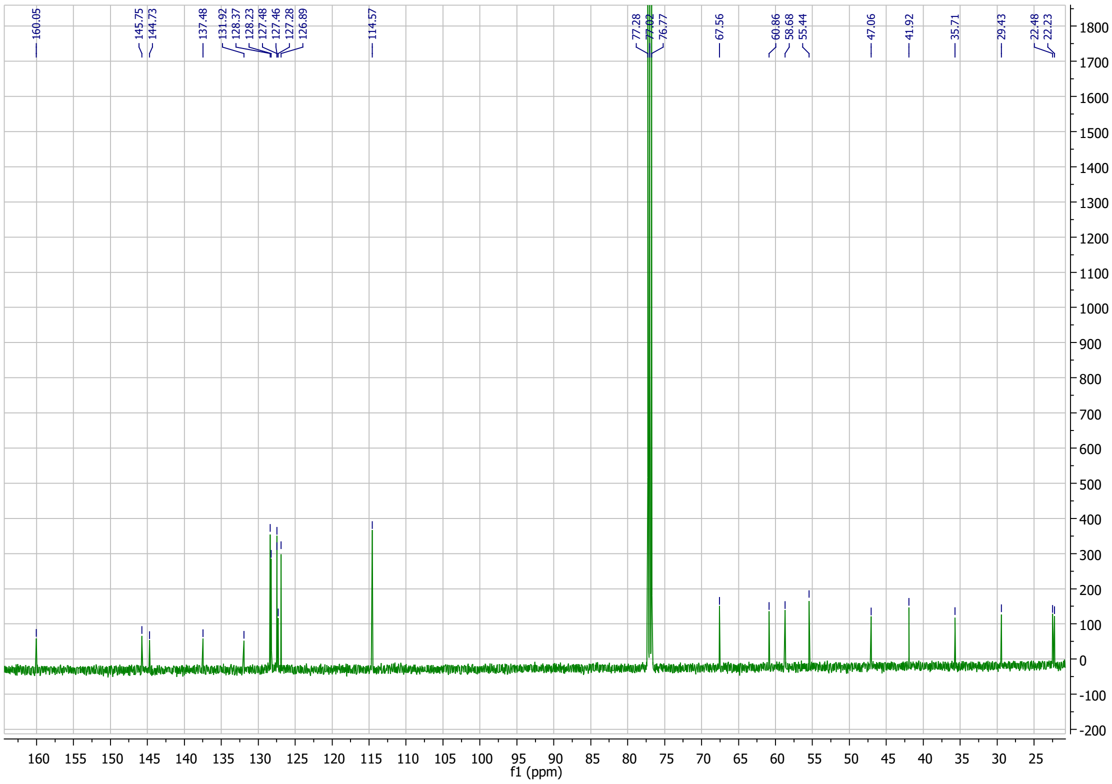

4-Trifluoromethyl-*N*-((2-((*S*)-1-phenylethyl)-(1*S*,3*R*,4*R*)-2-azabicyclo[2.2.1]heptane-3-yl) benzenesulfonamide (10f)

Yellow solid. Yield 0.35 g (81%). Mp 108-109 °C. $[\alpha]_D^{20}$ = +14.5 (*c* 0.51, CH_2Cl_2). ^{1}H NMR (600 MHz, $CDCl_3$): δ 1.23-1.25 (d, 2H), 1.29-1.30 (d, *J* = 6.5 Hz, 3H), 1.36-1.45 (m, 1H), 1.60-1.66 (m, 1H), 1.80-1.85 (m, 2H), 1.91-1.97 (m, 1H), 2.06-2.08 (dd, J_1 = 6.8 Hz, J_2 = 1.7 Hz, 1H), 2.16-2.17 (m, 1H), 2.23-2.26 (m, 1H), 3.40-3.44 (q, *J* = 6.5 Hz, 1H), 3.62 (s, 1H), 4.34 (s, 1H), 7.02-7.03 (m, 3H, ArH), 7.17-7.19 (m, 2H, ArH), 7.23-7.33 (m, 1H, ArH), 7.69-7.74 (m, 3H, ArH) ppm.

Solvents peak: chloroform 7.29 (s) ppm- overlapped peak.

^{13}C NMR ($CDCl_3$, 125 MHz): δ 22.2, 22.4, 29.5, 35.7, 42.0, 46.8, 58.7, 60.8, 67.4, 122.3, 124.4, 126.0, 126.0, 126.0, 126.1 (q, *J* = 3.6 Hz), 126.2, 126.3 (q, *J* = 3.6 Hz), 126.9, 127.3, 127.4, 127.5, 127.6, 128.2, 128.4 (q, *J* = 33.9 Hz), 133.8, 134.1 (d, *J* = 32.9 Hz), 143.1, 145.8 ppm.

Solvents peaks: chloroform-*d* 77.1 ppm (t).

IR (KBr): 3350, 2977, 1405, 1323, 1165, 1062, 711, 604, 430 cm^{-1}.

HRMS (ESI+, *m/z*): calcd for $[C_{22}H_{26}F_3N_2O_2S]^+$ ($[M + H]^+$) 439.1667; found 439.1670.

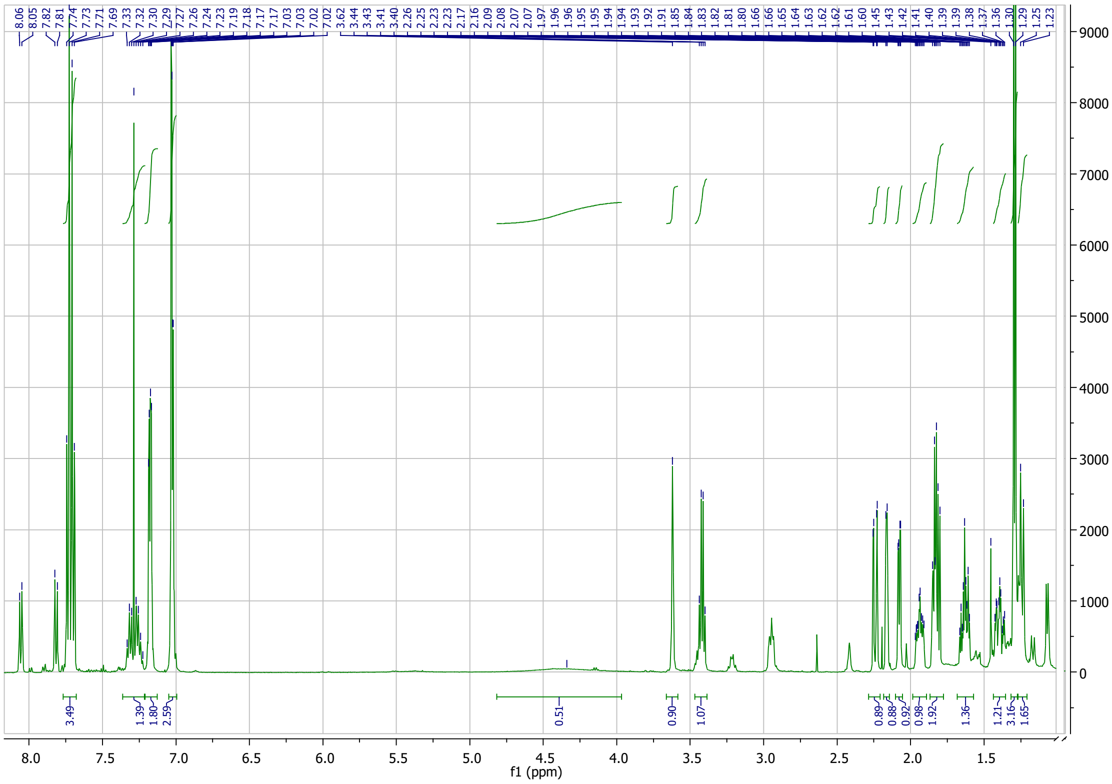

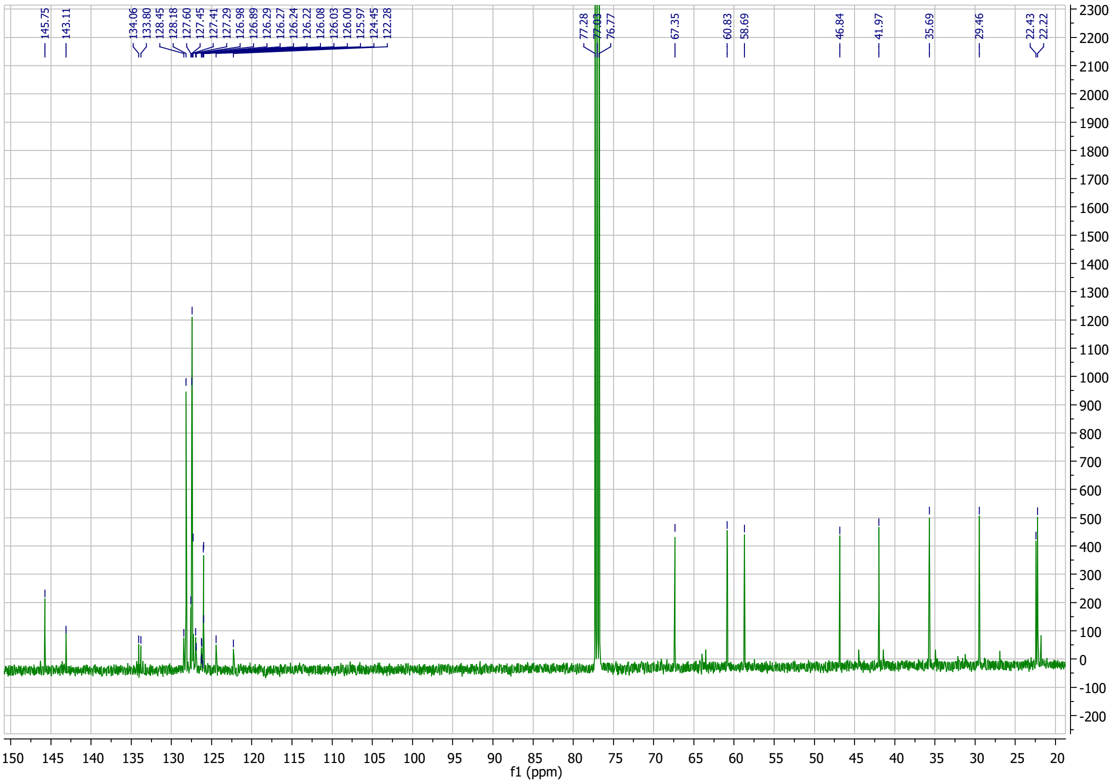

3,5-Bis(trifluoromethyl)-*N*-((2-((*S*)-1-phenylethyl)-(1*S*,3*R*,4*R*)-2-azabicyclo[2.2.1]heptane-3-yl)benzenesulfonamide (10g)

Yellow solid. Yield 0.44 g (86%). Mp 82-83 °C. $[\alpha]_D^{20}$ = +13.6 (*c* 0.76, CH_2Cl_2). ^{1}H NMR (600 MHz, $CDCl_3$): δ 1.25-1.27 (d, *J* = 9.8 Hz, 2H), 1.31-1.32 (d, J = 6.5 Hz, 3H), 1.38-1.44 (m, 1H), 1.61-1.67 (m, 1H), 1.80-1.84 (m , 2H), 1.91-1.97 (m, 1H), 2.08-2.09 (m, 1H), 2.14-2.15 (d, *J* = 3.9 Hz), 2.29-2.32 (dd, J_1 = 12.1 Hz, J_2 = 1.7 Hz, 1H), 3.40-3.44 (q, *J* = 6.5 Hz, 1H), 3.64 (s, 1H), 6.97-7.03 (m, 3H, ArH), 7.17-7.19 (m, 2H, ArH), 8.06-8.09 (m, 3H, ArH) ppm.

Solvents peaks: ethyl acetate 1.29 (t)- overlapped peak, 2.07 (s), 4.13-4.17 (q), chloroform 7.29 (s) ppm.

^{13}C NMR ($CDCl_3$, 125 MHz): δ 22.2, 29.5, 35.7, 42.2, 46.8, 58.7, 67.2, 121.4, 123.6, 125.8 (m), 127.3 (m), 127.4, 127.5, 128.1, 132.3, 132.6, 132.8, 133.1 (q, *J* = 34.3 Hz), 142.6 ppm.

Solvents peaks: chloroform-*d* 77.1 ppm (t).

IR (KBr): 3430, 3310, 2969, 1360, 1280, 1133, 682, 589 cm^{-1}.

HRMS (ESI+, *m/z*): calcd for $[C_{23}H_{25}F_6N_2O_2S]^+$ ($[M + H]^+$) 507.1541; found 507.1553.

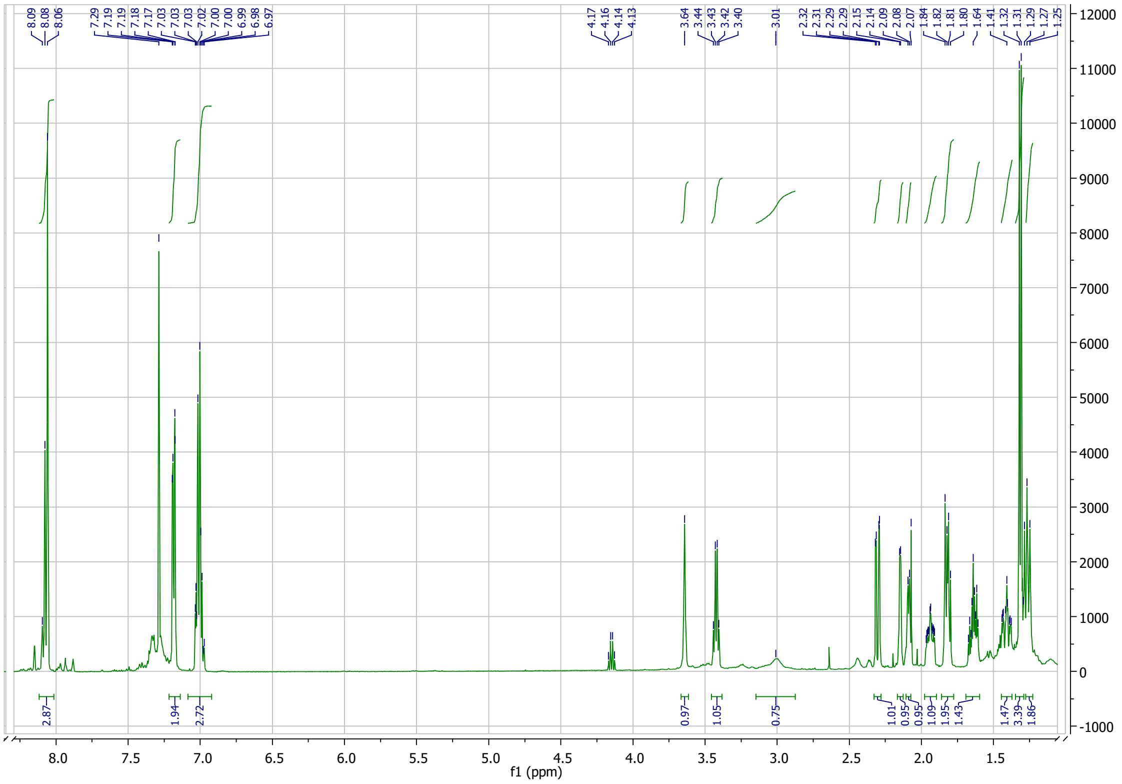

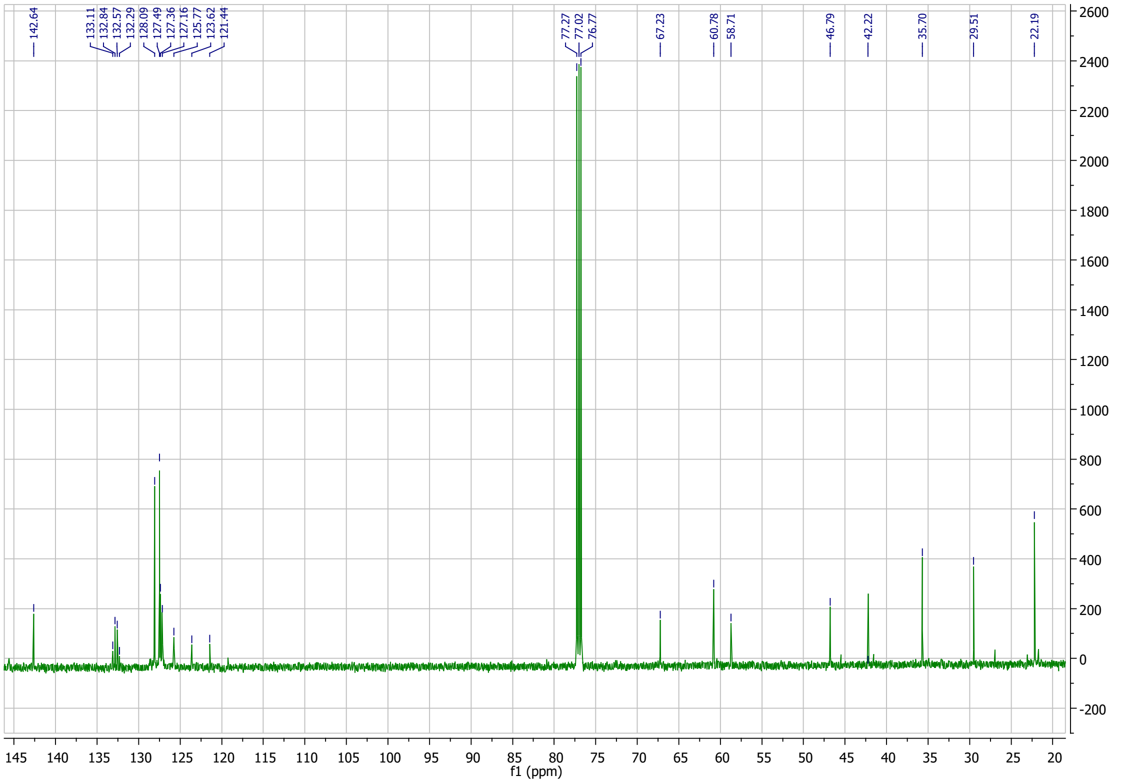

***N*-((2-((S)-1-Phenylethyl)-(1S,3R,4R)-2-azabicyclo[2.2.1]heptan-3-yl)-[(1R)-(-)-10-camphor]sulfonamide (10h)**

Yellow oil. Yield 0.27 g (61%). $[\alpha]_D^{20}$ = +23.0 (*c* 0.50, CH_2Cl_2). ^{1}H NMR (600 MHz, $CDCl_3$): δ 0.76 (s, 3H), 0.91 (s, 3H), 1.24-1.30 (m, 7H), 1.59-1.66 (m, 1H), 1.70-1.77 (m, 2H), 1.82-1.87 (m, 1H), 1.90-1.99 (m, 2H), 2.02-2.15 (m, 4H), 2.25-2.34 (m, 3H), 2.50, 2.86 (AB system, *J* = 14.8 Hz, 2H), 3.46 (q, *J* = 7.0 Hz, 1H), 3.59 (s, 1H), 4.75 (s, 1H, NH), 7.15-7.33 (m, 5H, ArH) ppm.

Solvents peaks: ethyl acetate 1.21-1.25 (t), 2.02 (s), 4.07-4.12 (q) ppm.

^{13}C NMR ($CDCl_3$, 125 MHz): δ 19.6, 20.0, 22.4, 22.6, 26.4, 27.0, 29.1, 35.4, 40.7, 42.8, 47.3, 48.4, 48.5, 58.8, 58.9, 61.0, 127.4, 128.0, 128.4, 146.2, 216.0 ppm.

Solvents peaks: chloroform-*d* 77.1 ppm (t).

IR (KBr): 3449, 3261, 2955, 2871, 1735, 1452, 1328 1149, 1067, 701 cm^{-1}.

HRMS (ESI+, *m/z*): calcd for $[C_{25}H_{37}N_2O_3S]^+$ ($[M + H]^+$) 445.2521; found 445.2525.

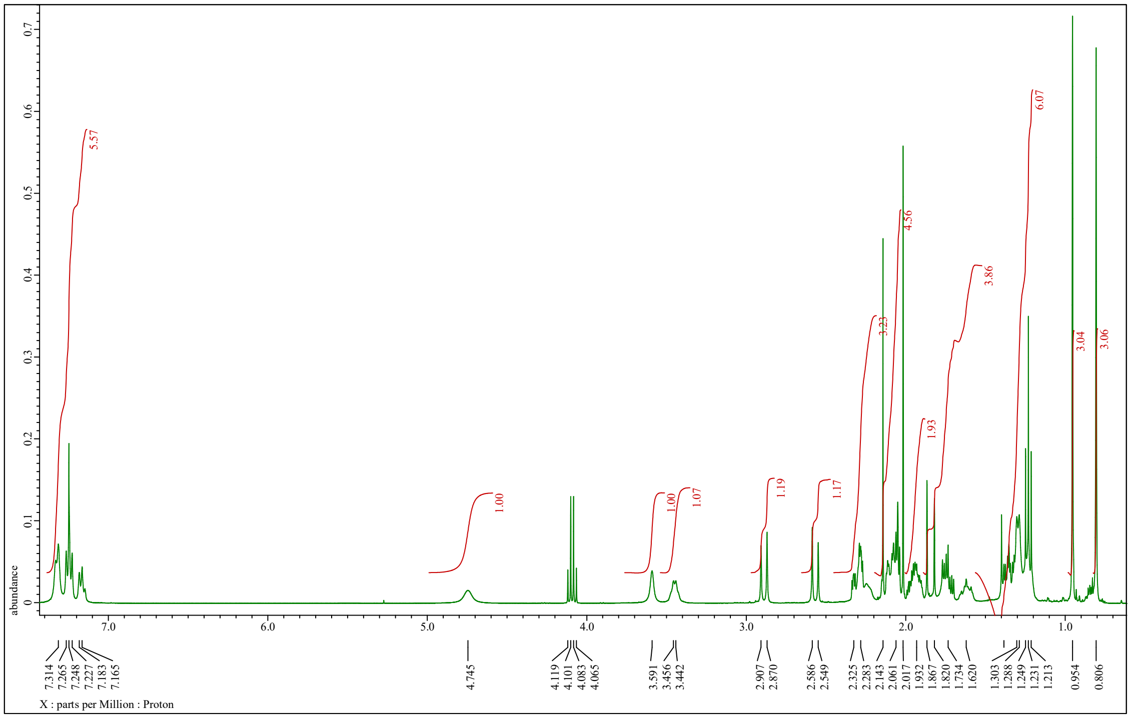

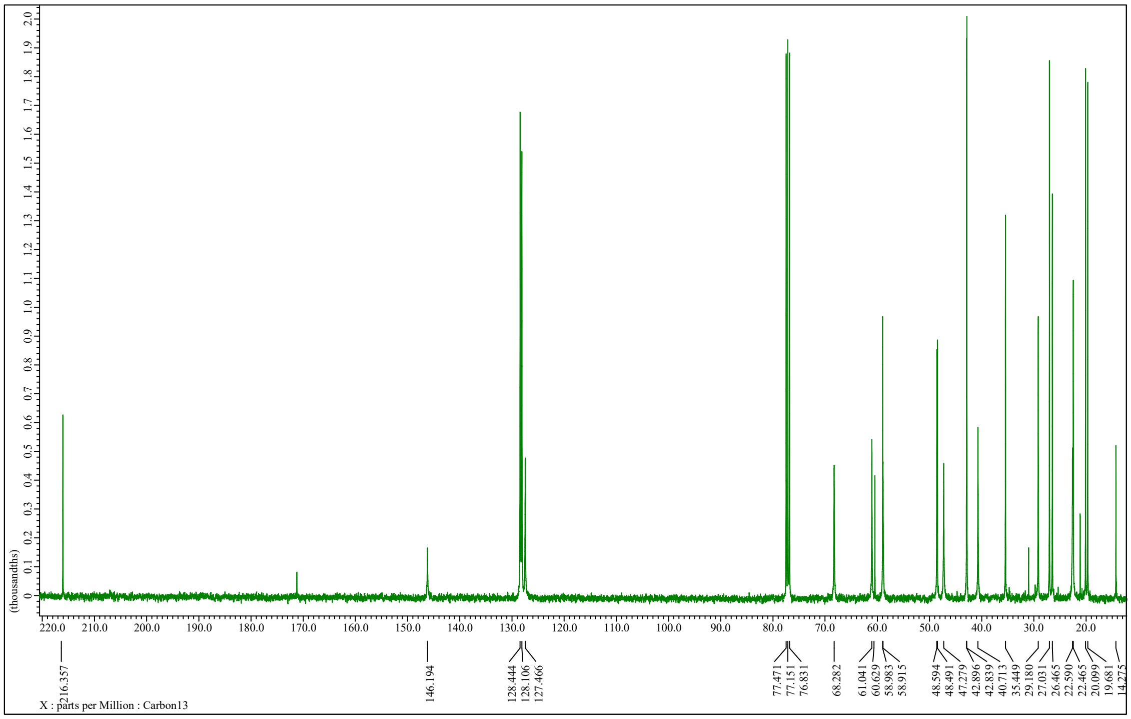

4-Phenyl-*N*-((2-((*S*)-1-phenylethyl)-(1*S*,4*S*,5*R*)-2-azabicyclo[3.2.1]octan-4-yl) benzenesulfonamide (11a)

Yellow oil. Yield 0.34 g (76%). $[\alpha]_D^{20} = -13.5$ (*c* 0.50, CH_2Cl_2). ^1H NMR (600 MHz, $CDCl_3$): δ 1.22-1.37 (m, 7H), 1.57-1.68 (m, 2H), 1.87 (d, *J* = 19.8 Hz, 2H), 2.29 (q, *J* = 6.6 Hz, 1H), 3.03 (m, 1H), 3.20 (q, *J* = 10.2 Hz, 1H), 3.52 (t, *J* = 7.8 Hz, 1H), 5.05 (d, *J* = 12.6 Hz, 1H), 7.14-7.26 (m, 6H, ArH), 7.38-7.54 (m, 6H, ArH), 7.65-7.67 (m, 2H, ArH) ppm.

Solvents peaks: ethyl acetate 1.23-1.26 (t), 2.03 (s), 4.08-4.14 (q); chloroform 7.25 (s) ppm.

^{13}C NMR ($CDCl_3$, 125 MHz): δ 14.3, 21.3, 21.8, 27.2, 34.4, 40.1, 49.1, 52.7, 55.7, 62.3, 127.0, 127.2, 127.3, 127.6, 128.4, 128.5, 129.1, 139.3, 139.4, 145.0, 145.3.

Solvents peaks: chloroform-*d* 77.1 ppm (t).

IR (KBr): 3435, 3288, 2934, 1321, 1161, 1093, 763, 673, 587 cm^{-1}. HRMS (ESI+, *m/z*): calcd for $[C_{27}H_{31}N_2O_2S]^+$ ($[M+H]^+$) 447.2109; found 447.2106.

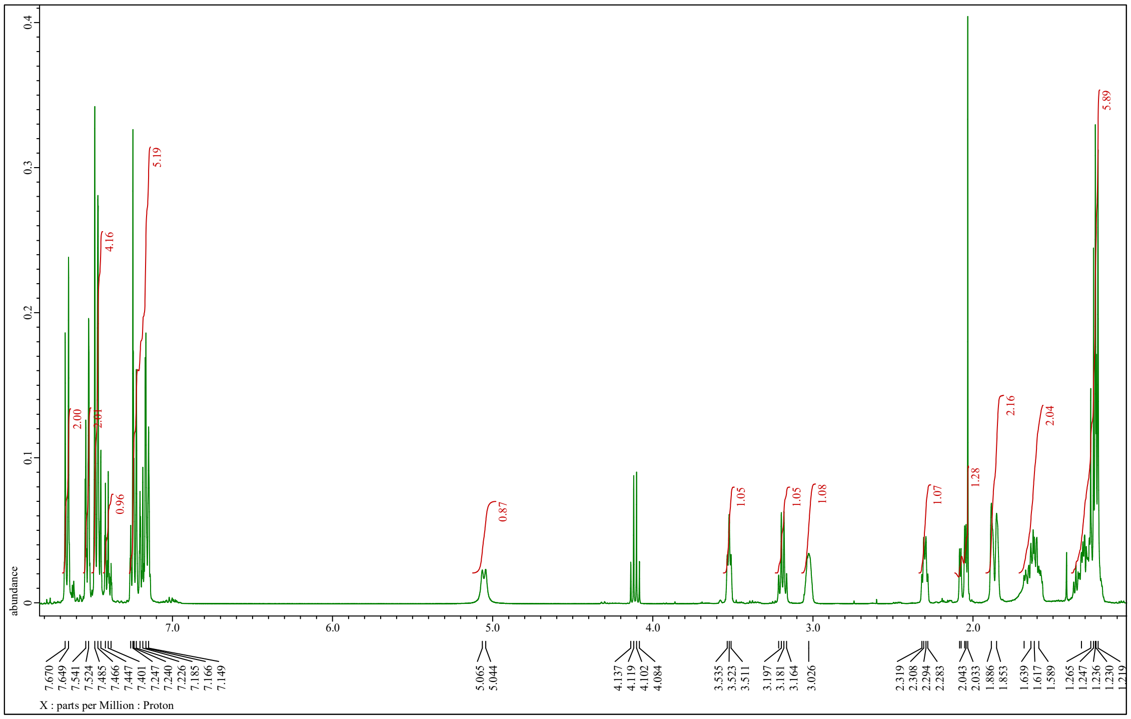

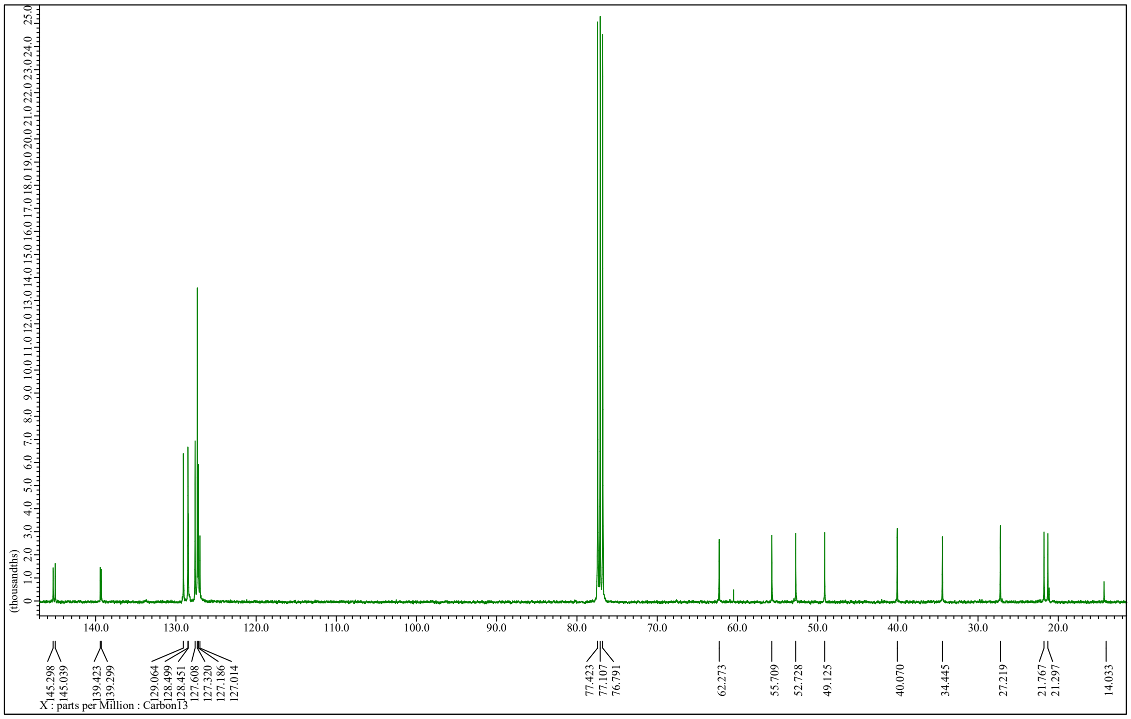

4-(4-Methoxyphenyl)-*N*-((2-((*S*)-1-phenylethyl)-(1*S*,4*S*,5*R*)-2-azabicyclo[3.2.1]octan-4-yl)benzenesulfonamide (11e)

White solid. Yield 0.30 g (63%). Mp 153-155 °C. $[\alpha]_D^{20} = -7.8$ (*c* 1.00, CH_2Cl_2). ^{1}H NMR (600 MHz, $CDCl_3$): δ 1.19-1.37 (m, 6H), 1.56-1.68 (m, 2H), 1.85-1.88 (m, 2H, 2.03-2.08 (m, 1H), 2.30 (q, *J* = 7.2 Hz, 1H), 3.01 (m, 1H), 3.18 (q, *J* = 9.6 Hz, 1H), 3.52 (t, *J* = 4.4 Hz, 1H), 3.86 (s, 3H), 5.02 (d, *J* = 13.2 Hz, 1H), 6.98-7.00 (m, 2H), 7.14-7.26 (m, 6H), 7.41-7.49 (m, 3H), 7.61-7.64 (m, 2H) ppm.

Solvents peaks: chloroform 7.24 (s) ppm.

^{13}C NMR ($CDCl_3$, 125 MHz): δ 21.3, 21.8, 27.2, 34.5, 40.1, 49.1, 52.7, 55.5, 55.7, 62.3, 114.5, 127.0, 127.2, 127.3, 128.4, 128.5, 131.8, 138.5, 144.6, 145.3, 160.1 ppm.

Solvents peaks: chloroform-*d* 77.1 ppm (t).

IR (KBr): 3441, 3254, 2938, 1607, 1307, 1247, 1157, 1041, 822, 642 cm^{-1}.

HRMS (ESI+, *m/z*): calcd for $[C_{28}H_{33}N_2O_3S]^+$ ($[M+H]^+$) 477.2220; found 477.2212.

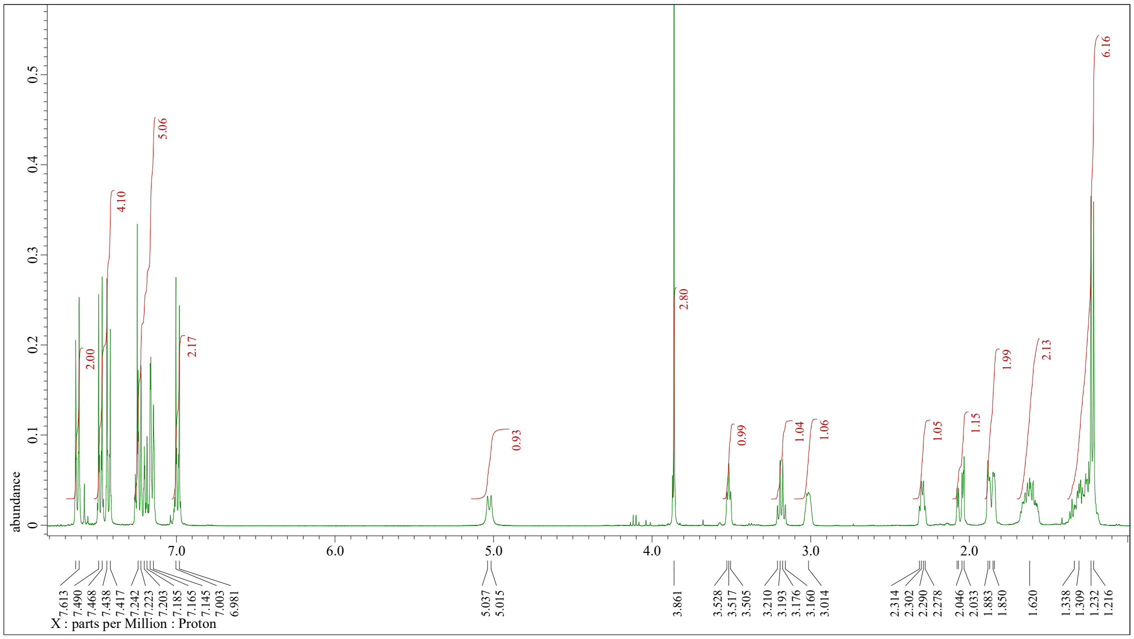

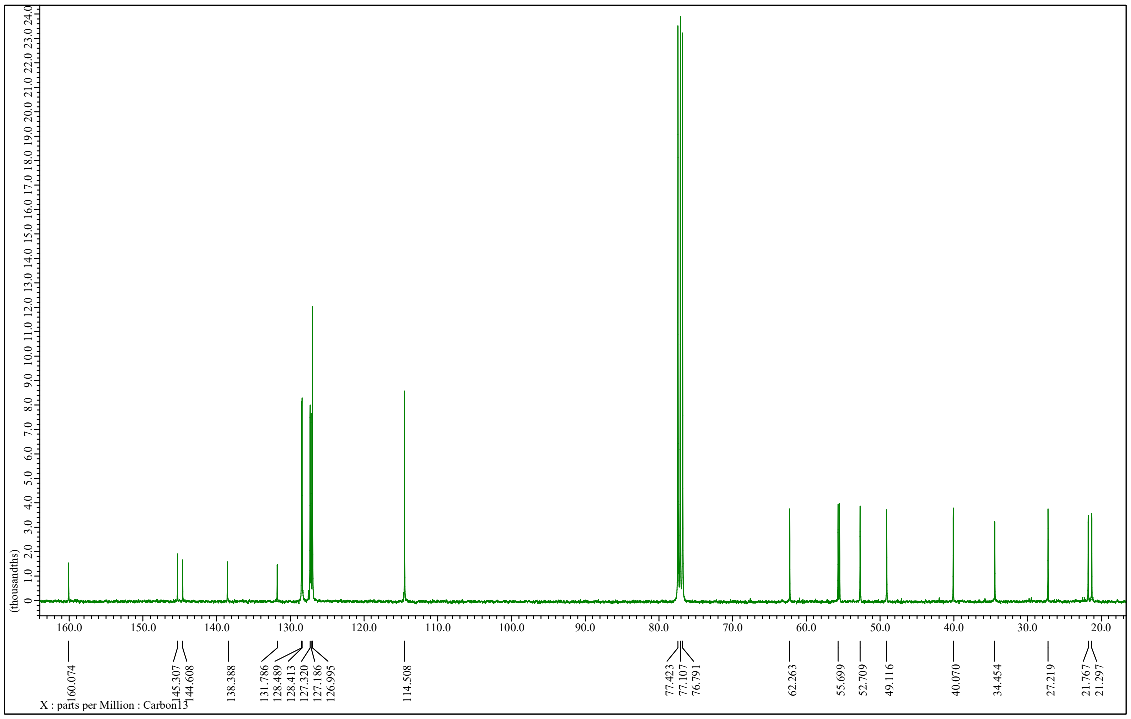

4-Trifluoromethyl-*N*-((2-((*S*)-1-phenylethyl)-(1*S*,4*S*,5*R*)-2-azabicyclo[3.2.1]octan-4-yl)benzenesulfonamide (11f)

Colorless oil. Yield 0.25 g (57%). $[\alpha]_D^{20} = -36.9$ (*c* 1.07, CH_2Cl_2). ^1H NMR (600 MHz, $CDCl_3$): δ 1.21-1.38 (m, 7H), 1.59-1.84 (m, 4H), 2.01-2.05 (m, 1H), 2.28 (q, *J* = 6.6 Hz, 1H), 3.01 (s, 1H), 3.14 (q, *J* = 10.2 Hz, 1H), 3.54 (t, *J* = 6.6 Hz, 1H), 5.15 (s, 1H), 7.12-7.14 (m, 2H, ArH), 7.22-7.26 (m, 3H, ArH), 7.50-7.52 (d, *J* = 8.4 Hz, 2H, ArH), 7.69-7.71 (d, *J* = 8.8 Hz, 2H, ArH) ppm.

Solvents peaks: ethyl acetate 1.24-1.26 (t), 2.03 (s), 4.08-4.13 (q) ppm, chloroform 7.25 (s, overlapped).

^{13}C NMR ($CDCl_3$, 125 MHz): δ 14.3, 21.1, 21.3, 21.5, 21.6 (m), 27.1, 27.2 (m), 34.3, 34.4, 34.5 (m), 40.1, 49.0, 49.1, 49.3 (m), 52.7, 52.8, 52.9 (m), 55.4, 55.5 (d, *J* = 6.0 Hz), 60.5, 62.1, 62.2 (m), 121.9, 124.6, 126.1, 126.2 (m), 127.1, 127.2 (m), 128.4, 128.5, 128.6 (m), 133.7, 134.0 (q, *J* = 40.7 Hz), 144.3, 145.3 ppm.

Solvents peaks: chloroform-*d* 77.1 ppm (t).

IR (KBr): 3432, 3301, 2965, 1333, 1322, 1160, 1062, 711, 596 cm^{-1}.

HRMS (ESI+, *m/z*): calcd for $[C_{22}H_{26}F_3N_2O_2S]^+$ ($[M+H]^+$) 439.1667; found 439.1670.

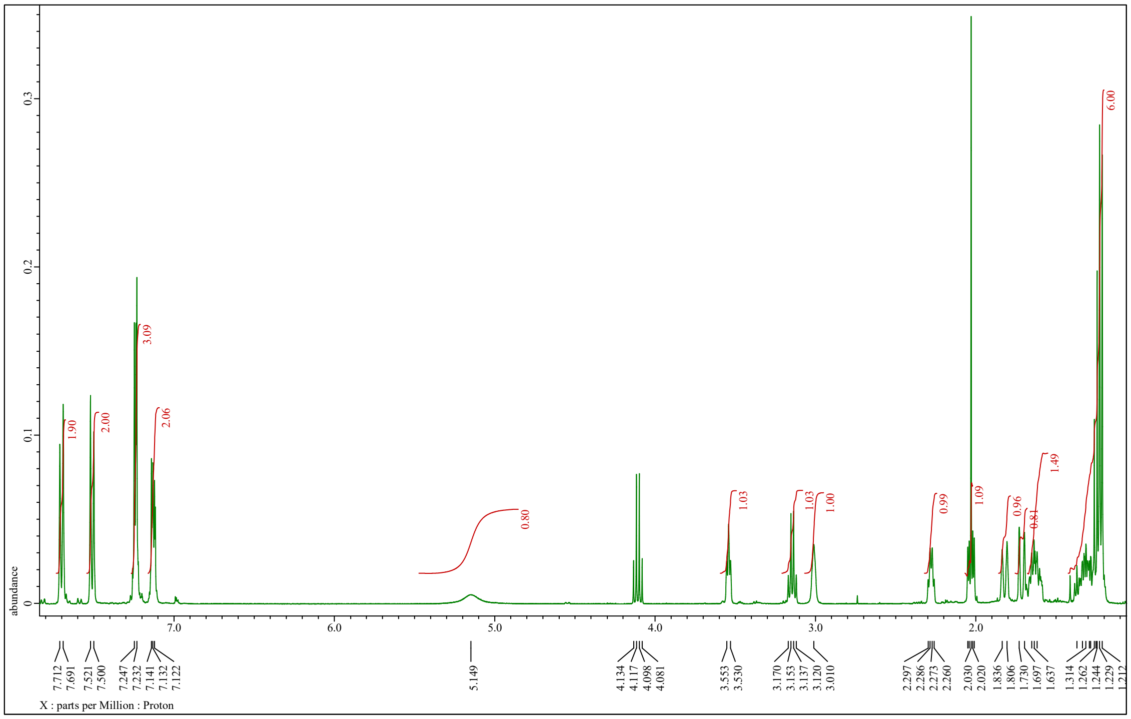

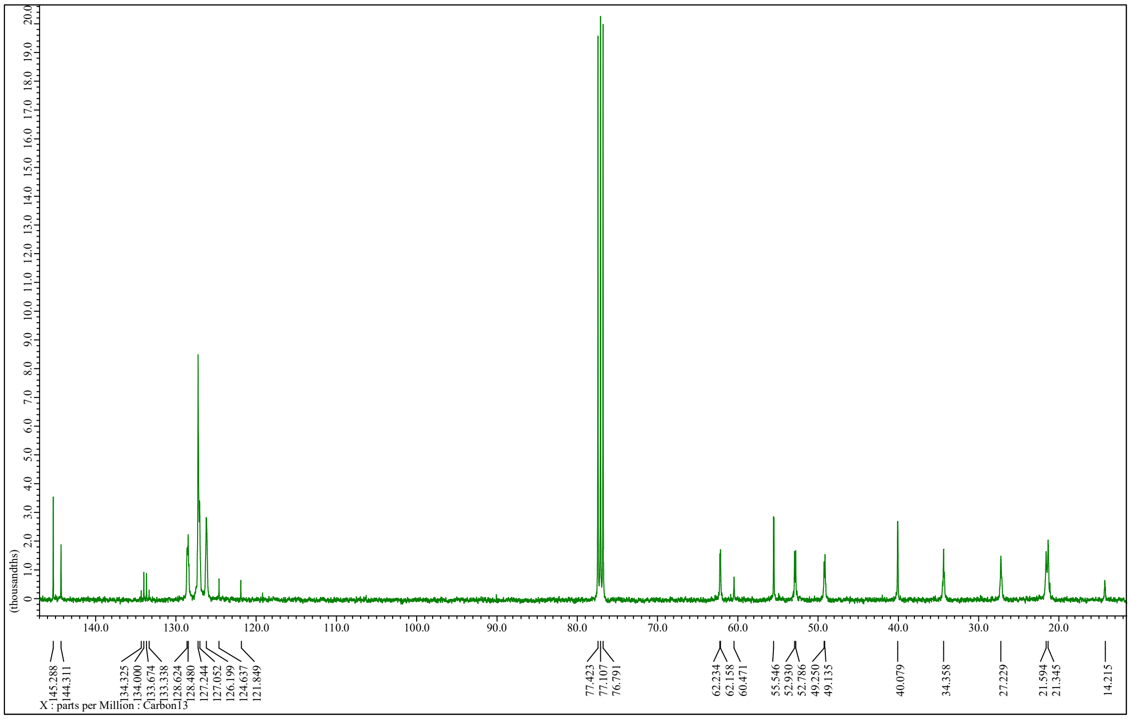

3,5-Bis(trifluoromethyl)-N-((2-((S)-1-phenylethyl)-(1S,4S,5R)-2-azabicyclo[3.2.1]octan-4-yl)benzenesulfonamide (11g)

White solid. Yield 0.29 g (57%). Mp 116-118 °C. $[\alpha]_D^{20} = -36.9$ (*c* 0.79, CH_2Cl_2). ^{1}H NMR (600 MHz, $CDCl_3$): δ 1.23-1.42 (m, 7H), 1.59-1.70 (m, 2H), 1.78-1.99 (m, 1H), 2.19-2.25 (m,

1H), 3.18 (s, 1H), 3.24 (q, J = 10.2 Hz, 1H), 3.53 (t, J = 7.2 Hz, 1H), 5.08 (s, 1H, NH), 7.10-7.25 (m, 5H, ArH), 7.94 (s, 1H, ArH), 8.15 (s, 2H, ArH) ppm.

Solvents peaks: ethyl acetate 1.26-1.28 (t), 2.03 (s), 4.08-4.14 (q), chloroform 7.25 (s) ppm.

^{13}C NMR ($CDCl_3$, 125 MHz): δ 21.0, 21.8, 27.2, 34.3, 39.9, 49.2, 53.3, 56.0, 62.2, 121.1, 123.8, 125.8, 125.91, 125.94, 126.0 (q, J = 4.9 Hz), 127.0, 127.1, 128.4, 132.3, 132.6, 133.0, 133.3 (q, J = 42.0 Hz), 144.3, 144.4 ppm.

Solvents peaks: chloroform-*d* 77.1 ppm (t).

IR (KBr): 3349, 2963, 1342, 1283, 1158, 907, 700, 592 cm^{-1}.

HRMS (ESI+, *m/z*): calcd for $[C_{23}H_{25}F_6N_2O_2S]^+$ ($[M+H]^+$) 507.1541; found 507.1539.

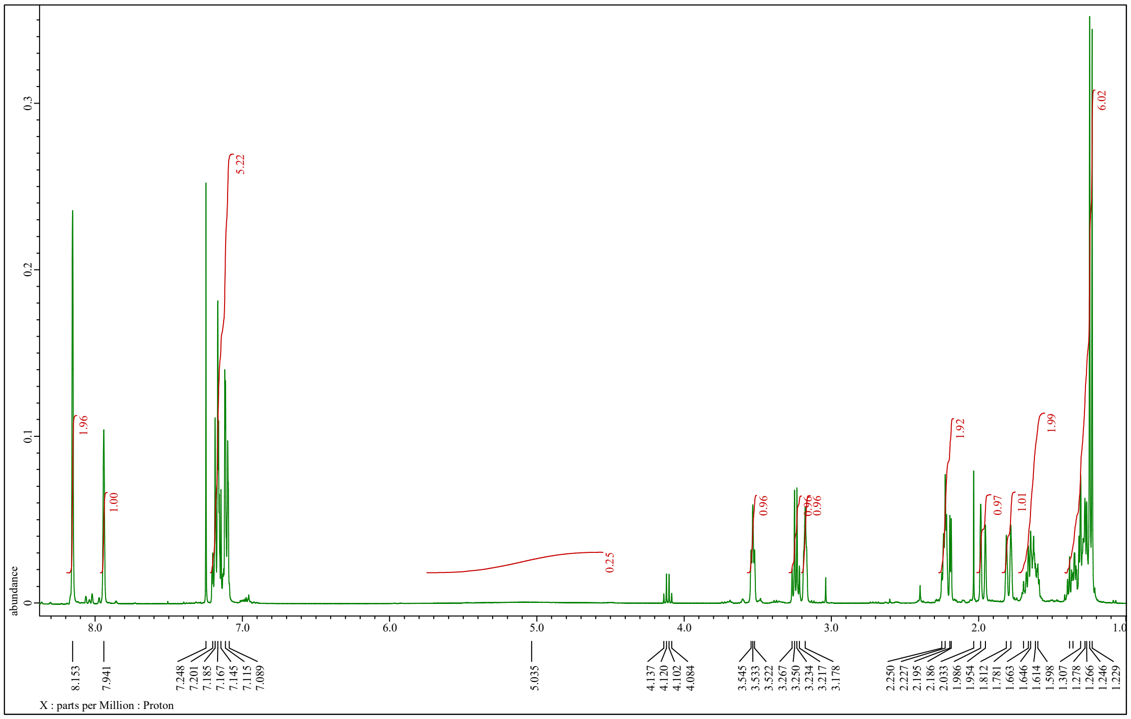

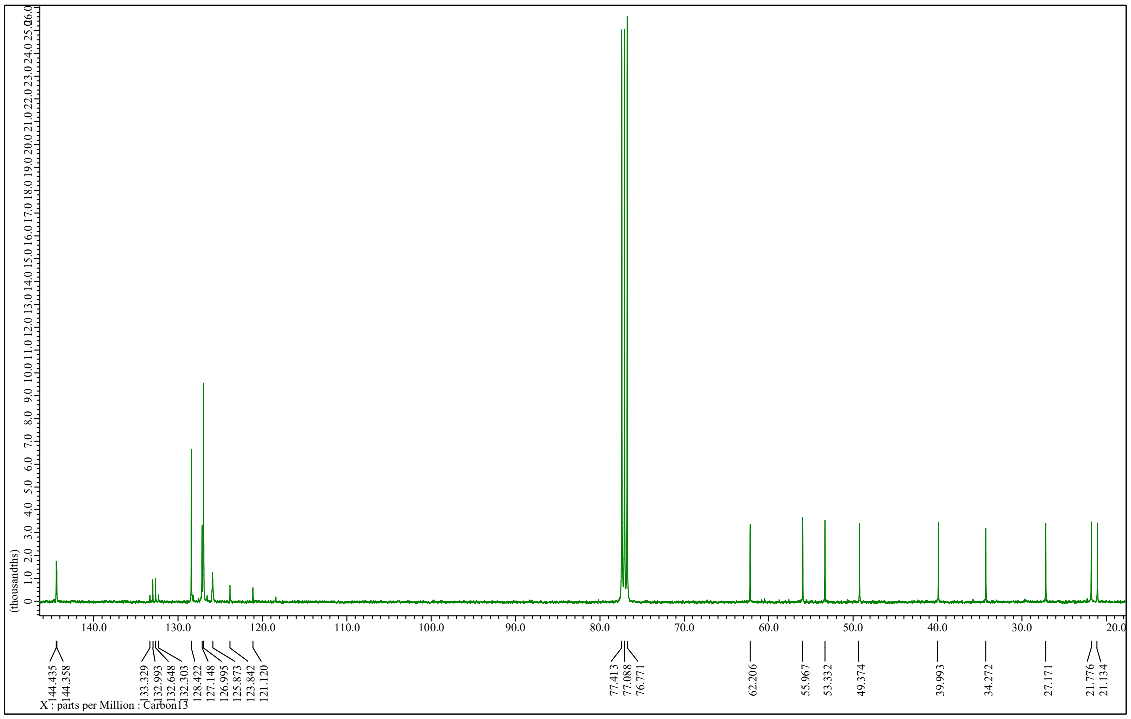

***N*-((2-((S)-1-Phenylethyl)-(1S,4S,5R)-2-azabicyclo[3.2.1]octan-4-yl)-[(1R)-(-)-10-camphore]sulfonamide (11h)**

Yellow oil. Yield 0.19 g (43%). $[\alpha]_D^{20} = -10.9$ (*c* 0.41, CH_2Cl_2). ^1H NMR (600 MHz, $CDCl_3$): δ 0.82 (s, 3H), 0.99 (s, 3H), 1.23-1.40 (m, 9H), 1.65-1.70 (m, 1H), 1.72-2.15 (m, 6H), 2.22-2.39 (m, 4H), 2.78 (d, J = 22.2 Hz, 1H), 3.27-3.35 (m, 2H), 3.53 (s, 1H), 5.45 (s, 1H), 7.18-7.27 (m, 5H, ArH) ppm.

Solvents peaks: ethyl acetate 1.22-1.25 (t), 2.02 (s), 4.07-4.12 (q), chloroform 7.25 (s) ppm.

^{13}C NMR ($CDCl_3$, 125 MHz): δ 19.7, 19.9, 21.0, 22.1, 26.0, 27.0, 27.4, 34.3, 39.8, 42.8, 42.9, 48.3, 50.0, 51.4, 53.1, 56.1, 59.0, 62.4, 127.0, 127.3, 128.5, 145.1, 215.8 ppm.

Solvents peaks: chloroform-*d* 77.1 ppm (t).

IR (film): 3448, 3271, 2969, 2943, 1739, 1410, 1338, 1135, 771, 700, 562 cm^{-1}.

HRMS (ESI+, *m/z*): calcd for $[C_{25}H_{37}N_2O_3S]^+$ ($[M+H]^+$) 445.2525; found 445.2521.

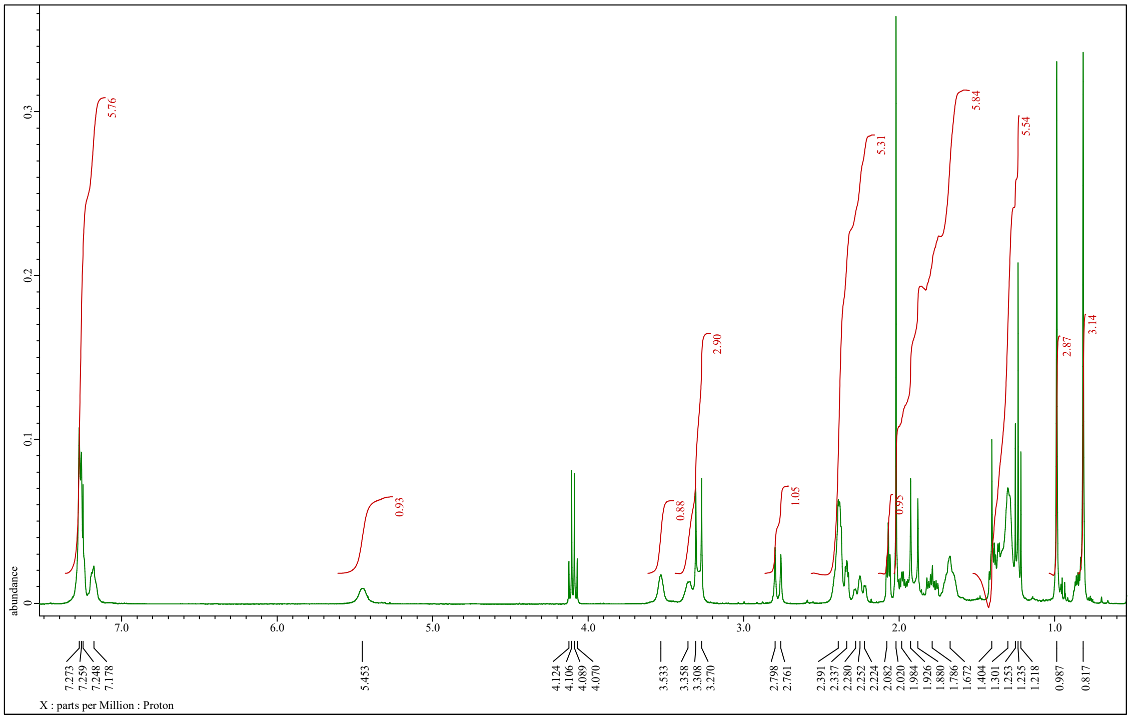

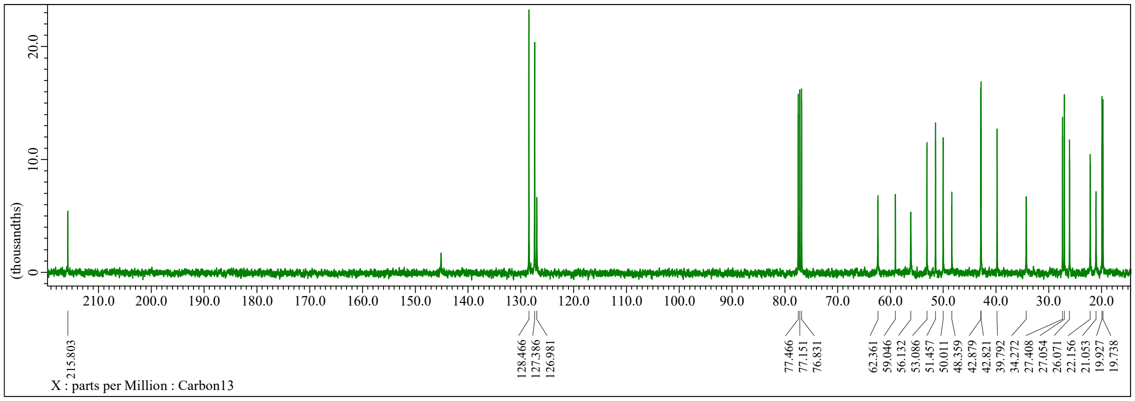

Bis-*N*,*N*-{[(1*S*,3*R*,4*R*)-2-((*S*)-1-phenylethyl)-2-azabicyclo[2.2.1]heptan-3-yl]methyl}-[1,1'-biphenyl]ethyl-4-sulfonamide (12a)

White solid. Yield 0.60 g (66%). Mp 118-120 ºC. $[\alpha]_D^{20}$ = +76.1 (*c* 0.8, CH_2Cl_2). ^{1}H NMR (400 MHz, $CDCl_3$): δ 1.27-1.29 (d, *J* = 6.4 Hz, 6H), 1.38-1.42 (m, 5H), 1.62 (s, 2H), 1.69-1.75 (m, 7H), 2.29-2.30 (s, 2H), 2.36-2.52 (m, 6H), 2.70-2.76 (m, 3H), 3.50-3.55 (q, *J* = 6.4 Hz, 2H), 3.63 (s, 2H), 7.12-7.16 (m, 2H, ArH), 7.20-7.24 (m, 6H, ArH), 7.36-7.38 (m, 3H, ArH), 7.44-7.45 (m, 6H, ArH), 7.48-7.52 (m, 4H, ArH), 7.59-7.61 (m, 8H, ArH) ppm.

Solvents peaks: ethyl acetate 1.23-1.26 (t), 2.03 (s), 4.08-4.14 (q); methylene chloride 5.28 (s); chloroform 7.25 (s) ppm.

^{13}C NMR (125 MHz, $CDCl_3$): δ 22.8, 28.7, 35.0, 38.8, 49.6, 54.2, 59.4, 61.0, 67.2, 127.2, 127.4, 127.6, 127.8, 128.4, 128.6, 128.6, 129.2, 136.4, 139.5, 145.4, 146.3 ppm.

Solvents peaks: chloroform-*d* 77.1 ppm (t).

IR (KBr): 3435, 2968, 2870, 1595, 1481, 1453, 1347, 1309, 1162, 763, 728, 700, 666, 586 cm^{-1}.
HRMS (ESI+, *m/z*): calcd for $[C_{56}H_{62}N_4O_4S_2]^+$ ($[M+H]^+$) 919.4285; found: 919.4293.

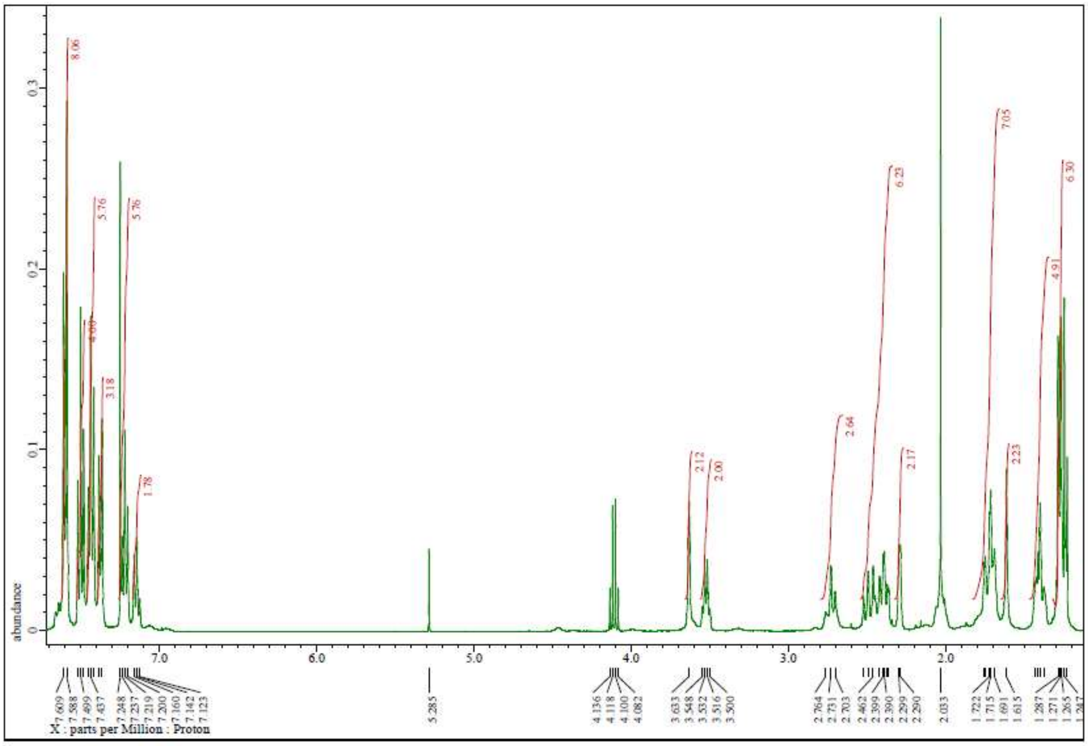

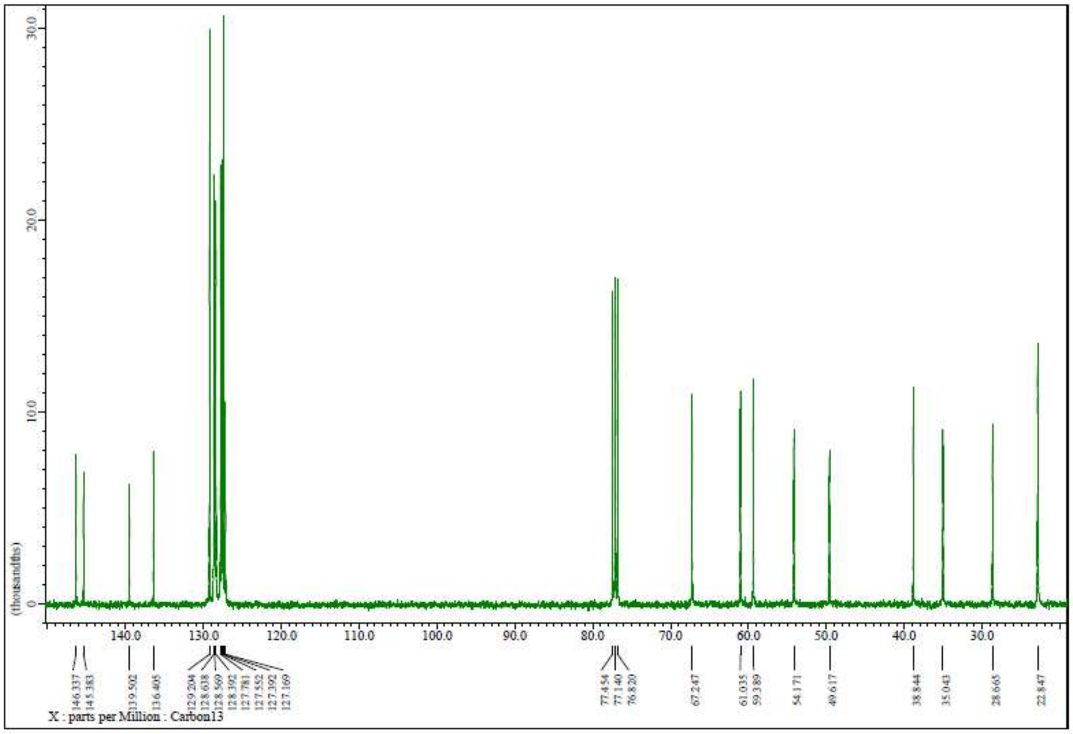

Bis-4’-methyl-N,N-{[(1*S*,3*R*,4*R*)-2-((*S*)-1-phenylethyl)-2-azabicyclo[2.2.1]heptan-3-yl]methyl}-[1,1'-biphenyl]ethyl-4-sulfonamide (12b)

White solid. Yield 0.24 g (62%). Mp 120-125 ºC. $[\alpha]_D^{20}$ = +78.1 (*c* 0.7, CH_2Cl_2). ^1H NMR (400 MHz, $CDCl_3$): δ 1.24 (s, 1H), 1.27-1.29 (d, J = 6.4 Hz, 7H), 1.37-1.42 (m, 5H), 1.61 (s, 1H), 1.69-1.75 (m, 7H), 2.01-2.06 (m, 3H), 2.29-2.30 (s, 2H), 2.36-2.40 (m, 1H), 2.43 (s, 6H), 2.46-2.52 (m, 1H), 2.70-2.72 (m, 2H), 3.50-3.55 (q, J = 6.4 Hz, 2H), 3.63 (s, 2H), 7.12-7.15 (m, 2H, ArH), 7.19-7.23 (m, 3H, ArH), 7.29-7.31 (m, 4H, ArH), 7.36-7.42 (m, 8H, ArH), 7.49-7.51 (m, 5H, ArH), 7.57-7.59 (m, 4H, ArH) ppm.

Solvents peaks: methylene chloride 5.29 (s), chloroform 7.25 (s) ppm.

^{13}C NMR (125 MHz, $CDCl_3$): δ 21.3, 22.9, 28.6, 35.0, 38.8, 49.6, 54.1, 59.4, 61.0, 67.2, 127.2, 127.2, 127.3, 127.7, 128.4, 128.6, 129.9, 136.1, 136.6, 138.6, 145.3, 146.3 ppm.

Solvents peaks: chloroform-*d* 77.1 ppm (t).

IR (KBr): 3436, 2969, 2870, 1596, 1490, 1453, 1345, 1311, 1163, 809, 752, 702, 631, 564 cm^{-1}. HRMS (ESI+, *m/z*): calcd for $[C_{58}H_{66}N_4O_4S_2]^+$ ($[M+H]^+$) 947.4598; found 947.4591.

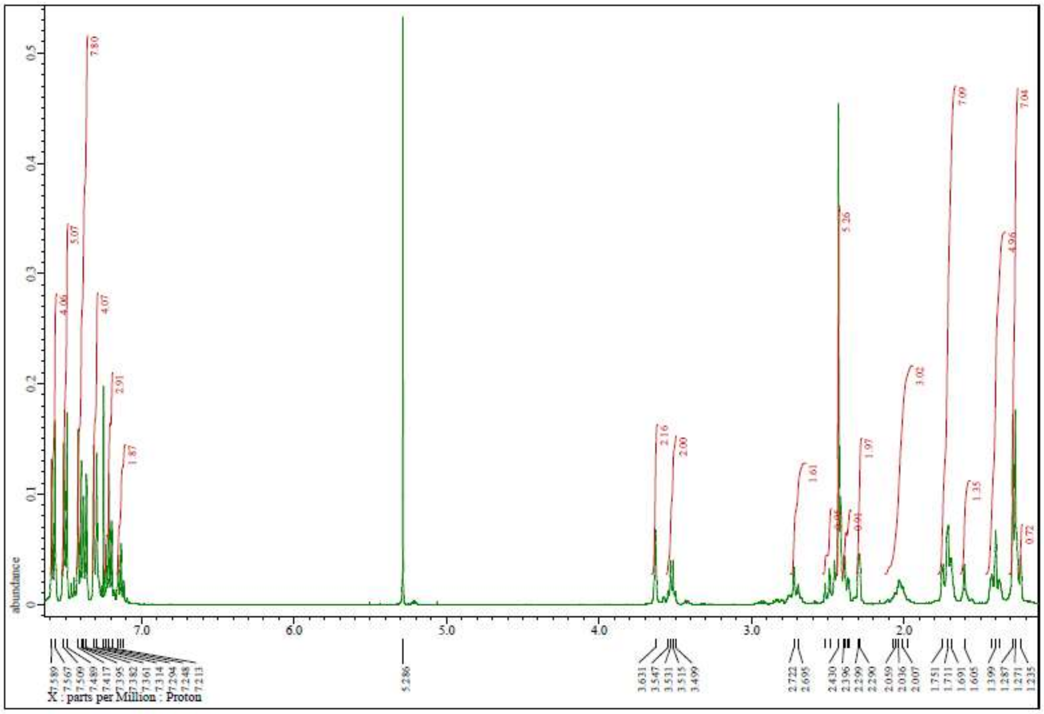

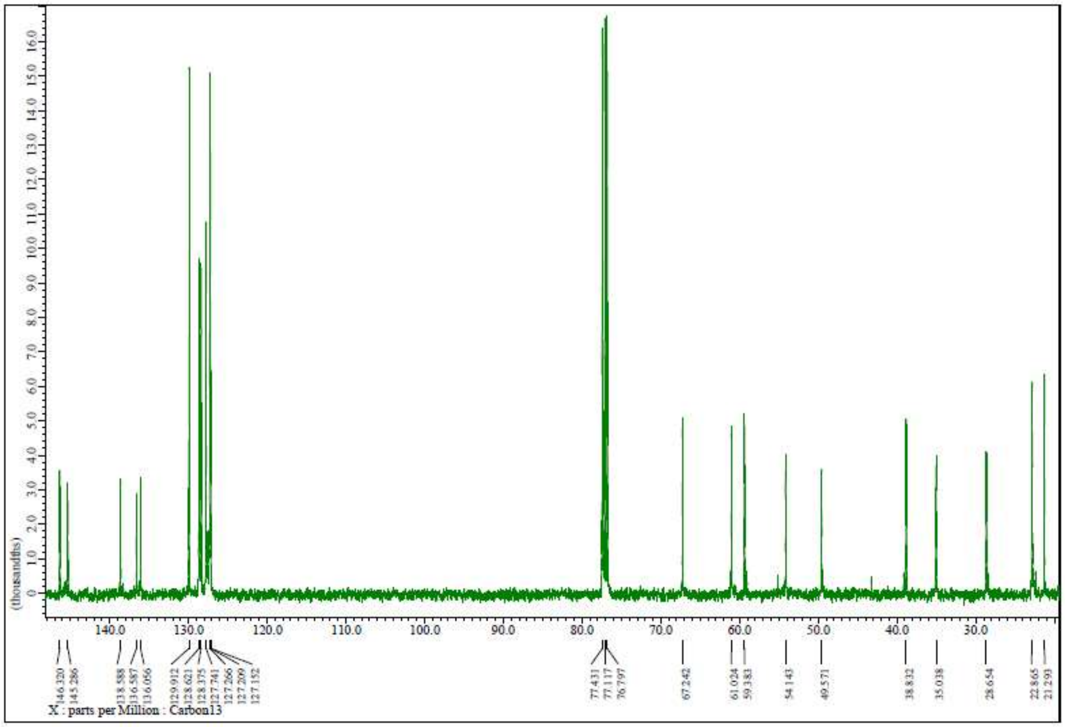

Bis-4'-chloro-*N*,*N*-{[(1*S*,3*R*,4*R*)-2-((*S*)-1-phenylethyl)-2-azabicyclo[2.2.1]heptan-3-yl]methyl}-[1,1'-biphenyl]ethyl-4-sulfonamide (12d)

White solid. Yield 0.26 g (67%). Mp 252-255 ºC. $[\alpha]_D^{20}$ = +84.3 (*c* 0.6, CH_2Cl_2). ^1H NMR (400 MHz, $CDCl_3$): δ 1.24 (s, 1H), 1.27-1.29 (d, *J* = 6.4 Hz, 6H), 1.38-1.43 (m, 4H), 1.61 (s, 1H), 1.68-1.75 (m, 6H), 2.00-2.06 (m, 2H), 2.28-2.29 (s, 2H), 2.35-2.52 (m, 6H), 2.69-2.79 (m, 2H), 3.50-3.55 (q, *J* = 6.4 Hz, 2H), 3.63 (s, 2H), 7.12-7.16 (m, 2H, ArH), 7.20-7.24 (m, 5H, ArH), 7.36-7.38 (m, 4H, ArH), 7.42-7.48 (m, 8H, ArH), 7.51-7.57 (m, 8H, ArH) ppm.

Solvents peaks: methylene chloride 5.28 (s), chloroform 7.24 (s) ppm.

^{13}C NMR (125 MHz, $CDCl_3$): δ 22.9, 28.7, 35.0, 38.8, 49.7, 54.2, 59.4, 61.0, 67.2, 127.1, 127.4, 127.9, 128.4, 128.6, 129.4, 134.9, 136.7, 137.9, 144.1, 146.3 ppm.

Solvents peaks: chloroform-*d* 77.1 ppm (t).

IR (KBr): 3435, 2963, 2872, 1596, 1478, 1454, 1344, 1306, 1163, 1093, 817, 781, 738, 706, 604 cm^{-1}.

HRMS (ESI+, *m/z*): calcd for $[C_{56}H_{60}Cl_2N_4O_4S_2]^+$ ($[M+H]^+$) 989.3506; found 989.3466.

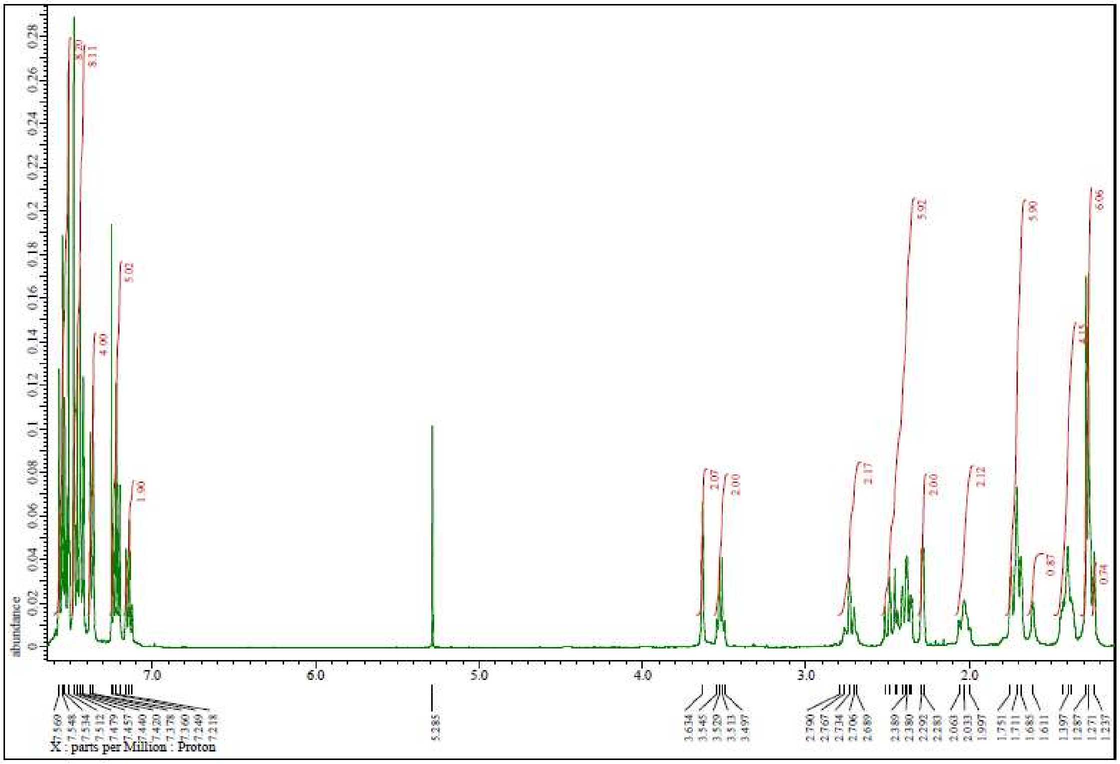

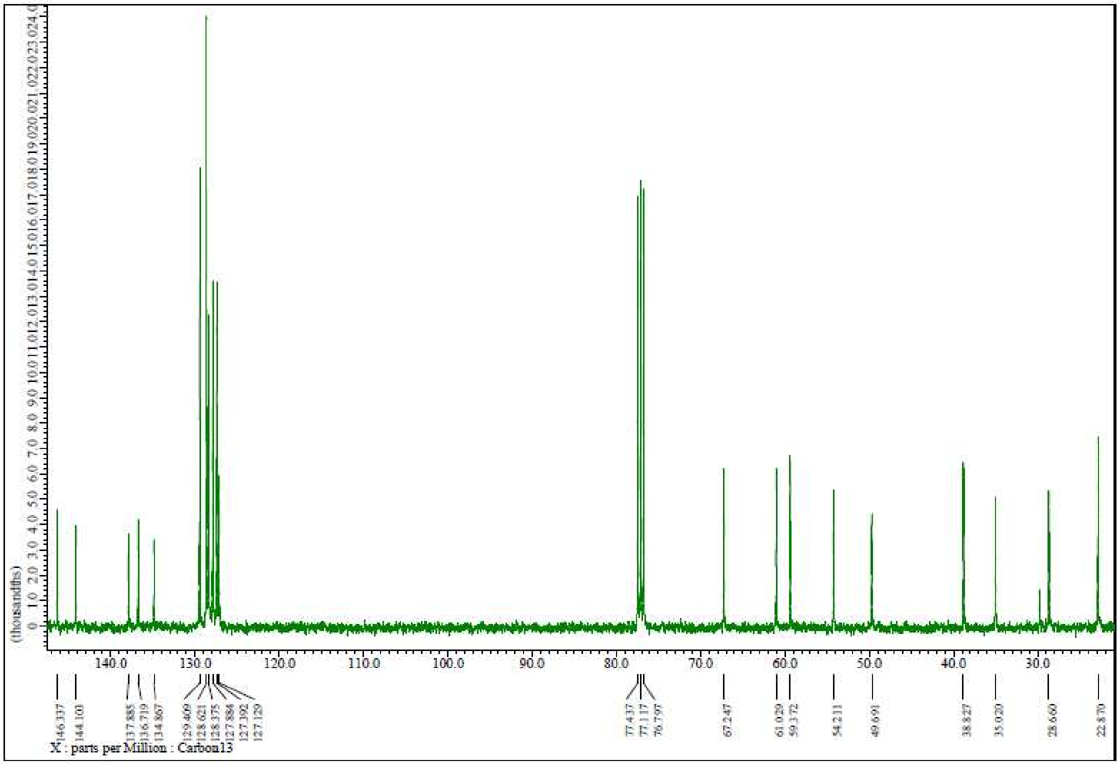

Bis-*N*,*N*-(4-(*N*-(((1*S*,3*R*,4*R*)-2-((*S*)-1-phenylethyl)-2-azabicyclo[2.2.1]heptan-3-yl)methyl)sulfamoyl)ethyl)phenyl)acetamide (12i)

White solid. Yield 0.13 g (37%). Mp 145-147 °C. $[\alpha]_D^{20}$ = +43.5 (*c* 0.4, CH_2Cl_2). ^{1}H NMR (400 MHz, $CDCl_3$): δ 1.24-1.29 (m, 8H), 1.33-1.43 (m, 1H), 1.68-1.75 (m, 7H), 1.98-2.02 (m, 2H), 2.19 (s, 6H), 2.26-2.34 (m, 6H), 2.44-2.50 (m, 2H), 2.67-2.72 (m, 2H), 3.48-3.53 (q, *J* = 6.4 Hz, 2H), 3.66 (s, 2H), 7.11-7.13 (m, 2H, ArH), 7.17-7.25 (m, 8H, ArH), 7.33-7.35 (m, 4H, ArH), 7.53-7.55 (m, 4H, ArH), 8.16 (s, 2H) ppm.

Solvents peaks: chloroform 7.24 (s) ppm.

^{13}C NMR (125 MHz, $CDCl_3$): δ 22.8, 24.8, 28.6, 35.0, 38.8, 49.2, 53.9, 59.5, 61.1, 67.6, 119.4, 128.4, 128.3, 128.5, 128.6, 132.8, 141.8, 146.2, 168.9 ppm.

Solvents peaks: chloroform-*d* 77.1 ppm (t).

IR (KBr): 3354, 2970, 2871, 1706, 1683, 1593, 1530, 1493, 1453, 1402, 1343, 1312, 1158, 1091, 840, 738, 703, 615 cm^{-1}.

HRMS (ESI+, *m/z*): calcd for $[C_{48}H_{60}N_6O_6S_2]^+$ ($[M+H]^+$) 881.4088; found 881.4103.

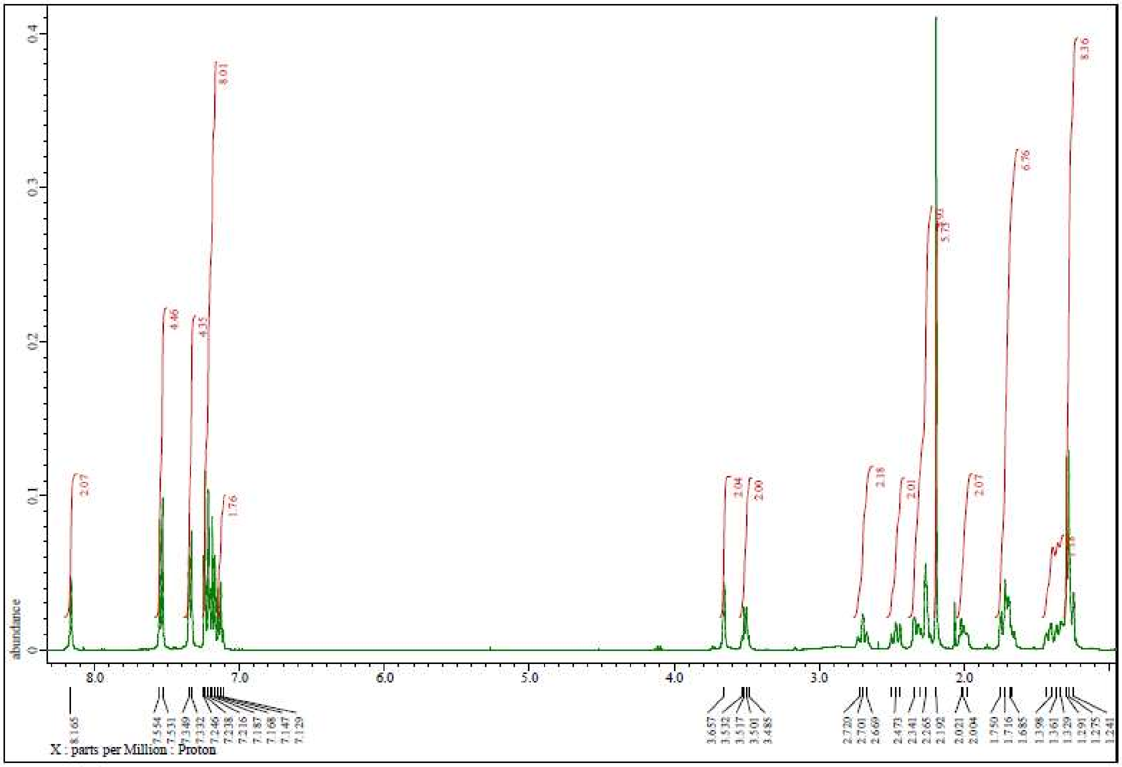

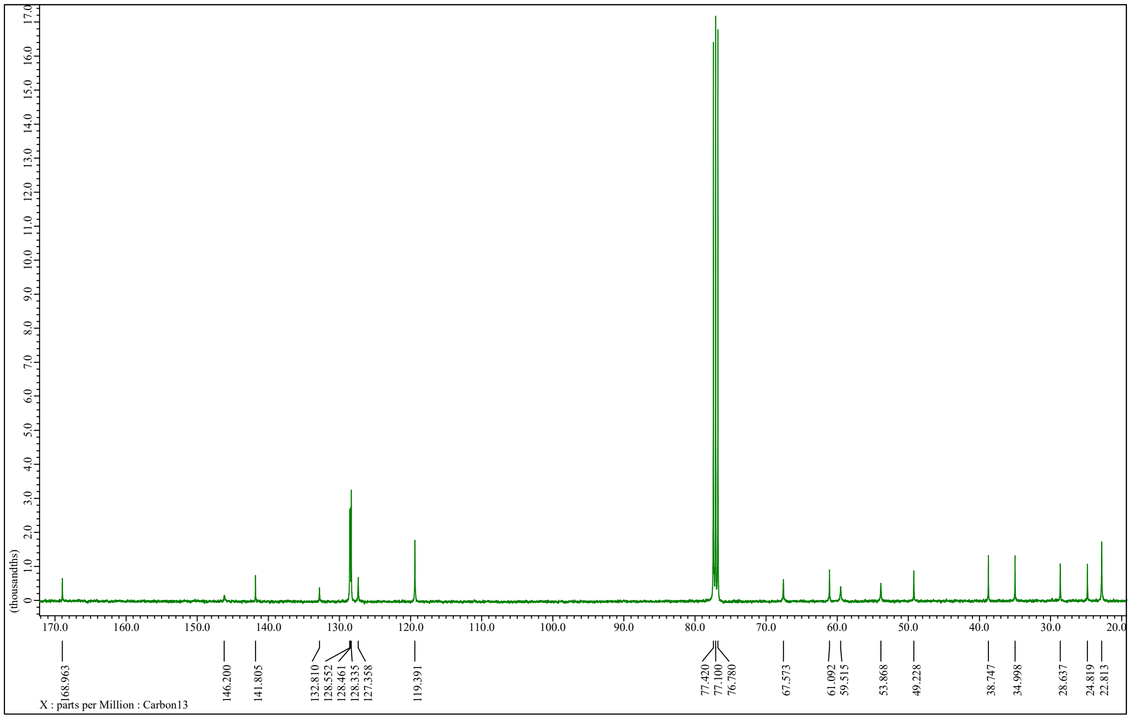

***N*-Bis-(((1*S*,3*R*,4*R*)-2-((*S*)-1-phenylethyl)-2-azabicyclo[2.2.1]heptan-3-yl)methyl)-[1,1'-biphenyl]-4-sulfonamide (13a)**

White solid. Yield 0.55g (83%). Mp 85-89 ºC $[\alpha]_D^{20}$ = +80.7 (*c* 0.7, CH_2Cl_2). ^{1}H NMR (400 MHz, $CDCl_3$): δ 1.22-1.24 (d, *J* = 6.4 Hz, 6H), 1.30-1.37 (m, 3H), 1.44 (s, 1H) 1.49 (s, 2H), 1.56-1.64 (m, 2H), 1.68-1.70 (m, 2H), 1.90-1.97 (m, 4H), 2.14 (s,1H), 2.17-2.20 (m, 2H), 2.30-2.31 (m, 2H), 3.43-3.48 (q, *J* = 6.4 Hz, 2H), 3.58 (s, 2H), 7.02-7.04 (m, 2H, ArH), 7.16-7.34 (m, 10H, ArH), 7.40-7.51 (m, 5H, ArH), 7.57-7.59 (m, 2H, ArH) ppm.

Solvents peaks: chloroform 7.24 (s) ppm.

^{13}C NMR (125 MHz, $CDCl_3$): δ 22.8, 23.4, 28.4, 35.1, 39.0, 53.5, 59.3, 61.2, 67.1, 127.0, 127.2, 127.8, 128.1, 128.2, 128.4, 129.0, 129.6, 136.1, 139.9, 144.6, 146.8 ppm.

Solvents peaks: chloroform-*d* 77.0 ppm (t).

IR (KBr): 3435, 2969, 2870, 1595, 1492, 1453, 1344, 1307, 1165, 733, 700, 667, 587 cm^{-1}. HRMS (ESI+, *m/z*): calcd for $[C_{42}H_{49}N_3O_2S]^+$ ($[M+H]^+$): 660.3618; found 660.3619.

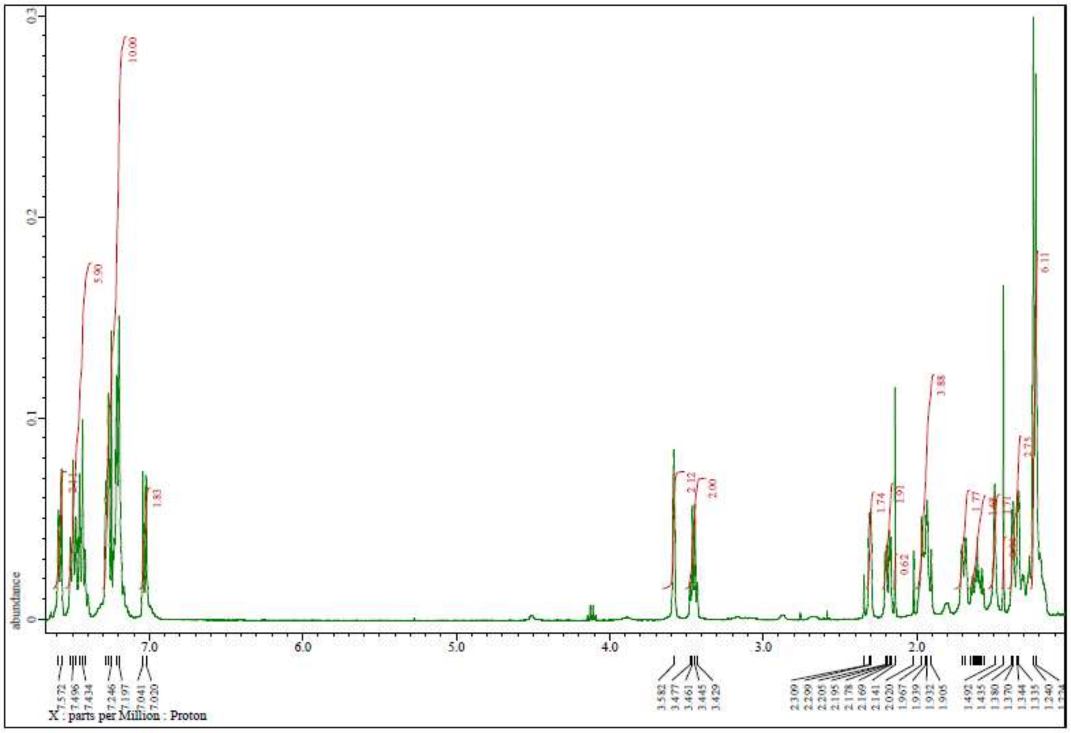

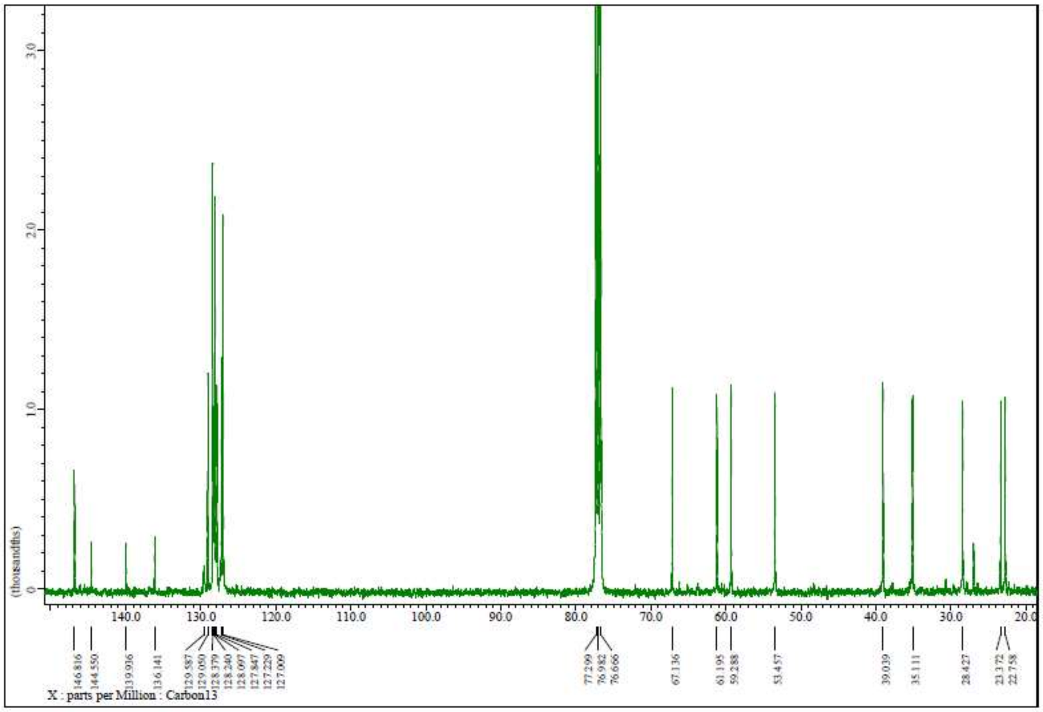

(thousandths)
3.0
2.0
1.0
0
140.0
130.0
120.0
110.0
100.0
90.0
80.0
70.0
60.0
50.0
40.0
30.0
20.0
146.816
144.550
139.936
136.141
129.587
129.050
128.379
128.240
128.097
127.847
127.229
127.009
77.299
76.982
76.666
67.136
61.195
59.288
53.457
39.039
35.111
28.427
23.372
22.758
X : parts per Million : Carbon13
